# Strategies for Enhancing Selectivity in Anticancer Metal Complexes

**DOI:** 10.1021/acsomega.5c11333

**Published:** 2026-05-22

**Authors:** Paolo R. Butcher, Daniel Sykes

**Affiliations:** School of Human Sciences, 4904 London Metropolitan University, 166−220 Holloway Road, London N7 8DB, U.K.

## Abstract

Anticancer metal complexes have always been a key aspect of chemotherapy. Platinum complexes such as cisplatin are commonly used. However, their lack of selectivity often leads to severe side effects. This review examines strategies that have been used to enhance the selectivity of metal complexes toward cancer cells and therefore, minimize toxicity toward healthy cells. These strategies have been categorized into passive targeting, active targeting, stimulus-responsive, structural modifications, subcellular targeting, delivery systems, external stimulus-controlled methods, and exploitation of tumor-specific biology. Each section discusses key scientific principles behind how the strategy works, examples, advantages, limitations, and recent advances or proposed methods for improvement. This Review aims to provide a consolidated resource for researchers aiming to design next-generation metal complexes with improved selectivity.

## Graphical abstract

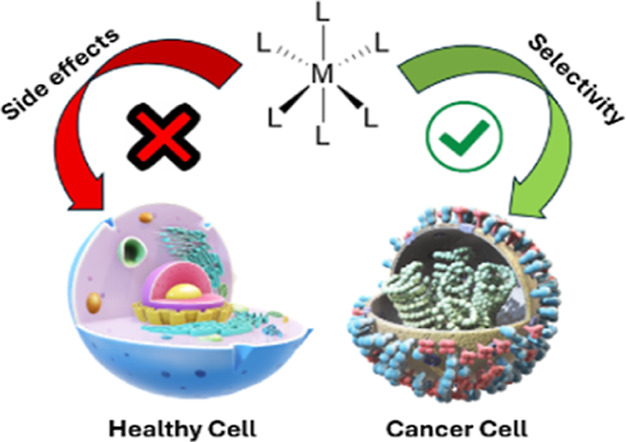

## 1 Introduction

### 1.1 Background

Metal complexes have played a crucial role in the development of anticancer chemotherapy.^1^ Cisplatin and its analogues^2^ have been the main compounds in metal-based therapeutic drugs (Figure 1) since their approval in the 1970s.^3^ Despite their wide use and proven effectiveness, these platinum-based drugs often have serious issues. This includes systemic toxicity, severe side effects,^4^ and the development of drug resistance.^5^ These issues primarily come from a lack of selectivity. Traditional metal complex drugs tend to affect both cancer and healthy cells, leading to off-target damage.^6^

**Figure 1:**
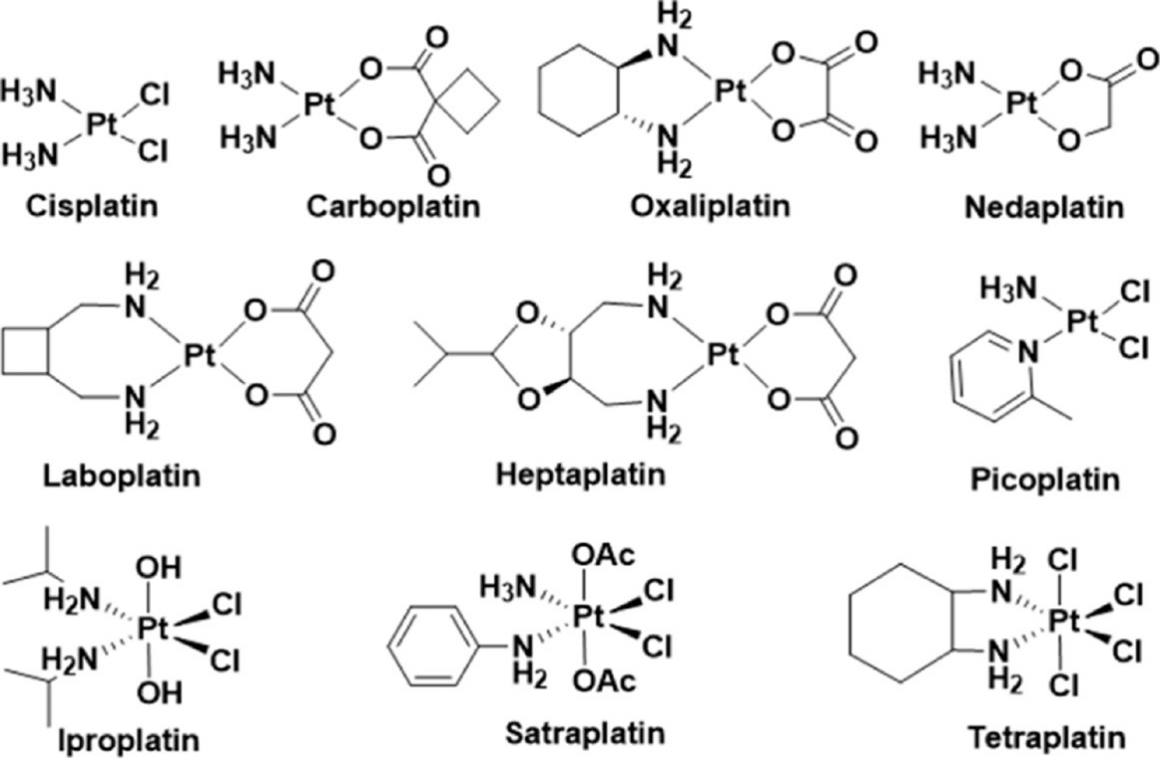
Structures of cisplatin and its analogues **(Ghosh, 2019)**.^2^ Adapted/redrawn using ChemDraw based on literature from ref 2.

### 1.2 Shift in Focus to Prioritize Selective Medicine

Over the past two decades, increasing efforts have been made to design anticancer metal complexes that have enhanced selectivity for cancer cells to reduce damage to normal tissues. A few examples include using photodynamic therapy (PDT) (Figure 2), targeting moieties (Figure 3), and nanoparticles for drug delivery (Figure 4).^7–9^

**Figure 2:**
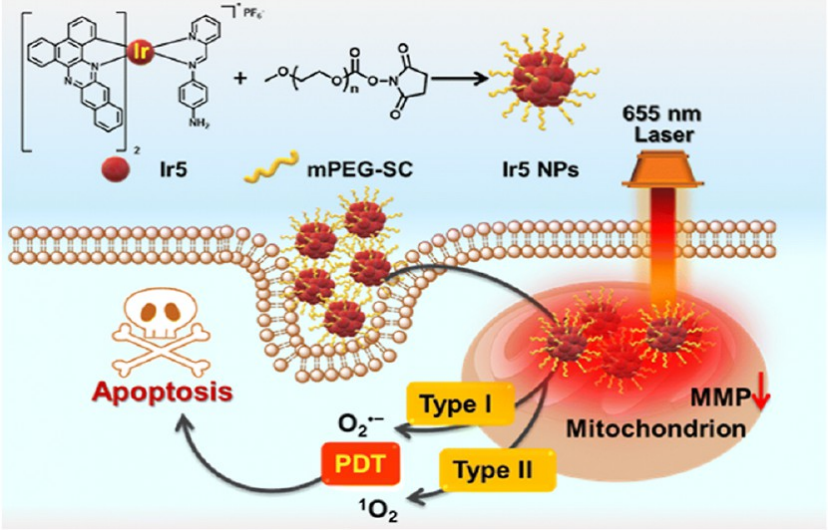
Iridium (Ir) complex used in PDT, generating reactive oxygen species (ROS), causing apoptosis (cell death) **(Liu** et al.**, 2024).** ^10^ Reproduced with permission from ref 10. Copyright 2024 Royal Society of Chemistry. Creative Commons BY attribution 3.0 license.

**Figure 3:**
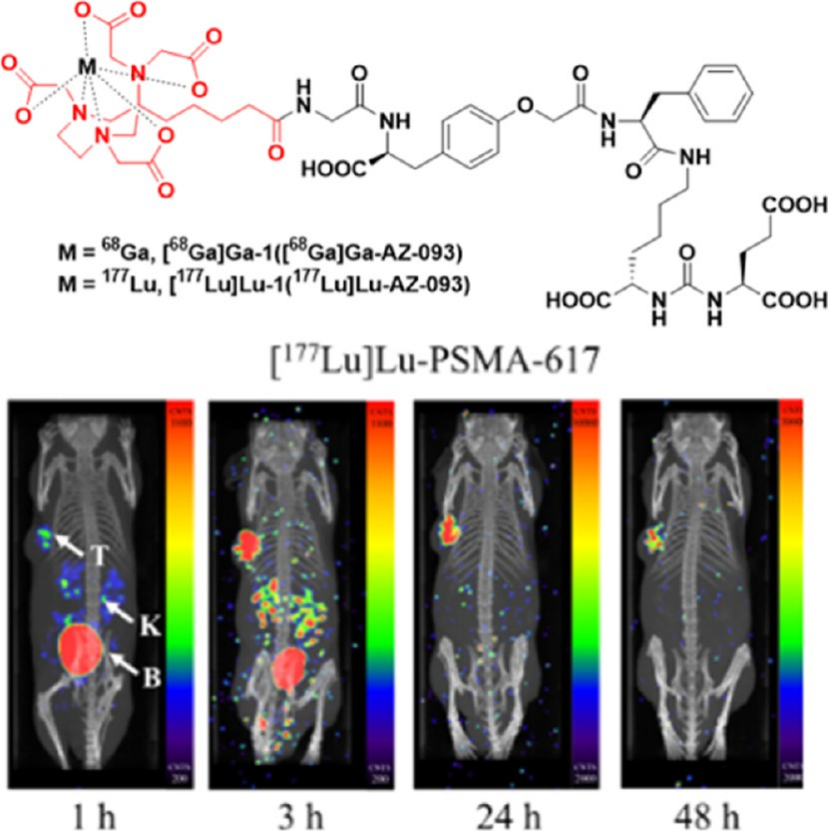
Metal complexes equipped with glutamic acid-urea-lysine (GUL) PSMA (Prostate-Specific Membrane Antigen)-targeting peptide causing tumor shrinkage in mice **(Wang** et al.**, 2024)**.^11^ Adapted from ref 11. Copyright 2024 American Chemical Society.

**Figure 4:**
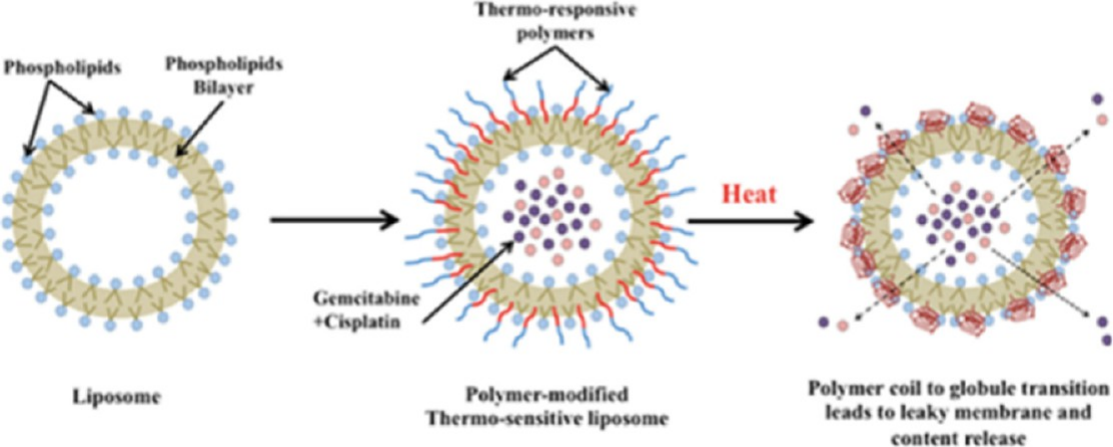
Liposome nanoparticles used for drug delivery **(Emamzadeh, Emamzadeh, and Pasparakis, 2019).** ^12^ Reproduced from ref 12. Copyright 2024 American Chemical Society.

These strategies reflect the shift in anticancer therapy toward selective medicine, where metal complexes are designed to exploit specific features of cancer biology. Transition metals such as iridium (Ir), ruthenium (Ru), and gold (Au) have gained significant attention for their coordination chemistry, tunable oxidation states, and the potential use in selective or stimulus-responsive anticancer drugs.^13^

### 1.3 Brief Overview of Selectivity Method Principles

Selectivity in metal complexes can be achieved through a variety of methods, which can be broadly categorized as passive targeting,^14^ active targeting,^15^ stimulus-responsive activation,^16^ subcellular localization,^17^ and delivery systems.^18^ Passive targeting exploits the enhanced permeability and retention (EPR) effect (Figure 5) of tumor vascularity,^19^ allowing nanostructures or macromolecular complexes to accumulate preferentially in tumor tissues. Active targeting, on the other hand, involves the functionalization of metal complexes with ligands that bind selectively (Figure 3) to overexpressed receptors on cancer cells.^20^ Additional methods include the design of complexes that respond to the tumor-specific microenvironment. This includes complexes sensitive to pH, redox potential, enzyme expression, or hypoxia (low oxygen) (Figure 6),^21^ as well as those that exploit organelle-specific localization, such as mitochondria (Figure 7), DNA, or lysosomes.^22^

**Figure 5:**
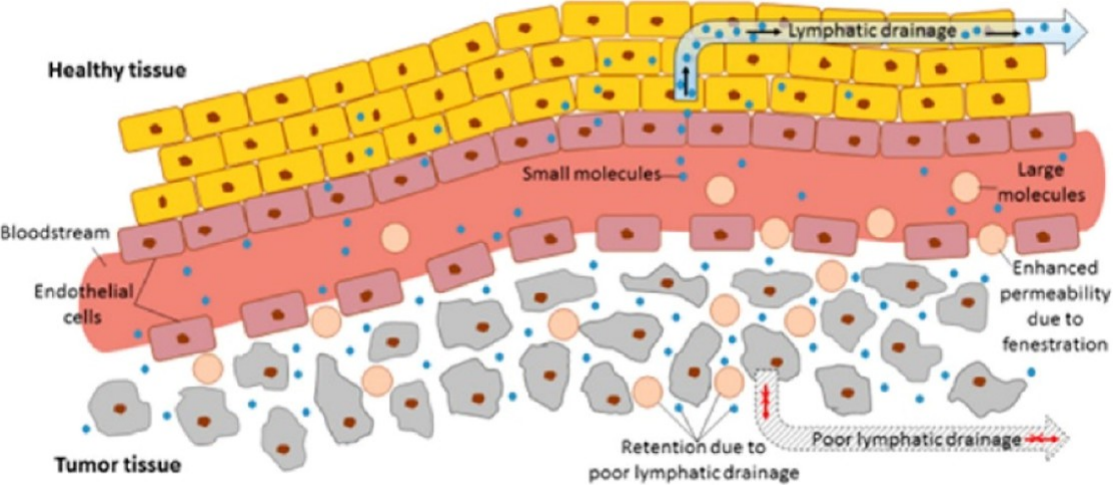
The EPR effect of leaky tumor vasculature **(Mannancherril and Therrien 2018).** ^19^ Reproduced from ref 19. Copyright 2018 American Chemical Society.

**Figure 6:**
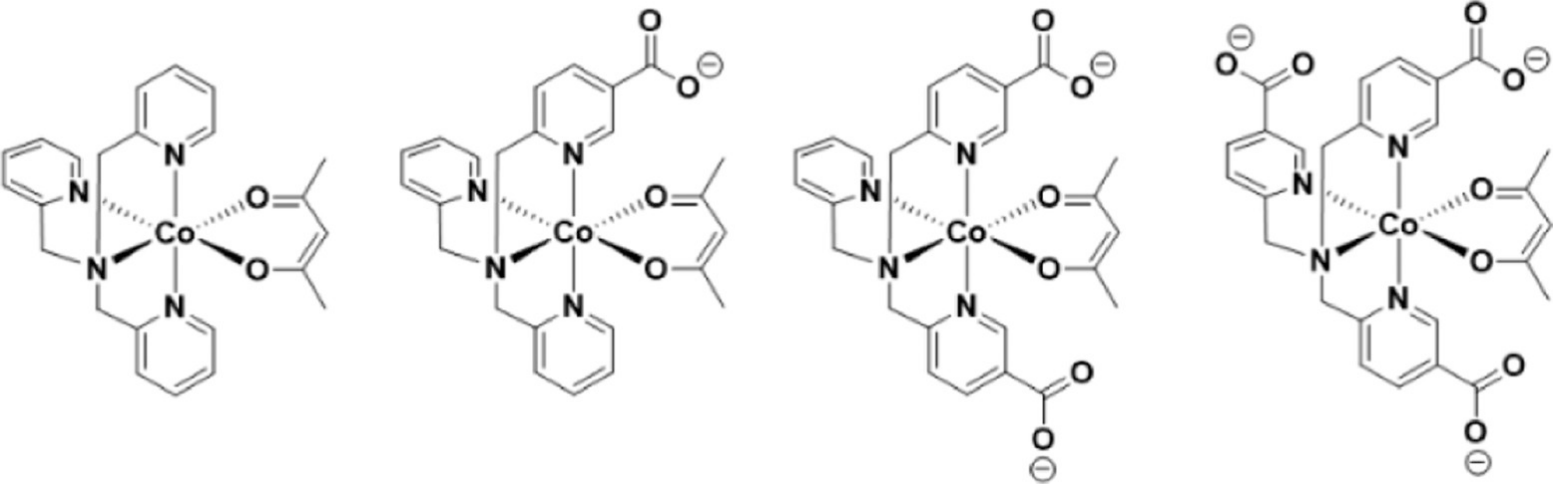
Cobalt (Co) complexes with redox sensitive carbonyls that can be used for detection of hypoxic tumor microenvironments via a change in the absorption and emission signal wavelengths of light **(O’Neill** et al.**, 2017).** ^21^ Adapted from ref 21. Copyright 2017 American Chemical Society.

**Figure 7:**
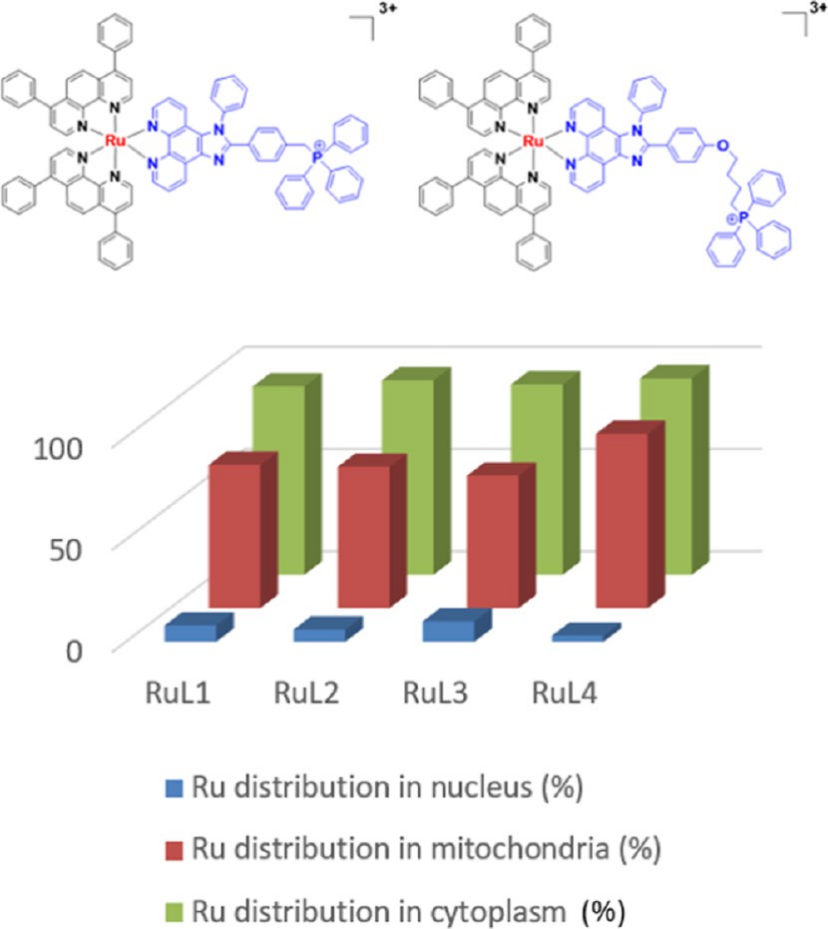
Ru complexes with TPP favoring localization in mitochondria over nucleus **(Liu** et al.**,2015)**.^22^ Adapted/redrawn with ChemDraw Software and Excel based on the literature from ref 22.

Furthermore, chemical modifications of ligands, manipulation of the overall complex charge,^23^ and DNA structure-specific binding offer additional ways to refine selectivity. Delivery systems, including liposomes, dendrimers, and other nanoparticles (Figure 8),^24^ can further improve selectivity by improving pharmacokinetics and enabling controlled or triggered drug release. External stimuli such as light or magnetic fields can activate or control magnetic nanoparticles with spatial and temporal precision.^25^ Staying on the main topic of metal complexes rather than nanoparticles, this review will cover liposomal polymeric, dendrimeric, and metal–organic nanocarriers specifically to offer a general insight into how nanoparticles can be used to deliver metal complexes.

**Figure 8:**
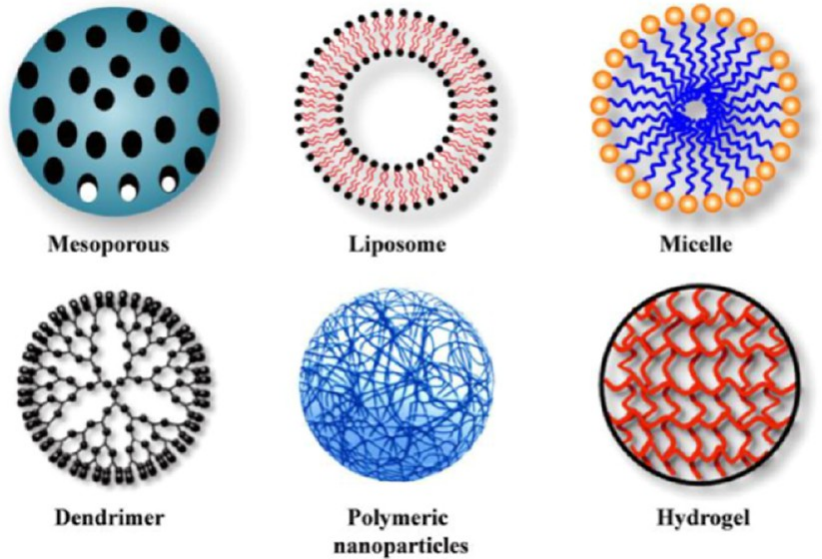
Six major types of nanoparticles used in drug delivery **(Graham** et al.**, 2025).** ^26^ Reproduced from ref 26 under a Creative Commons Attribution (CC BY) license.

### 1.4 Review Article Purpose

This Review aims to provide an overview of the different strategies that have or can be used to enhance the selectivity of anticancer metal complexes. Each method is systematically broken down, showing examples, underlying mechanisms, advantages, limitations, and potential for passing clinical trials.

By consolidating developments across these interdisciplinary metal complex treatments for cancer, this should provide a useful resource for design of next-generation anticancer complexes with improved selectivity toward cancer cells, less toxicity to healthy cells, fewer side effects, and higher survival rates of therapy.

**Explanation of Scientific Principles Behind the Strategies Used to Make Metal Complexes Selectively Target Cancer Cells and Tumors**

## 2 Passive Targeting

### 2.1 Enhanced Permeability and Retention (EPR) Effect

The EPR effect is a key biological feature of solid tumors. It involves leaky vascularity and faulty lymphatic drainage.^27^ This abnormal feature allows nanoparticles and macromolecules (that are larger than 40 kDa and up to 800 kDa) to accumulate passively in tumor tissue while also being retained due to the poor lymphatic clearance.^28^

Metal complexes can exploit the EPR effect when placed inside nanoparticles. For example, liposomes carrying cisplatin (lipoplatin) enhance tumor accumulation and reduce toxicity to the kidneys by taking advantage of passive diffusion through the leaky tumor vascularity.^29^ Similarly, ruthenium complexes loaded into polymeric nanoparticles (Figure 9) have also been shown to accumulate selectively in tumor tissue via EPR.^30^

**Figure 9:**
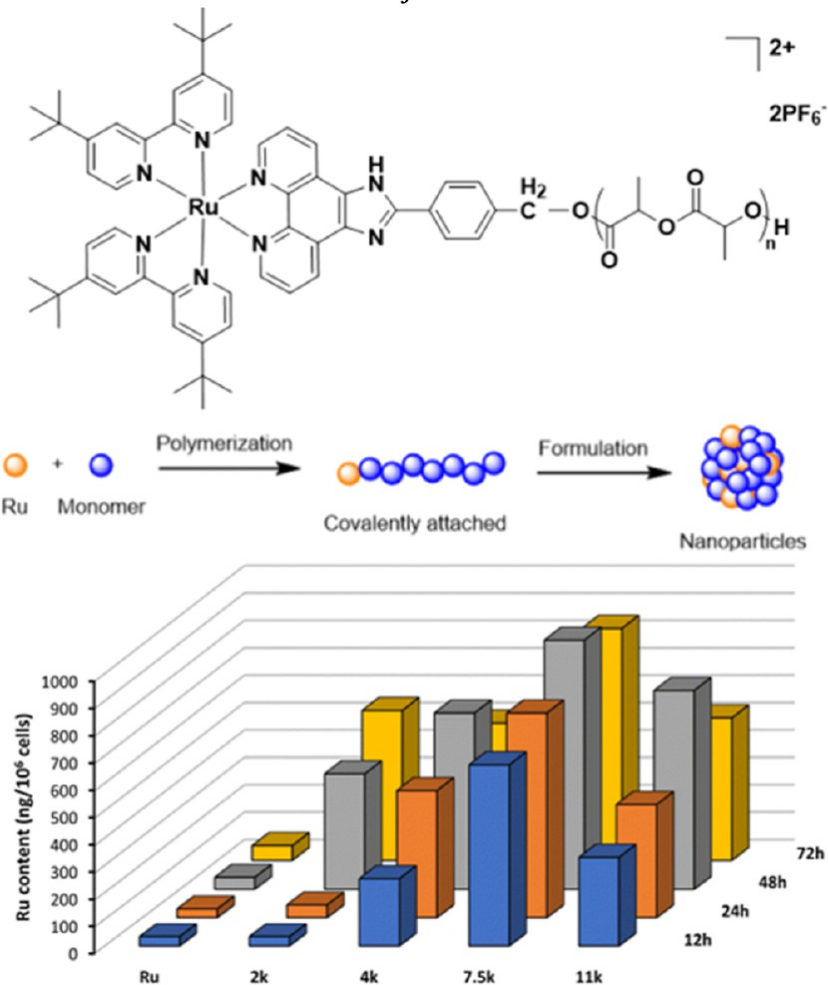
Ru complex accumulation in cancer cells increasing with time by exploiting the EPR effect with polymeric nanocarriers **(António** et al.**, 2023).** ^30^ Adapted with permission from ref 30. Copyright 2023 Royal Society of Chemistry. Creative Commons Attribution-NonCommercial 3.0 Unported License.

The passive nature of this process means it does not require any specific tumor biomarkers like how active targeting methods do. Accumulation of the drug in the tumor is improved, and systemic toxicity is reduced. However, the level of EPR effect exhibited by tumors in humans may not be as pronounced as in mice due to different human tumors varying in vascularity patient to patient.^31^ Due to the diffusion being passive, there is less of a strong driving force for the drug to accumulate in tumor tissue in comparison to active targeting strategies. The diffusion is slow/limited (Figure 10).^32^

**Figure 10:**
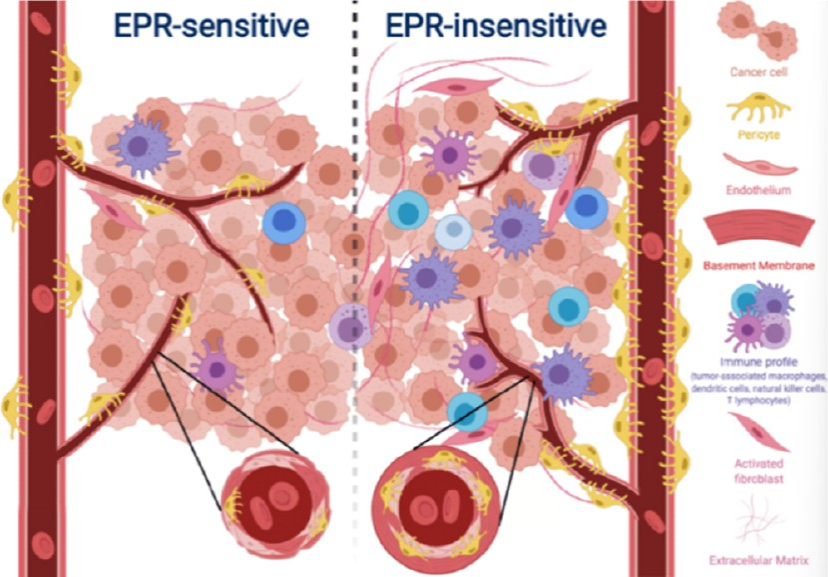
A comparison between EPR-sensitive tumors that have poor vasculature/lymphatic drainage and insensitive tumors that have less exploitable EPR effect **(Dhaliwal and Zheng, 2019).** ^33^ Reproduced with permission from ref 33. Copyright 2019 PubMed Central. Creative Commons Attribution License.

Recent advancements to enhance the EPR effect include dilation of blood vessels via hyperthermia or using modified nanopeptides such as bradykinin (in combination therapy with metal complexes) that widen tumor veins (Figure 11).^34^

**Figure 11:**
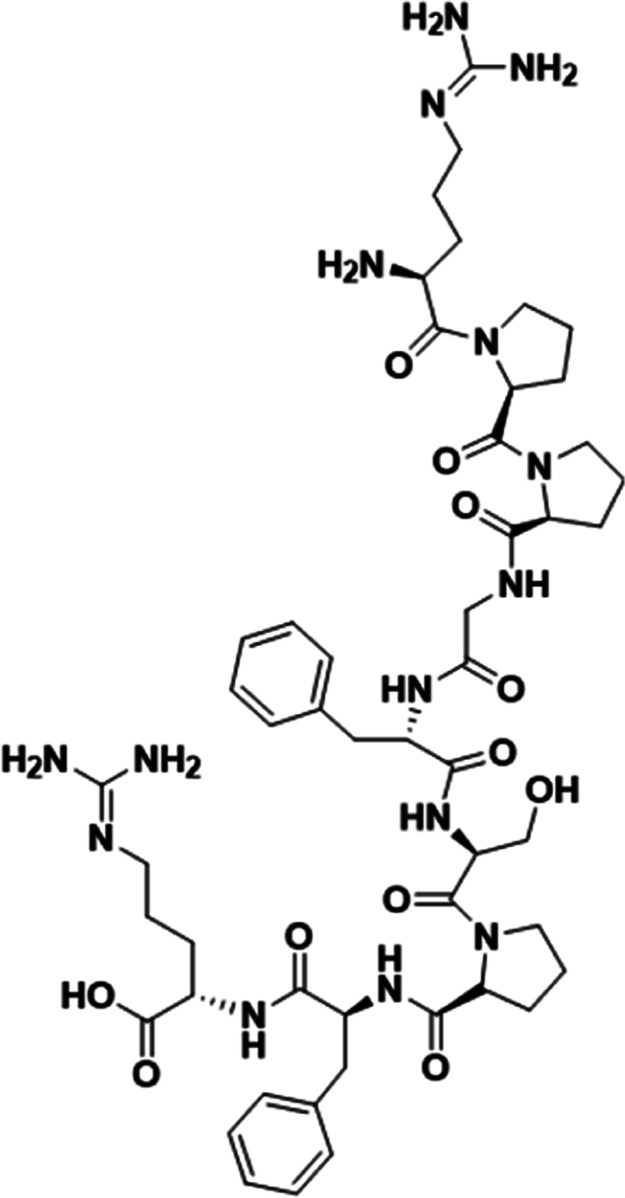
Chemical structure of a Bradykinin peptide that widens blood vessels for better diffusion of drugs **(PubChem 2016).** ^35^ Redrawn using Chemdraw based on the literature from ref 35.

### 2.2 Size Optimization

Small metal complexes (less than 5 nm) are often rapidly cleared by the kidneys, while those that are in the 10–100 nm range can circulate in the bloodstream for longer and better accumulate in tumor tissues through passive targeting.^36^

Platinum­(IV) complexes with hydrophobic ligands or polymer chains can act as nanoclusters,^37^ and iridium­(III) complexes with macromolecular or amphiphilic (both hydrophobic and hydrophilic) ligands (Figure 12) have been used to exploit the EPR effect.^38^

**Figure 12:**
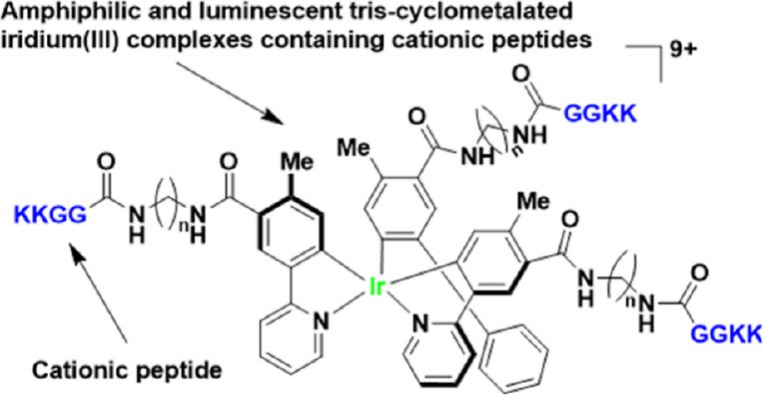
Amphiphilic iridium­(III) complex that can be used to exploit EPR effect **(Hisamatsu** et al.**, 2015).** ^38^ Adapted from ref 38. Copyright 2015 American Chemical Society.

Overall, the size optimization improves the blood circulation time and allows for more significant exploitation of the EPR effect. However, the metal complexes could become less potent, too kinetically stable, and less water-soluble when size is increased. Metal complexes over 200 nm in size may be filtered out of the liver and spleen. Stability in circulation while preserving controlled release remains challenging.^39^

### 2.3 PEGylation

Polyethylene glycol (PEG) chains have been attached to ligands to improve the water solubility of larger metal complexes. This reduces recognition by the immune system and increases blood circulation time which allows more opportunities to exploit the EPR effect of a tumor.^40^

PEGylated oxaliplatin derivatives have shown improved pharmacokinetics and reduced rate of excretion by the kidneys.^41^ Similarly, PEG-functionalized ruthenium­(II) arene complexes have demonstrated enhanced aqueous solubility and stability in the body, allowing improved passive tumor targeting (Figure 13).^42^

**Figure 13:**
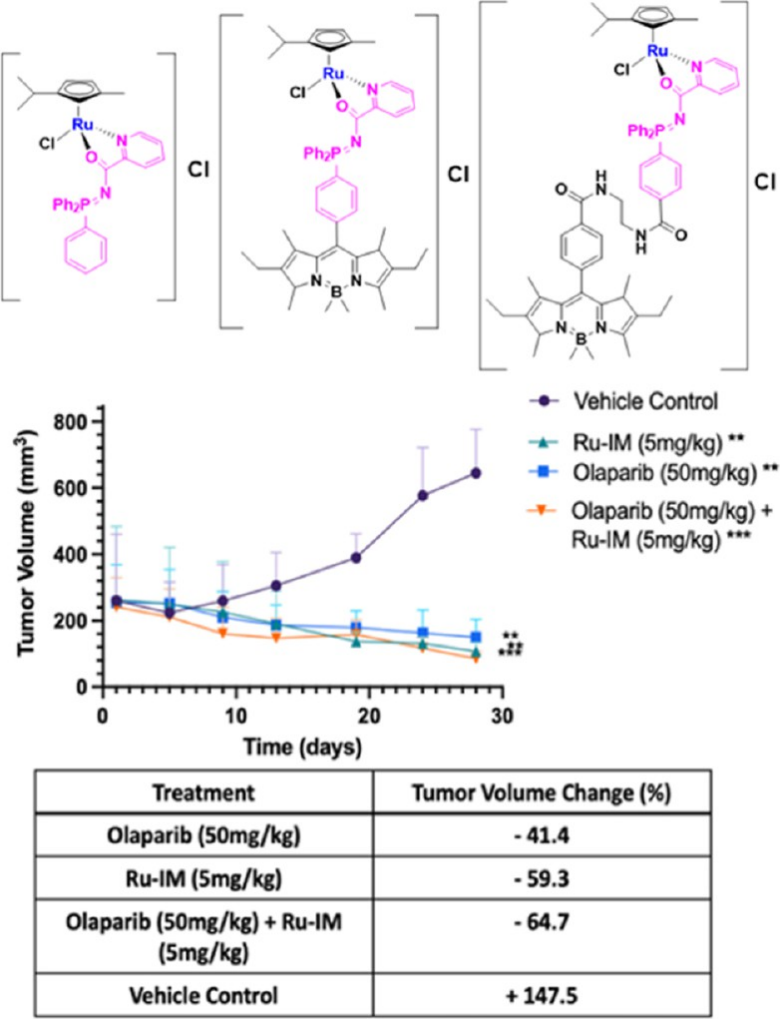
Ru (II) arene complexes causing significant decrease in tumor volume across 30 days, with the potential to be PEGlayted **(Nayeem** et al.**, 2024).** ^42^ Adapted from ref 42. Copyright 2024 American Chemical Society.

However, PEG could mask therapeutic effects if positioned incorrectly and can reduce uptake into the cell due to PEG being hydrophilic and cell membranes being hydrophobic.^43^

This issue can be resolved by using cleavable PEG linkers (Figure 14) and spacers that detach in response to the tumor-associated microenvironment (low pH or elevated glutathione and NADPH). This restores the complex’s cytotoxic activity after accumulation inside the tumor resulting in targeted treatment.^44^

**Figure 14:**
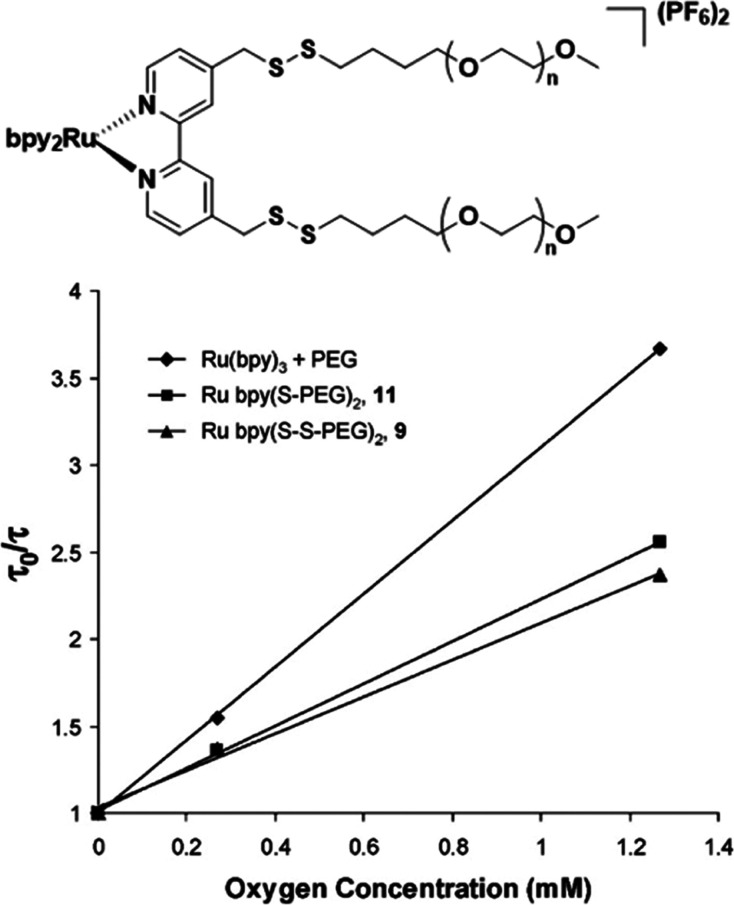
Ru complex with cleavable PEG linkers caused S–S bond to remain stable under increased oxygen concentration allowing activation in a hypoxic tumor environment primarily **(Fiore** et al.**, 2008).** ^44^ Adapted from ref 44. Copyright 2008 American Chemical Society.

## 3 Active Targeting

### 3.1 Ligand–Receptor Targeting

Ligand–receptor targeting involves attaching a biomolecular ligand such as a vitamin, sugar, or small peptide to a metal complex.^45^ These ligands are chosen to recognize and bind to receptors overexpressed on cancer cells (folate receptor accepts B vitamin folate used for DNA synthesis, transferrin receptor accepts iron for oxygen transport). Upon binding, the complex is often accepted by the cell membrane, enhancing selectivity and uptake into the cell.^46^

Platinum­(IV) complexes have been conjugated with folic acid ligands, exploiting the high expression of folate receptors in tumors.^47^ This gives the drug cancer-specific accumulation and reduced off-target effects. Transferrin-conjugated ruthenium complexes target cells with elevated transferrin receptor expression, such as fast-growing, treatment-resistant brain cancers.^48^ Glucose-functionalized ligands^49^ (Figure 15) have also been investigated to target the Warburg effect (Figure 16) (cancer cells use energy from break down of glucose rather than using energy released from NADPH/FADH electrons transferring to oxygen)^50^ and exploit glucose transporter overexpression.

**Figure 15:**
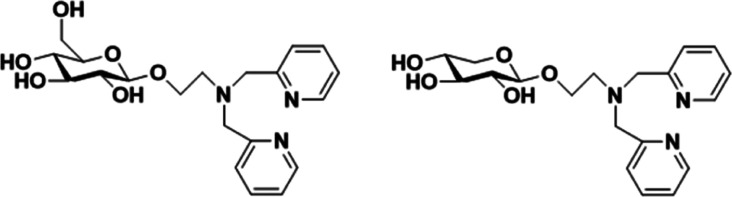
Glucose-functionalized ligands used in metal complexes to target overexpressed glucose receptors in tumors **(Storr** et al.**, 2005).** ^49^ Reproduced from ref 49. Copyright 2005 American Chemical Society.

**Figure 16:**
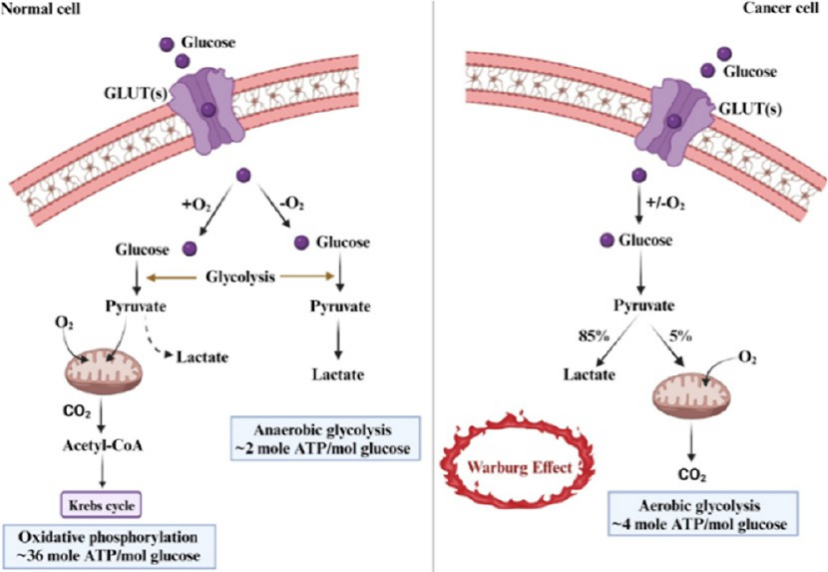
The Warburg Effect explained. A cancer cell’s preference of using glycolysis over oxidative phosphorylation **(Akter** et al.**, 2024).** ^50^ Reproduced from ref 50. Copyright 2024 American Chemical Society.

Advantages include high selectivity for cancer cells that overexpress certain receptors, efficient uptake via natural pathways (endocytosis), and reduced toxicity to normal tissues (which have lower receptor expression).

However, once again, due to tumor behavior varying patient to patient, the overexpression of folate and transferrin receptors may not be as pronounced. Ligands can degrade in the body and, unfortunately, using PEGylation to combat this may sterically hinder the ligand’s ability to bind to receptors.^51^

Recent studies have looked at using multiple receptor-targeting ligands, dual receptor targeting (complexes with ligands targeting multiple receptor types), and using tumor-sensitive linkers or spacers such as disulfide bridges.

### 3.2 Antibody-Conjugated Metal Complexes

This strategy uses antibodies that are selective for tumor-specific antigens (like HER2, EGFR, PSMA), that are chemically bonded to metal complexes (Figure 17),^52^ delivering them directly to cancer cells. Once the metal complexes are bound to the cancer cells, they cross the membrane, allowing drug release inside cells.

**Figure 17:**
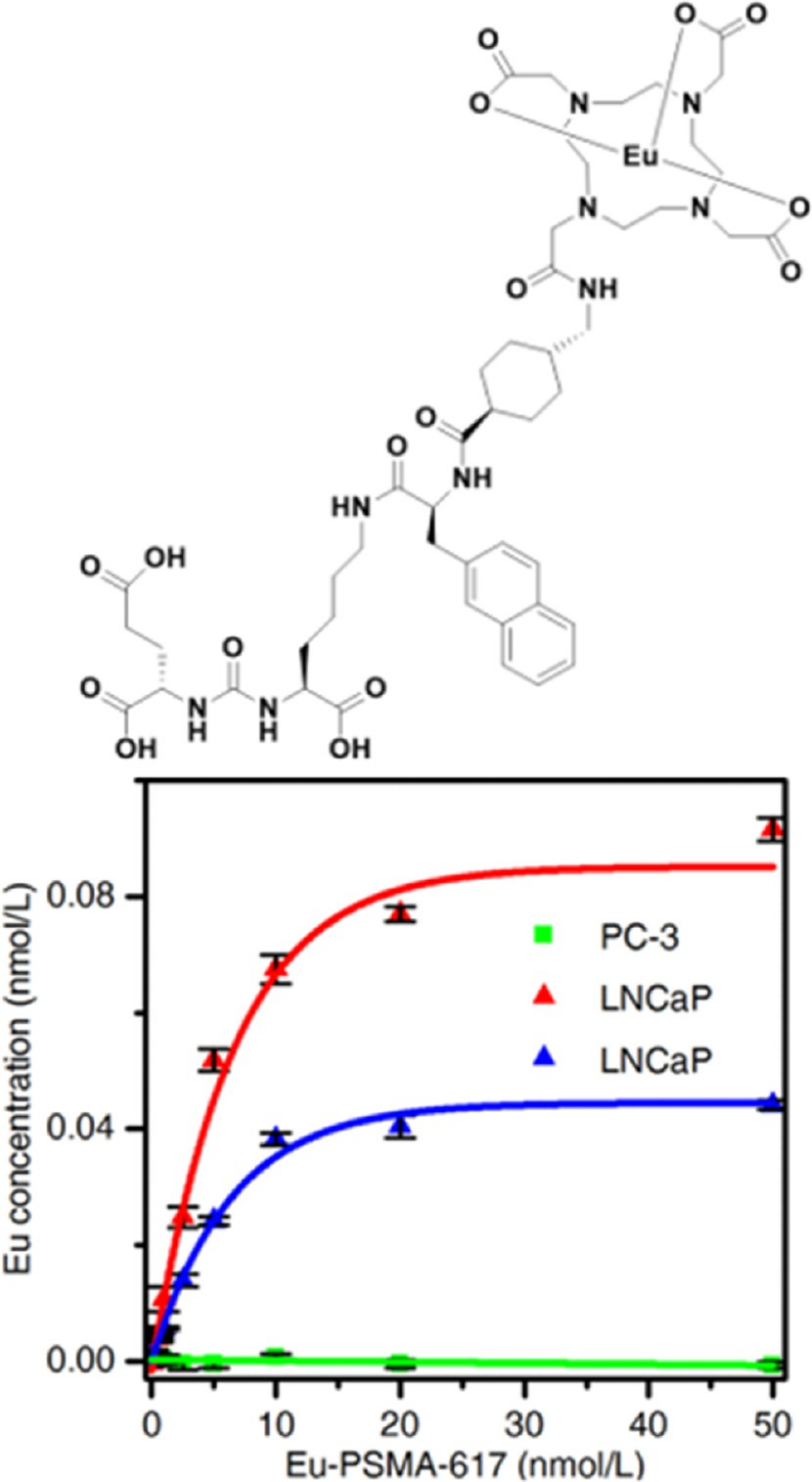
Europium complex that targets PSMA accumulates inside prostate positive cells and does not accumulate inside prostate negative cells **(Holzapfel** et al.**, 2019).** ^52^ Adapted from ref 52. Copyright 2019 American Chemical Society.

Examples include platinum­(IV) complexes that have been conjugated to trastuzumab (an anti-HER2 antibody) for selective delivery to HER2-positive breast cancer cells.^53^ Ruthenium­(II) polypyridyl complexes have been attached to anti-EGFR antibodies (Figure 18),^54^ showing enhanced tumor uptake in the body.

**Figure 18:**
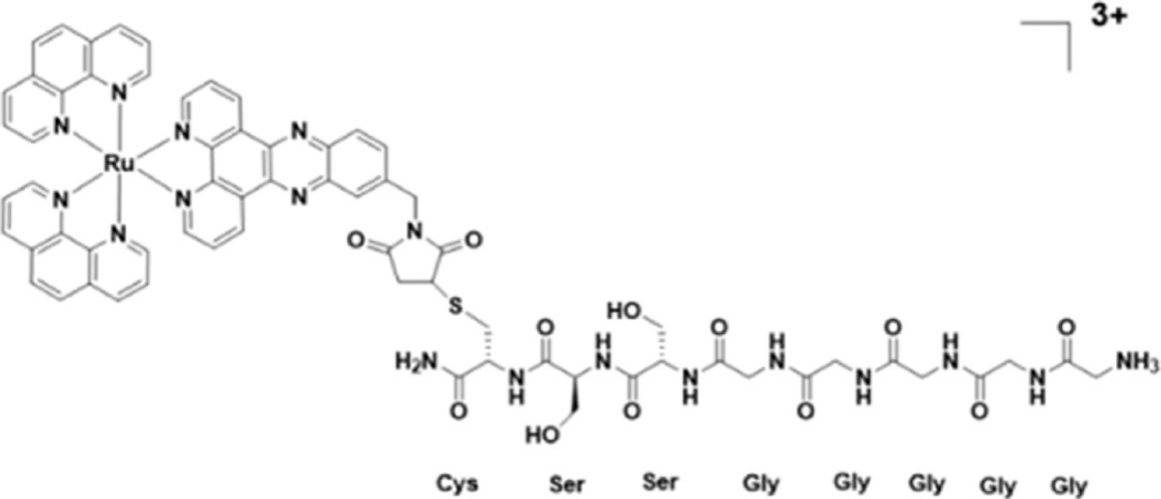
Polypyridyl Ru (II) complex with EFGR targeting **(Karges** et al. **2020).** ^54^ Redrawn using Chemdraw software based on the literature from ref 54. 2020 Wiley.

This enables high selectivity based on antigen recognition, enhances cell uptake, and reduces the toxicity to normal cells. However, the same issues as PEGylation arise with improper conjugation of antibodies interfering with metal complex activity. New developments in site-specific bioconjugation (click and coupling chemistry) allow better control over stoichiometry and positioning of antibodies onto metal complexes.^55^

### 3.3 Peptide Targeting

Peptide targeting utilizes short amino acid sequences that can be conjugated to metal complexes (Figure 19) and bind selectively to receptors that are overexpressed on cancer cells. Commonly targeted receptors include integrins (via RGD peptides), prostate-specific membrane antigen (PSMA) via DUPA or GUL peptides, and other receptors involved in tumor growth and metastasis.^56^

**Figure 19:**
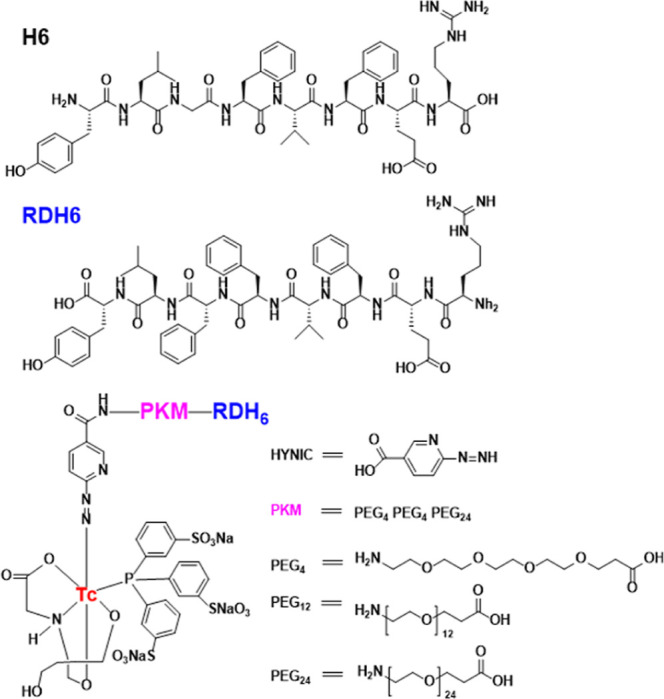
Technetium complex with targeting peptides linked to ligands **(Du** et al.**, 2020).** ^56^ Adapted from ref 56. Copyright 2020 American Chemical Society.

DUPA (2-[3-(1,3-dicarboxypropyl)-ureido]­pentane-dioic acid) and GUL-conjugated ruthenium­(II) and iridium­(III) complexes have been developed for selective targeting of PSMA-expressing prostate cancer cells. GUL targets the PSMA active site by mimicking its substrate (glutamate) with the use of glutamic acid. The urea section mimics a peptide bond and cannot be broken down by the PSMA active site. The urea group forms hydrogen bonds with the active site, allowing the GUL to be engulfed by the cell and, therefore,^57^ the conjugated metal complex too. RGD peptides can be used (Figure 20) to functionalize platinum­(IV) prodrugs and have shown increased binding to integrin-expressing tumor cells. Some peptidyl ligands also incorporate cleavable linkers that respond to tumor environment enzymes, further enhancing selectivity.^58^

**Figure 20:**
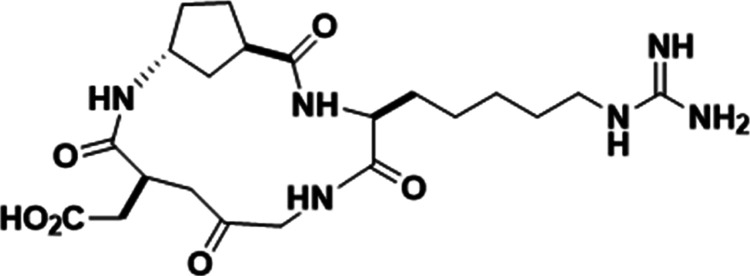
RDG peptide that can be chemically bonded to ligands of metal complexes **(Casiraghi** et al.**, 2005).** ^58^ Adapted from ref 58. Copyright 2005 American Chemical Society.

Peptides are small (exploit EPR effect), less detectable by the immune system (good circulation time), and easy to synthesize (condensation reaction between amino acids).^59^ Limitations include potential degradation of peptides during circulation when detected by enzymes such as peptidases and off-target binding is possible if receptors are expressed on normal tissues.^60^

Recent efforts to combat this include cyclization of peptides, self-assembling peptides, and the use of d-amino acids (mirror image enantiomers of l-amino acids) that are less detectable by peptidases.^61^

### 3.4 Hormonal Targeting

Hormonal targeting involves the conjugation of metal complexes to hormone molecules (or hormone analogues) that bind selectively to hormone receptors overexpressed in hormone-dependent cancers.^62^ Estrogen, androgen, and progesterone receptors are frequently exploited in cancers such as breast, prostate, and ovarian. Once bound, the complex will be taken up, leading to localized toxicity inside the cancer cell.^63^

Estrogen-conjugated platinum­(IV) drugs (Figure 21) have been designed to exhibit selective cytotoxicity toward estrogen receptor-positive breast cancer cells.^64^ Testosterone-modified platinum­(IV) complexes have been shown to selectively target androgen receptor-positive prostate cancers.^65^

**Figure 21:**
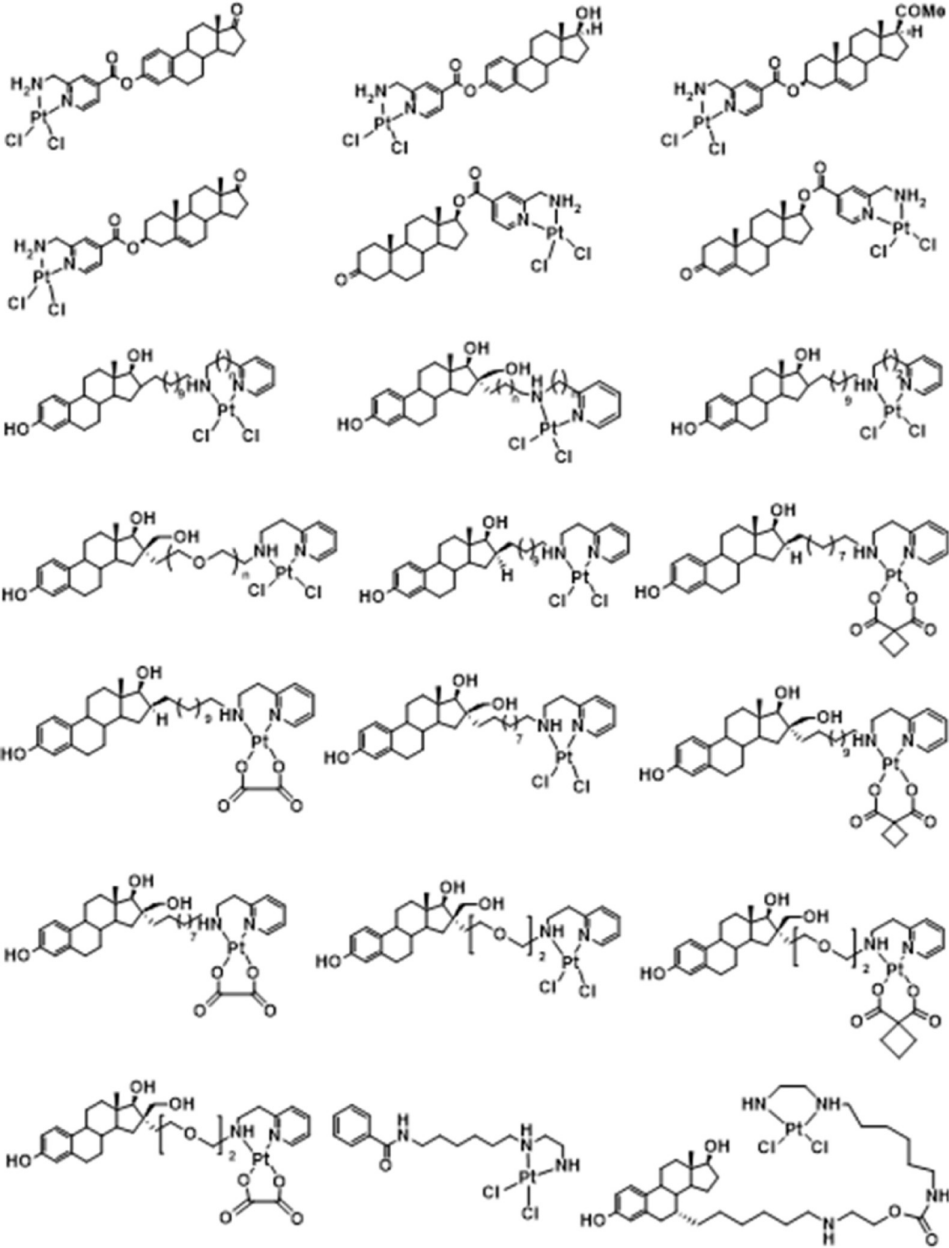
Hormone-conjugated anti breast cancer platinum complexes **(Liang** et al.**, 2023).** ^64^ Adapted from ref 64. Copyright 2023 American Chemical Society.

Downsides involve the risk of normal tissue that is hormone-sensitive growing to abnormal levels, causing side effects such as endocrine disruption. Receptor expression may decrease over time due to tumor evolution or treatment resistance.^66^

Current research explores the use of cleavable hormone linkers and dual-function conjugates that both target and interfere with hormone signaling. Recent estrogen-linked Pt­(IV) drugs demonstrate increased uptake in estrogen receptor-positive breast cancer cells and are now being explored in combination with hormonal therapies like tamoxifen to overcome hormonal therapy resistance.^67^

## 4 Stimulus-Responsive Activation

### 4.1 PH-Sensitive Metal Complexes

Tumor tissues usually have a slightly acidic pH (∼6.5–6.9) compared to normal tissues (∼7.4), due to high dependence on glucose breakdown activity and poor blood flow/oxygen delivery. Organelles like endosomes and lysosomes are even more acidic (pH ∼5.0–6.0).^68^ These pH-sensitive metal complexes are designed to remain inert at a normal tissue pH of ∼7.4 and become activated or release their therapeutic compound structure in acidic environments, therefore enhancing cancer selectivity.^69^

Copper­(II) drugs with acid-labile axial ligands (hydrazone or *cis*-aconitic linkers) are stable in blood but hydrolyze in acidic tumor tissue or endosomes to release the active drug.^70^ Ruthenium­(II) complexes with benzimidazole or imine-based ligands have demonstrated selective activation under acidic conditions.^71^ Some iridium­(III) complexes exhibit pH-dependent changes in photophysical properties, making them suitable for both therapy and pH-responsive phosphorescence imaging (Figure 22).^72^

**Figure 22:**
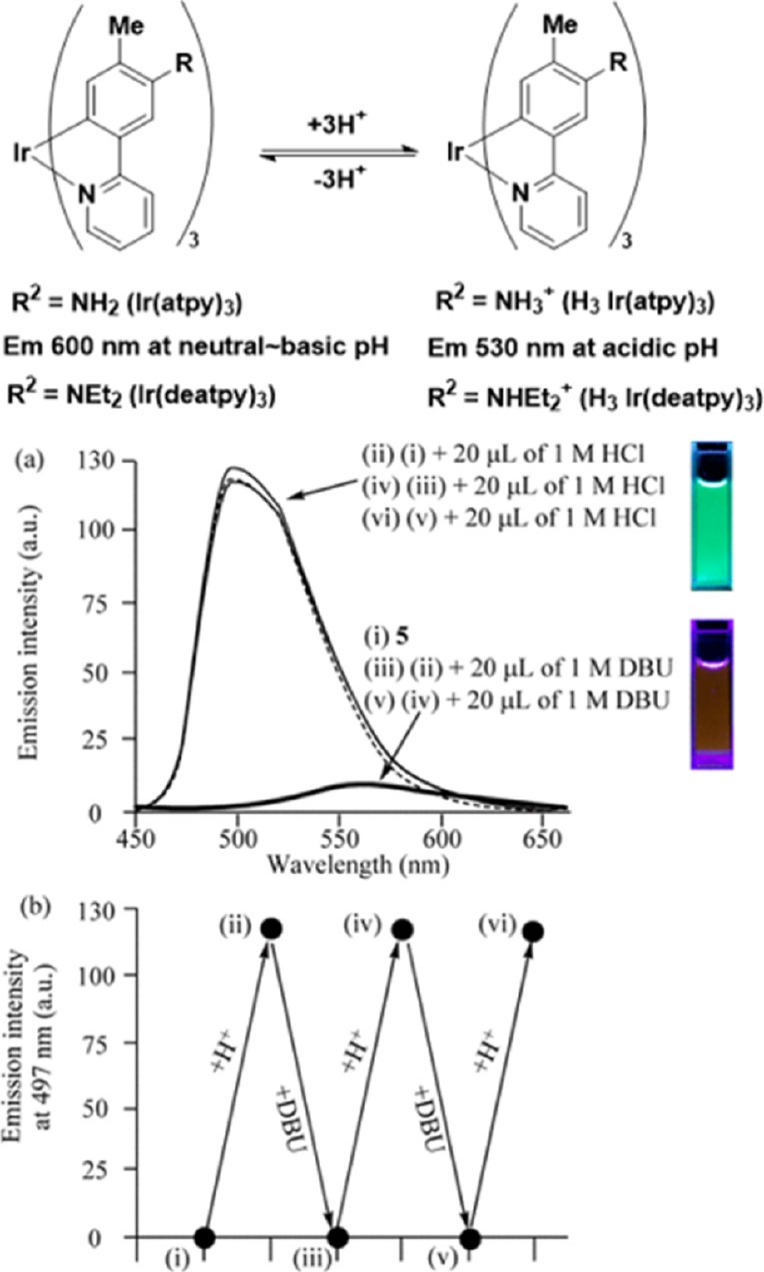
Iridium complex with acidic-sensitive aromatic-substituted ligands. Tumor/endosomes change the emission signal from 600 nm to 530 nm. Increased emission intensity under acidic conditions allows the complex to highlight tumor environments **(Moromizato** et al.**, 2012).** ^72^ Adapted from ref 72. Copyright 2012 American Chemical Society.

Unlike hormonal targeting (treating only hormone-related cancers), pH sensitivity can be used to target all types of tumors. However, the pH difference between tumors and normal tissues is often subtle, so extreme sensitivity is required. There is a risk of premature release in acidic compartments of healthy cells in the liver, for example. Acid-sensitive ligands must remain stable during circulation.^73^

Recent efforts involve the use of dual-triggered systems that rely on pH sensitivity but also in conjunction with either redox or enzyme-sensitive systems.

### 4.2 Redox-Sensitive Systems

The inside of a tumor cell has elevated levels of reducing agents, particularly GSH (glutathione), which is often present at concentrations 100–1000 times higher inside cancer cells than in normal cells.^74^ Redox-sensitive metal complexes are designed to be inactive or stable in oxidizing environments but undergo reduction or ligand cleavage inside the tumor cell, releasing the active metal species or activating its cytotoxic version. As well as tumor-sensitive therapeutics, diagnostics are also possible through redox sensitivity.^13^ Carbonyl groups on ligands can be reduced into hydroxyl groups altering properties from electron withdrawing to electron donating. This alters the HOMO–LUMO gap meaning a change in absorption and emission wavelengths before and after accumulation inside a tumor allowing for detection of tumors.^75^

Platinum complexes are good examples of redox-sensitive drugs as they are reduced by GSH.^76^ Ruthenium­(III) complexes, such as NAMI-A, are reduced in the tumor environment to more reactive Ru­(II) species.^77^ Iridium­(III) complexes containing redox-cleavable sulphonamides (Figure 23)^78^ or disulfide linkers release cytotoxic or photoreactive fragments upon reduction inside the cancer cells.

**Figure 23:**
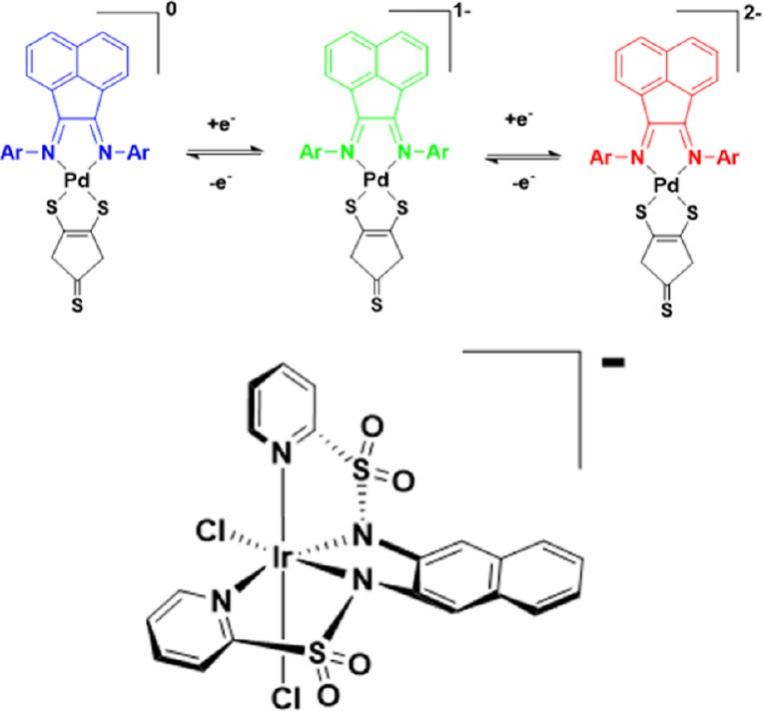
Redox-sensitive Pt/Pd complexes and a redox sensing iridium complex utilizing sulfonamides **(Romashev** et al. **2022).** ^76^ **(Li, M. and Bernhard, S., 2017).** ^78^ Adapted from ref 77. Copyright 2022 American Chemical Society. Redrawn using Chemdraw software based on the literature from ref [Bibr ref79] 2017 Elsevier.

This strategy works relatively well due to the reductive environment being a property of all tumors. The theory of redox Chemistry is simplistic in nature and therefore is easy to add to ligands and the surface of nanocarriers of metal complexes.^79^ However, the level of redox sensitivity can vary between tumors and between stages of tumor progression, reducing activation efficiency in some cases. Redox conditions in inflamed or regenerating healthy tissues may lead to nonspecific activation and a chance of premature reduction during circulation in patients with oxidative stress or inflammation.^80^

Advanced redox-sensitive designs include disulfide-bridged metal complexes and linkers that disassemble upon exposure to GSH. Recent Pt­(IV) complexes with dual-targeting ligands and redox-labile bonds have shown enhanced efficiency and minimal off-target toxicity.^80^

### 4.3 Enzyme-Activated Prodrugs

Certain enzymes that enhance formation of new blood vessels from pre-existing vessels and other processes required for metastasis/rapid growth (matrix metalloproteinases and cathepsins), as well as break down of carbohydrates (β-glucuronidase), are overexpressed or unregulated in the tumor environment or within cancer cells.^81^ Enzyme-activated drugs are designed with cleavable linkers or masking groups that are specifically removed by these enzymes, therefore releasing the active metal complex at the target site.

For example, platinum complexes with ligands target matrix metalloproteinases, which are abundant in metastatic tumors (Figure 24).^82^ Metal complexes can be masked with β-glucuronide (Figure 25) or phosphate groups, which are cleaved by tumor-expressed glucuronidases to unmask DNA-binding moieties.^83^ Some organometallic complexes can be linked to peptide sequences cleavable by cathepsin B, a lysosomal enzyme overexpressed in multiple cancers.^84^

**Figure 24:**
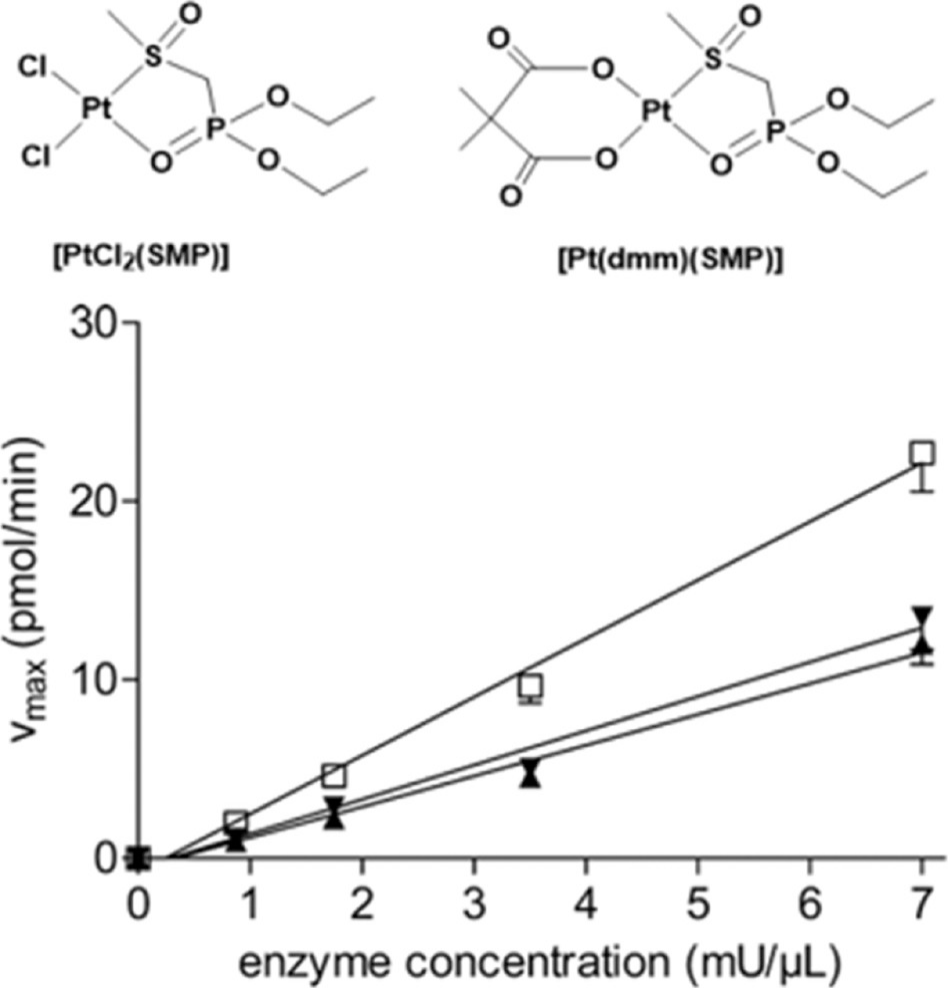
Platinum complexes that target overexpressed metalloproteinase activity in tumors. MMP-12 activity as a function of enzyme concentration in the absence (control, □) and in the presence of [PtCl_2_(SMP)] (▲) or [Pt­(dmm)­(SMP)] (▼) **(Sasanelli, R** et al. **2007).** ^82^ Adapted from ref 83. Copyright 2007 American Chemical Society.

**Figure 25:**
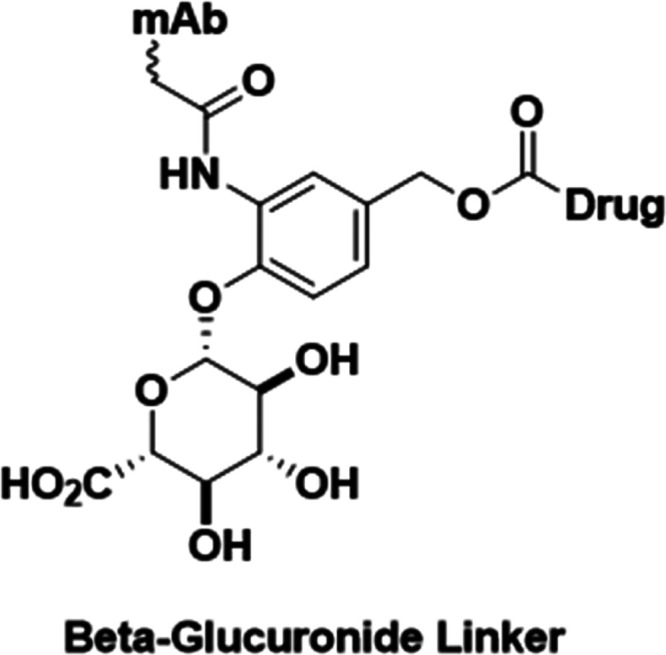
Shows how an anticancer drug can be linked to tumor-expressed glucuronidases targeting moiety **(Jeffrey** et al.**, 2010).** ^83^ Adapted from ref 84. Copyright 2010 American Chemical Society.

This strategy exploits enzymes on the surface (matrix metalloproteinases) and enzymes inside the cell (cathepsins). However, this method has similar limitations to redox-sensitive metal complexes with varying tumors and upregulation of these enzymes in regenerating normal tissues increasing the risk of normal tissue repair being targeted. The cleavable linkers must be stable in circulation yet sensitive at the tumor site.

Recent efforts have focused on dual-enzyme-responsive linkers and multistage metal complexes activated by sequenced enzymatic steps. Metal complexes could be coordinated to cathepsin-sensitive linkers being explored for activation once inside the cell and real-time fluorescence imaging.^85^

### 4.4 Hypoxia-Activated Complexes

Hypoxia (low oxygen) is a key feature of tumors resulting from poor vascularity and rapid cell growth. Hypoxia-activated metal complexes are designed to remain nontoxic or inert under normal conditions but become reduced, cleaved, or activated in oxygen-deprived environments. This allows for selective release of cytotoxic species in the tumor core while sparing healthy tissues.^86^

Co­(III) complexes are hypoxia-activated, as they are reduced to Co­(II) in low-oxygen environments, triggering ligand release.^87^ Iridium­(III) complexes with bioreductive nitroaromatic ligands or azo groups undergo reduction in hypoxic cells, resulting in cytotoxic or phosphorescence activation.^88^

Benefits of this method involve highly tumor-specific treatment, as normal tissues are well oxygenated and the strategy can be used with in combination therapies. Again, this method is limited by tumor variability in oxygen deficiency, and the metal complex could be partially activated by inflamed normal tissues and so the redox potential needs to be carefully tuned.^89^

Recent studies on Ir­(III) complexes conjugated to hypoxia-sensitive azo linkers (Figure 26) have demonstrated high selectivity, controlled activation, and minimal toxicity under normal conditions. Some also use hypoxia-activated fluorescence for imaging of tumors specifically.^90^

**Figure 26:**
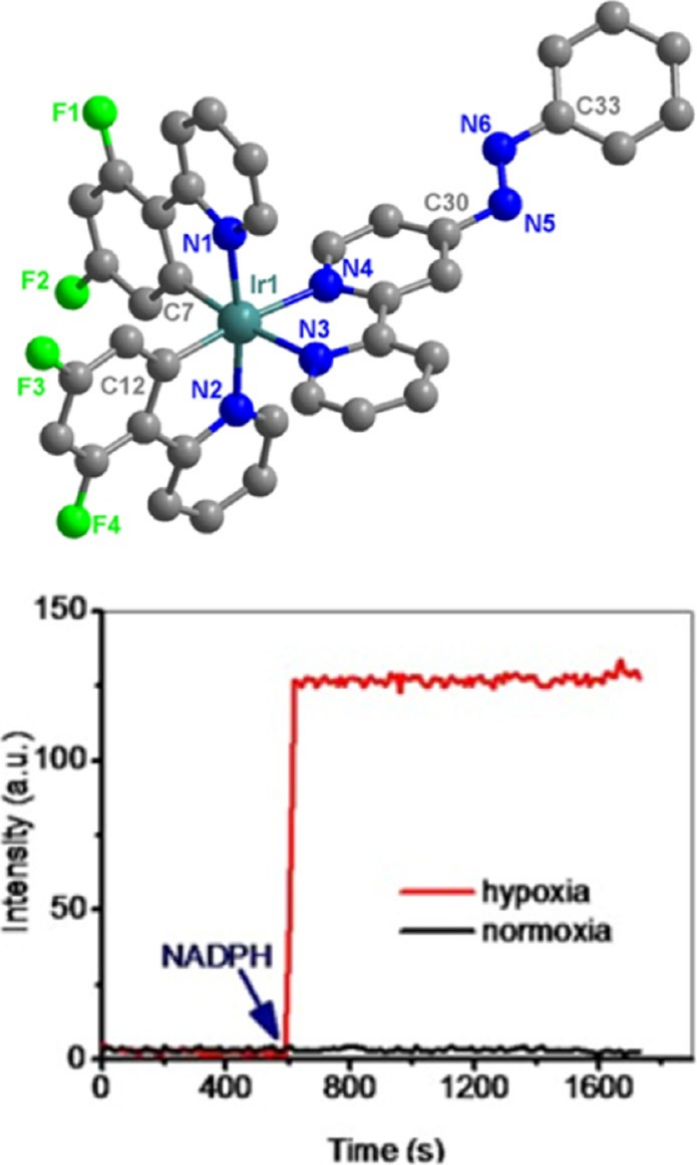
Iridium­(III) complex with a hypoxia-sensitive azo linker exhibiting different phosphorescence intensities under hypoxic and normoxic conditions **(Sun** et al. **2015)**.^90^ Adapted from ref 91 **. Licensed under creative commons CC BY 4.0**.

## 5 Subcellular/Organelle Targeting

### 5.1 Mitochondrial Targeting

Mitochondria have a strong negative inner membrane potential (−150 to −180 mV).^91^ This naturally attracts lipophilic cationic species, making it possible to selectively deliver positively charged metal complexes to mitochondria. Since mitochondria are essential for energy production of a cell, their targeting can trigger cancer cell death more effectively.^92^

Triphenyl phosphonium (TPP) is a widely used mitochondrion-targeting moiety. Conjugation of TPP to ruthenium­(II) or iridium­(III) complexes leads to preferential mitochondrial accumulation. TPP–Pt (IV) complexes have shown enhanced cytotoxicity in tumor cells due to mitochondrial damage (Figure 27). A TPP-functionalized cyclo-metalated Ir (III) complex showed potent reactive oxygen species (ROS) generation upon mitochondrial localization, initiating cell death selectively in cancer cells. This enables organelle-level targeting, better lipophilicity (due to hydrophobic aromatics), and increased cytotoxic ROS.^92^

**Figure 27:**
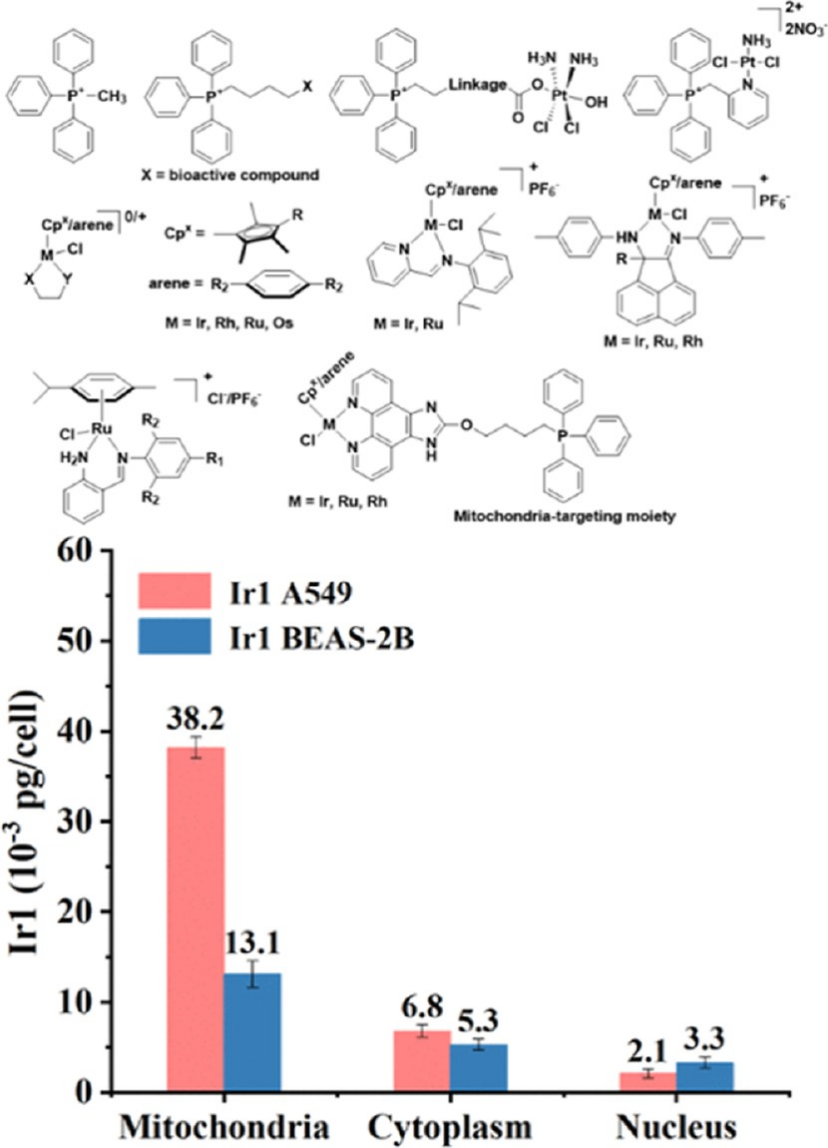
Ir, Ru, and Pt complexes with TPP substituents that target mitochondria. The graph shows iridium accumulation is favored in mitochondria due to TPP substituents **(Liu, Z** et al. **2024).** ^92^ Adapted from ref 93. Copyright 2024 American Chemical Society.

However, due to normal tissues having high mitochondrial activity, this could lead to a lot of systemic toxicity and damage to normal cells. This method is organelle selective but is not truly tumor selective unless used in combination with other strategies.

Recent strategies use cleavable TPP linkers that only release the targeting moiety upon tumor-specific triggers like GSH or acidic pH.^93^ Others use dual-function methods where mitochondrial accumulation is paired with light-triggered activation (a method that will be covered later in the external stimuli section).

### 5.2 Lysosomal Targeting

Lysosomes are acidic organelles (pH ∼4.5–5.5) involved in digestion and recycling. Many cancer cells have increased lysosomal activity and volume.^94^ Metal complexes can be designed to accumulate in lysosomes either through protonation of basic functional groups or via conjugation to targeting moieties that favor accumulation in lysosomes. This enables organelle-specific drug activation, triggering lysosomal membrane permeabilization and cell death.^95^

Phosphine-imine half-sandwich iridium­(III) complexes preferentially localize in lysosomes and generate ROS upon light activation, damaging the lysosomal membrane and inducing cell death.^96^ Platinum and cyclo-metalated iridium complexes with weakly basic ligands have high cytotoxicity when accumulated in the lysosomal environment (Figure 28).^97^

**Figure 28:**
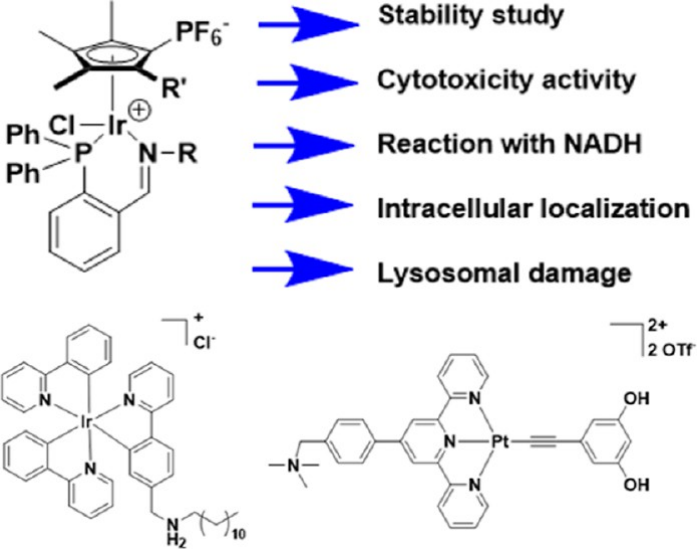
Platinum and iridium complexes with lysosomal targeting. Red dye-stained lysosomes slowly leaking out from (a) to (b) showing selective lysosomal damage caused by the imine half sandwich iridium complex **(Yang** et al. **2019)** ^96^ (Qiu et al., 2019).^97^ Adapted/redrawn using Chemdraw software based on the literature from refs 96 and 97 .

The key advantage includes exploitation of low pH and increased activity of lysosomes in tumors. However, excessive lysosomal membrane permeabilization may cause extreme levels of insufficient detoxification and recycling of waste to an extent where too much waste remains for normal cell lysosomes to handle, leading to normal cell inflammation.^98^ Some lysosomal targeting ligands may be nonspecific and accumulate in other acidic compartments such as the Golgi apparatus. The Golgi apparatus is an organelle that can contain small acidic areas, especially in the parts where proteins are processed and sorted. These slightly acidic regions may unintentionally attract lysosome-targeting complexes, leading to a weaker cytotoxic effect.^99^

### 5.3 DNA Binding

DNA in cancer cells often forms secondary structures such as G-quadruplexes, i-motifs, and three-way junctions, especially in regions of oncogenes or at telomeres.^100^ Metal complexes can be designed to selectively bind to these structures over normal B-form DNA, interfering with replication, transcription, or telomere maintenance and promoting selective cancer cell death.^101^

Platinum­(II) and ruthenium­(II) complexes have been designed to intercalate specifically to G-quadruplex DNA, inhibiting the expression of genes like c-MYC or VEGF. Some complexes include planar aromatic ligands (dipyrido-phenazine) that stack selectively on G-quartet planes, increasing affinity and specificity (Figure 29).^102^ Iridium­(III) complexes have also demonstrated selective binding to telomeric G-quadruplexes, disrupting telomerase activity and inducing sequenced cell death.^103^

**Figure 29:**
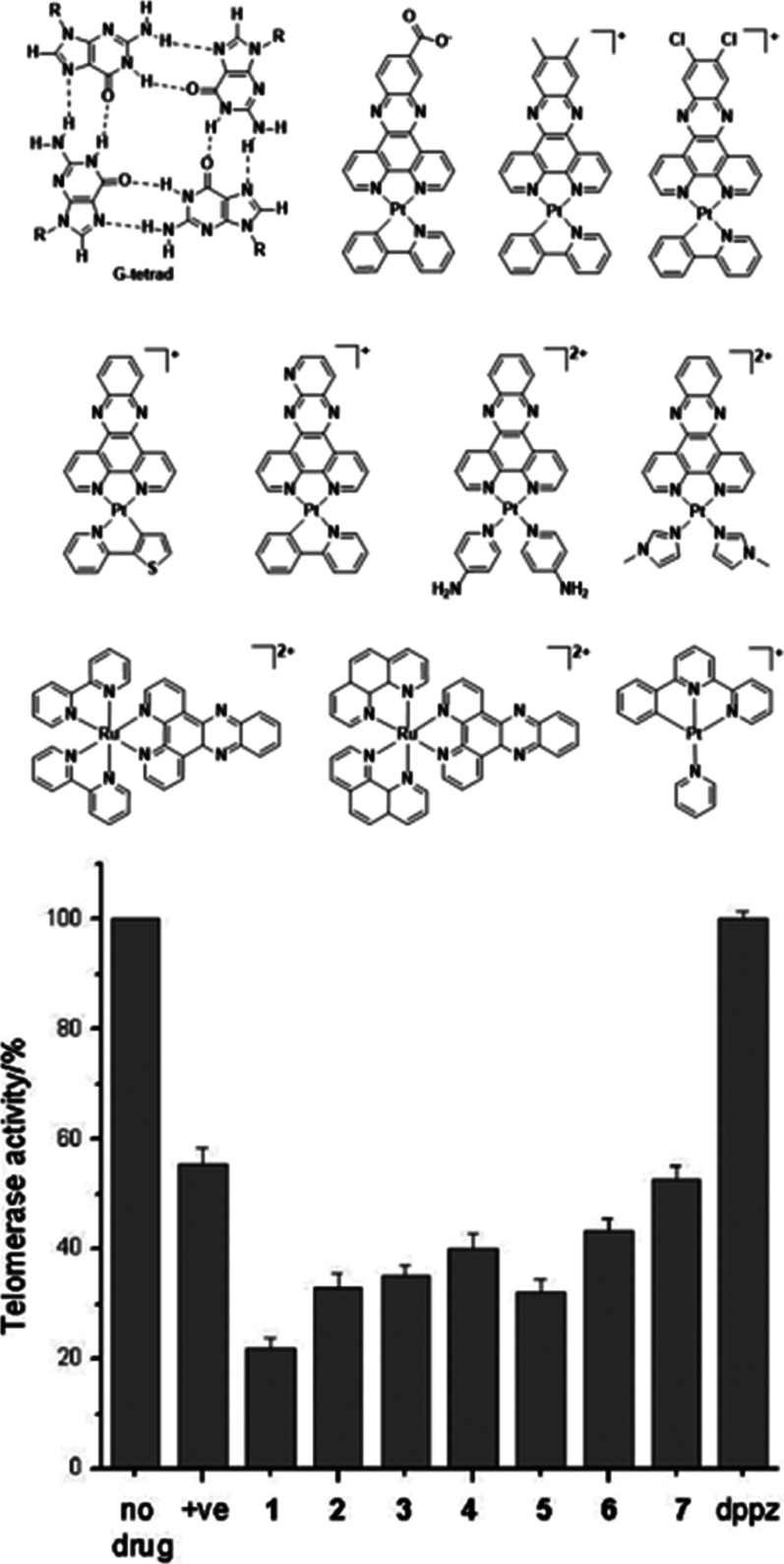
Platinum and ruthenium complexes causing significant inhibition of telomerase activity by binding to G-quadruplexes such as G-tetrad **(Ma** et al.**, 2009).** ^102^ Adapted from ref 103. Copyright 2009 American Chemical Society.

These metal complexes target DNA-related structures unique to cancer biology that are not present in normal gene regulation and can combat common resistance mechanisms related to DNA repair enzymes. However, these secondary structures of DNA are present for very brief periods of time, making them hard to target to a significantly cytotoxic level. Rapidly dividing cells in healthy bone marrow and gut lining could be accidentally targeted by the metal complexes.^104^

Recent research has focused on structure-specific imaging agents, theranostic probes, and complexes that disrupt G-quadruplex-binding proteins in cancer cells. A new generation of luminescent Ir­(III) G4-targeting agents is being developed for real-time imaging and gene-specific damage.^105^

### 5.4 Nuclear Delivery Systems

For many DNA-targeting metal complexes to have cytotoxic effects through intercalation, binding, and inhibition of replication, they must first be able to accumulate in the nucleus. However, the nucleus has very selective transport mechanisms that act as barriers of entry. Nuclear delivery systems use nuclear localization signals (NLS), DNA-binding functional groups, or nanocarrier-based delivery to aid in active transport or passive diffusion of the complex into the nucleus.^106^

Platinum­(II) complexes (Figure 30) with peptide-based NLS tags (sequences derived from SV40 T-antigen such as PKKKRKV) exhibit enhanced accumulation in the nucleus and DNA binding.^107^ Metal complexes and other anticancer drugs have been shown to reach the nucleus when attached to certain carrier peptides or when encapsulated in nuclear-penetrating nanoparticles.^108^

**Figure 30:**
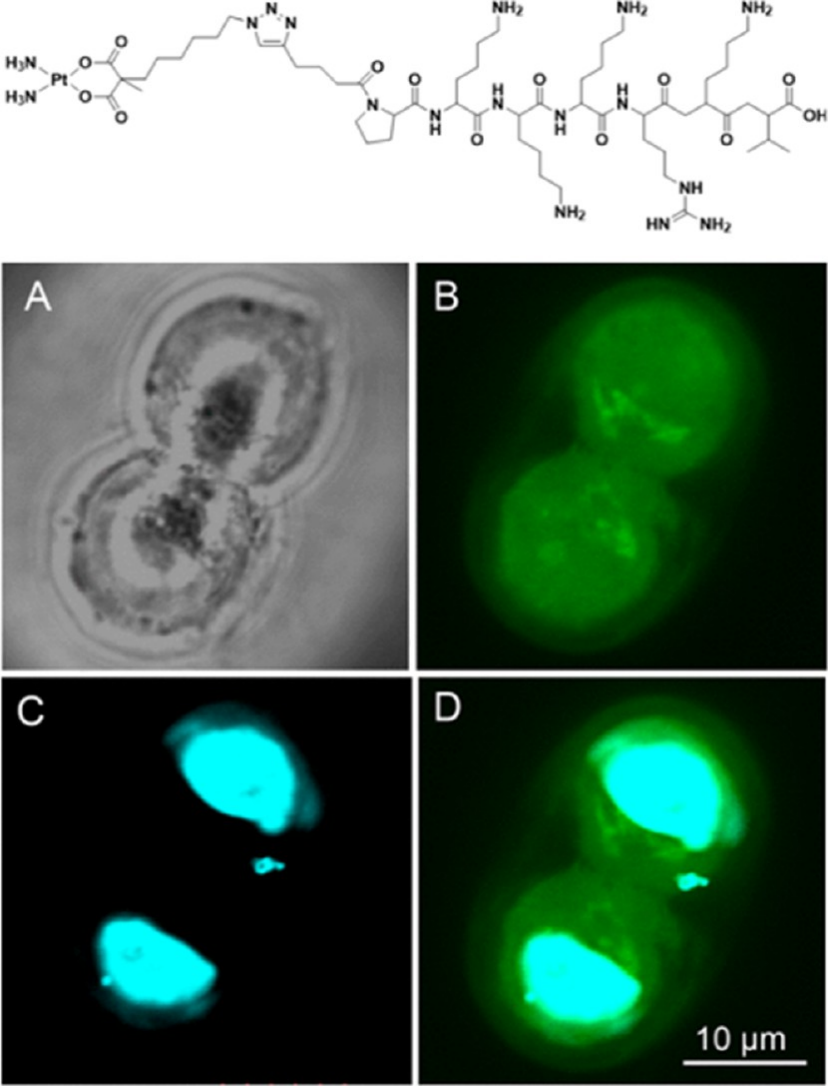
PKKKRKV peptide linked to a platinum complex for nuclear delivery. Localization in nuclei indicated in (D) **(Wlodarczyk** et al. **2018).** ^107^ Adapted from ref 108. Copyright 2018 American Chemical Society.

This strategy is very nucleus-specific and can bypass activation of the drug while passing other organelles and resist other triggered cytotoxicity. Despite this, large peptides like PKKKRKV can be unstable in circulation, hard to synthesize, and could target nearby healthy cell nuclei.^109^ These peptides would be better utilized in conjunction with nanoparticle delivery systems (discussed in the Delivery Systems section).

## 6 External Stimulus-Controlled

### 6.1 Photodynamic Therapy (PDT)

Photodynamic therapy involves the use of photosensitive compounds that, upon activation by light of a specific wavelength, generate reactive oxygen species, particularly singlet oxygen (^1^O_2_), which damages nearby mitochondria and causes cell death (Figure 31).^111^ Metal complexes are ideal photosensitive compounds due to their strong photophysical properties (fluorescence and phosphorescence) (Figure 32), including long-lived excited states and tunable redox behavior. Selectivity is achieved by exposing only the tumor area to light.^112^

**Figure 31:**
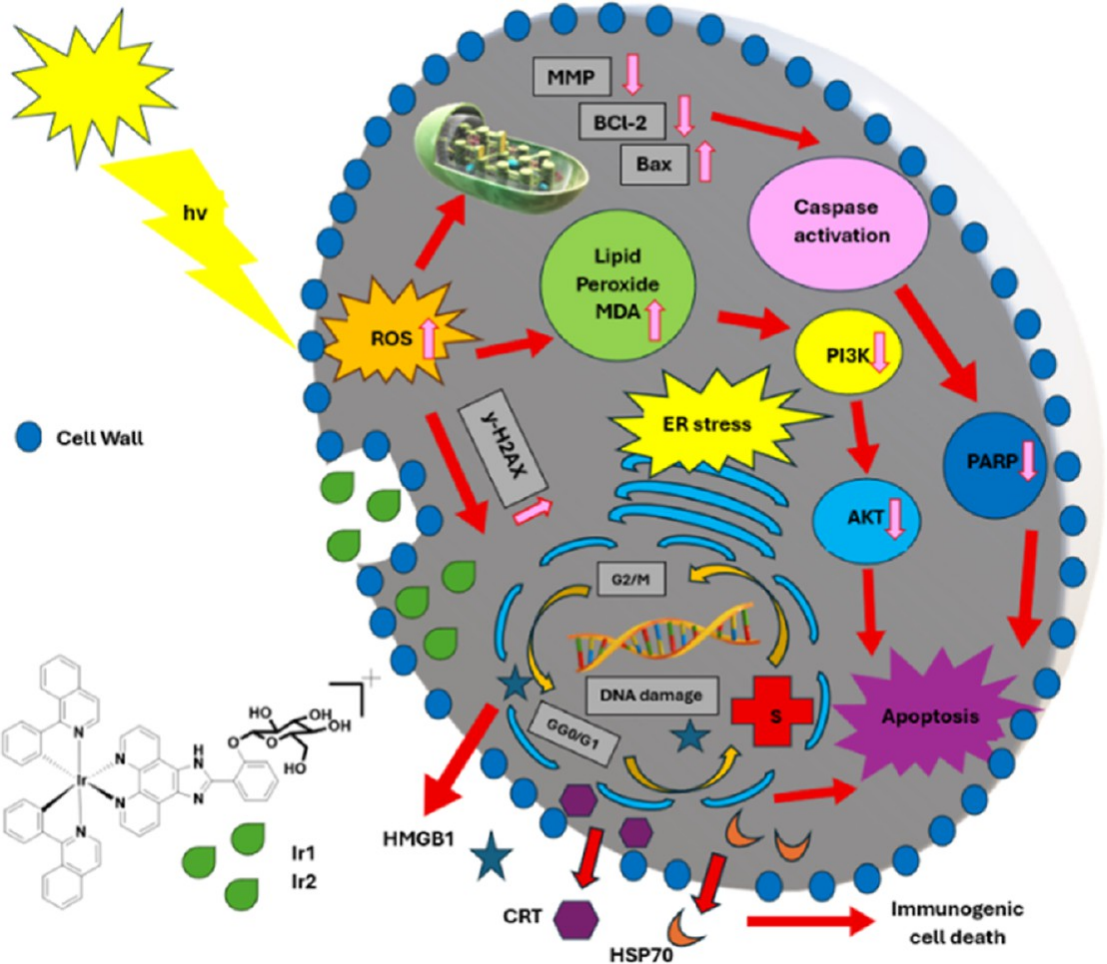
PDT using light-activated iridium complexes to produce ROS resulting in apoptosis **(Li** et al.**, 2022).** ^110^ Adapted/redrawn using Chemdraw and Microsoft Word based on the literature from ref [Bibr ref111] 2022 Elsevier.

**Figure 32:**
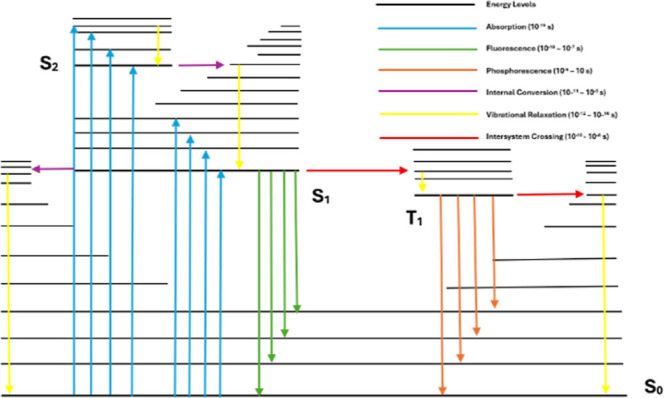
The excitation of electrons in metal complexes from ground state (S_o_) to singlet (S_1,2,3_) and triplet states (T_1_), causing either fluorescence or phosphorescence **(Edinburgh Instruments, 2023).** ^113^ Redrawn/adapted using Microsoft Word based on the literature from ref [Bibr ref114] 2023 Edinburgh Instruments.

Iridium­(III) and ruthenium­(II) polypyridyl complexes are the most advanced PDT complexes. These can be fine-tuned to absorb visible or near-infrared (NIR) light and produce ROS efficiently (Figure 33).^114^ They can be tuned to be blue-shifted to kill bacteria/cancer with light emission; however, this can be damaging to nearby tissue, and the blue-light absorption will not be effective for deep tissue tumors in comparison to red-light absorption and emission. This is due to hemoglobin absorbing blue light. Therefore, red-shifted complexes are usually used for tumor imaging.^115^

**Figure 33:**
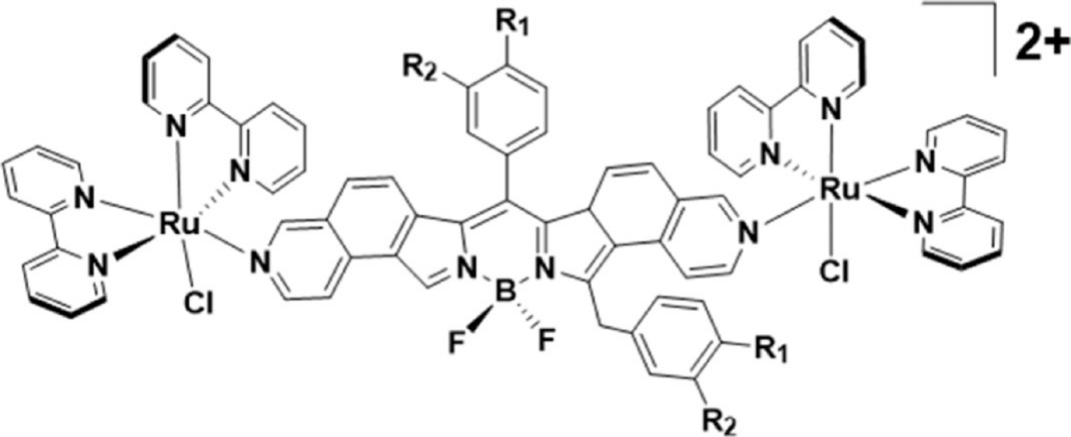
Polypyridyl Ru (II) complex with tunable photophysical properties via change in R_1_ and R_2_ **(Swavey** et al.**, 2017).** ^114^ Adapted from ref 115. Copyright 2017 American Chemical Society.

This allows excellent control of the location the drug is activated and offers an alternative to resistant tumors. Recent research favors NIR-shifted complexes for deep and less systemically toxic imaging.

### 6.2 Magnetically Guided Nanoparticles

Magnetically guided delivery uses external magnetic fields to direct metal nanoparticles toward tumor sites. This is typically achieved by using superparamagnetic iron oxide nanoparticles (SPIONs), which can be functionalized with therapeutic metal complexes and other anticancer drugs. By applying a magnet near the tumor site, the particles are pulled toward and retained in the desired location, enhancing tumor accumulation and reducing systemic exposure.^116^

SPIONs functionalized with anticancer platinum drugs (Figure 34) have demonstrated increased tumor delivery and reduced systemic toxicity compared to regular platinum drugs.^117^ Some ferrocene-based metal complexes exhibit magnetic properties and can be guided without additional nanocarriers.^118^

**Figure 34:**
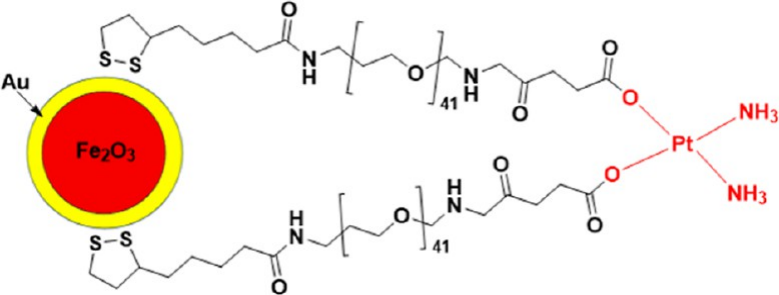
Gold plated iron oxide nanoparticle with an anticancer platinum complex tethered via the PEG spacer **(Wagstaff** et al.**, 2012).** ^117^ Adapted/redrawn using Chemdraw software based on the literature from ref [Bibr ref118] 2012 Elsevier.

Overall, this allows spatial control of where the metal complex is accumulated in the body, increasing the likelihood of a selective effect. Limitations include precise control of field strength and localization and may not be effective if the tumor is deep tissue. The SPION must be both stable in circulation but also biodegradable, otherwise it risks long-term accumulation in the liver or spleen.^119^

New generations of biodegradable magnetic nanocarriers are being developed to avoid long-term tissue retention. Magnetic fields are also being used with remote-controlled release, where heating or oscillation triggers drug release at the tumor site. Magnetically guided Fe (III)–salen complexes and Pt (IV)–SPION hybrids can also be used as selective cancer therapies.^120^

### 6.3 Photo Thermal Therapy (PTT)

PTT uses photosensitive compounds that, upon irradiation (usually with NIR), convert light energy into localized heat, causing thermal destruction of cancer cells.^121^ Metal nanoparticles are excellent examples (due to light interacting with a metal making electrons oscillate along its surface generating energy), photostability, and tunable signals.^122^ Selectivity is achieved through localized light exposure and/or targeted delivery of the metal complexes inside nanocarriers.

Gold nanoparticles and gold nanorods are PTT agents that absorb NIR light and generate heat (see Figure 35). When conjugated with targeting ligands and metal complexes drugs, they enable dual targeting therapy.^124^ Copper sulfide nanoparticles, sometimes with ruthenium or other metal complexes, have been used for simultaneous imaging and PTT.^125–127^

**Figure 35:**
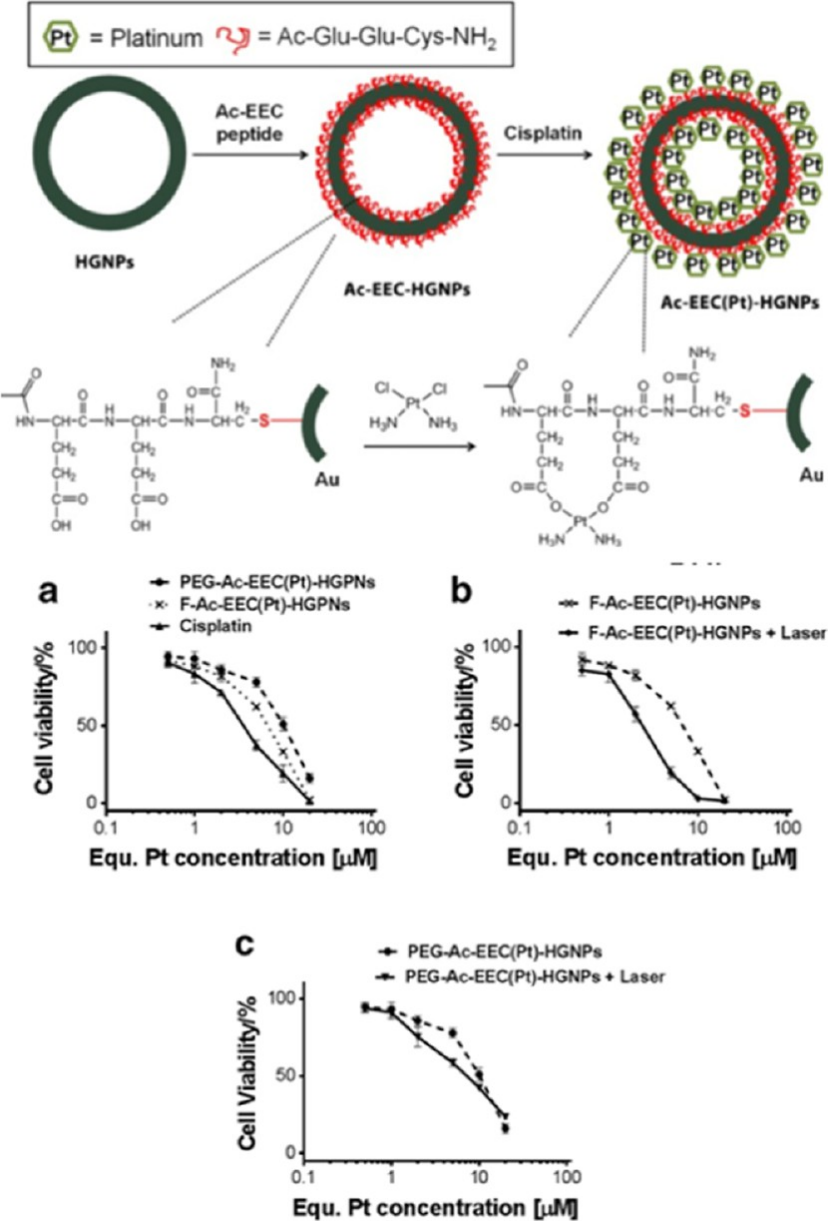
Hollow gold nanoparticles containing cisplatin caused more cytotoxicity toward cancer cells than cisplatin alone. Nanoparticles utilizing laser activation/release of cisplatin via photothermal effect is even more efficient. PEG lowers cytotoxicity **(Xiong** et al.**, 2018).** ^123^ Adapted from ref 124. Copyright 2018 Springer Nature Link. Creative Commons Attribution 4.0 International.

This provides noninvasive (as it is externally stimulated) and localized treatment with minimal systemic toxicity. This can overcome drug resistance, particularly in tumors not sensitive to traditional chemotherapy. Metal complexes in PTT-activated nanoparticles often double as imaging agents (CT/MRI scans).

Limitations are similar to those of PDT. PTT depends on light penetration, which may be limited to deep tissues. Highly localized heat may damage nearby normal cells if not precisely controlled.^128^ Some inorganic nanomaterials like gold are not biodegradable, raising concerns about long-term retention and usage of the method.^129^

Recent designs use biodegradable metal nanocarriers, such as gold-coated silica, metallic alloys, or Cu-based hollow spheres, optimized for tumor-specific accumulation and rapid clearance.^130^

## 7 Delivery Systems

### 7.1 Liposomes

Liposomes are spherical vesicles made of one or more lipid bilayers surrounding an aqueous core. They can encapsulate both hydrophilic and hydrophobic metal complexes, protecting them from degradation and reducing off-target toxicity.^131^ Selectivity comes from prolonged circulation, enhanced tumor accumulation via the EPR effect, and the ability to incorporate targeting ligands or pH-sensitive release mechanisms.^132^

Lipoplatin, a liposomal cisplatin, reduces kidney toxicity and improves tumor selectivity.^133^ Liposomes encapsulating ruthenium­(II) complexes have shown improved pharmacokinetics, controlled release, and better tumor uptake.^134^ Some methods use pH- or redox-sensitive nanoniosomes that release metal complexes only under tumor-specific conditions.^135^

The benefits of this strategy include increased stability and solubility of metal complexes. Liposomes enable controlled and steady release and can be functionalized with antibodies, peptides, and other targeting groups for active targeting reducing systemic toxicity.^136^

Unfortunately, encapsulation efficiency can be low for certain metal complexes, and liposomes may be rapidly cleared by the system without PEGylation. There is also risk of drug leakage during storage or circulation.^137^

Modern liposomal strategies use multistimulus responsiveness (pH and redox), triggered release (light and enzymes), or delivery of imaging agents for diagnosis. New stealth liposomes with PEGylation and charge-modified surfaces improve circulation and tumor accumulation (Figure 36).^138^

**Figure 36:**
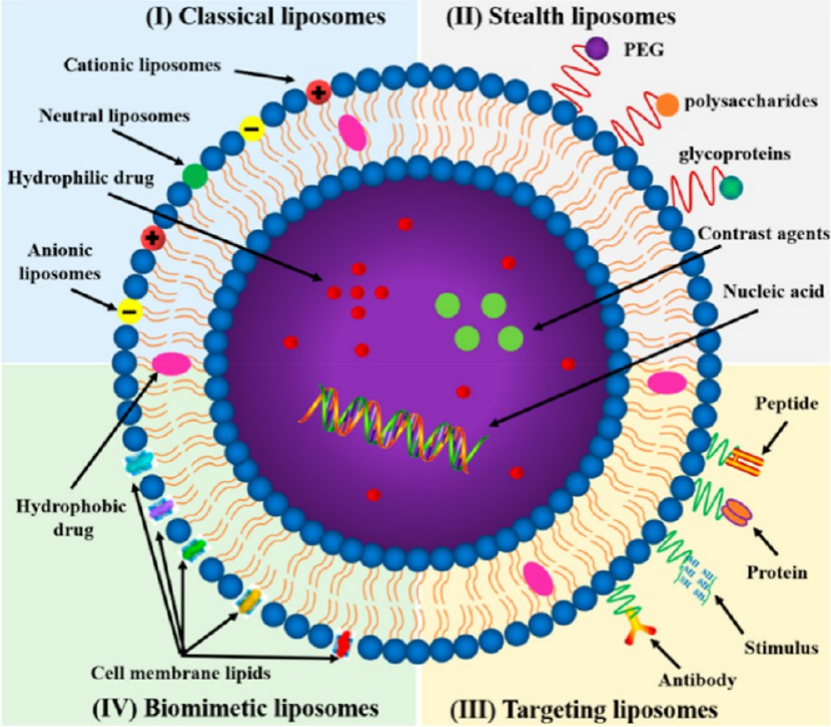
The modifications that can be made to liposome surfaces, improving selectivity **(Li** et al.**, 2020).** ^138^ Reproduced from ref 139. Copyright 2020 American Chemical Society.

### 7.2 Dendrimers

Dendrimers are branched, treelike macromolecules with a central core that has repeating units and a lot of surface functional groups (Figure 37). Their precise structures allow for the conjugation or encapsulation of metal complexes and the attachment of targeting ligands, imaging agents, or probing moieties. Selectivity is achieved through multifunctional, passive accumulation, and controlled release inside the cell.^139^

**Figure 37:**
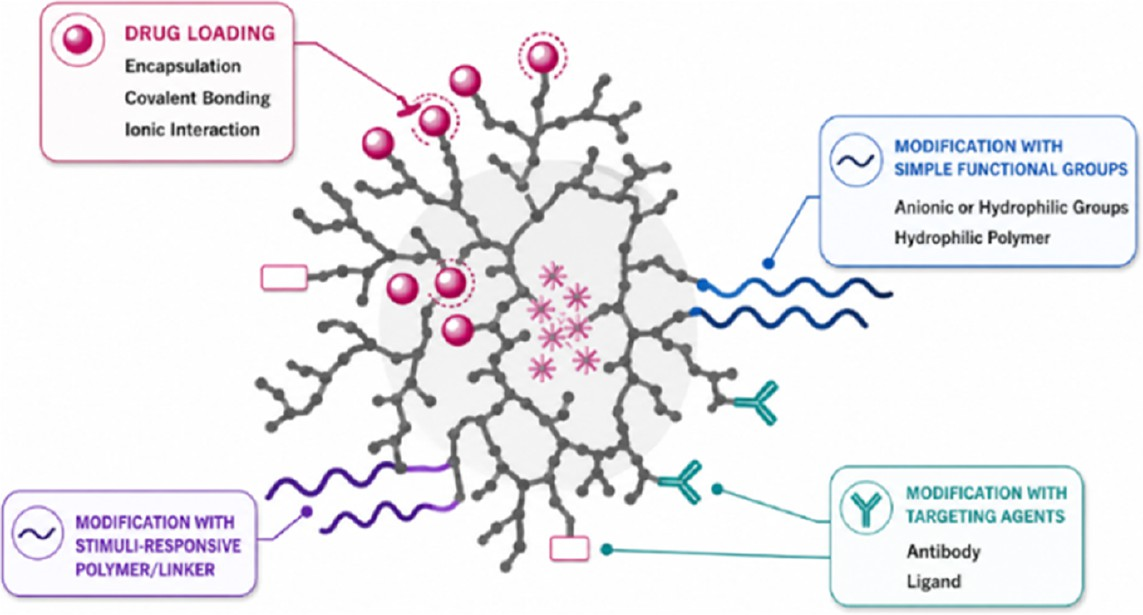
A diagram showing the branched structure of a dendrimer that can accompany anticancer metal complexes and targeting groups **(Dehkordi** et al. **2025).** ^143^ Redrawn using Microsoft Word shapes based on literature from ref 144.

Platinum­(IV) complexes have been conjugated to polyamidoamine (PAMAM) dendrimers, enhancing tumor uptake and reducing kidney toxicity.^140^ Copper­(II) complexes have been attached to dendrimers to improve solubility and enable targeted therapy.^141^ Dendrimers with folate or RGD peptides have shown selective delivery to cancer cells via receptor targeting uptake.^142^

These types of nanoparticles have a higher loading capacity and vacancies for the addition of many targeting moieties to the surface. However, PAMAM can be toxic if not modified properly. The benefits of this delivery system come at the cost of complicated synthesis and low scalability.^143^

Recent studies have developed biodegradable dendrimers with cleavable internal linkages, stimulus-sensitive conjugates, and dual-function carriers that combine imaging and therapy. Some dendrimers use enzyme-responsive or redox-cleavable bonds to release metal complexes selectively in cancer cells. Others use magnetic or photothermal responsive compounds for guided therapy.^144^

### 7.3 Metal–Organic Frameworks (MOFs)

Metal–organic frameworks (MOFs) are highly porous crystalline materials made of metal nodes (Zr, Fe, Zn) coordinated to organic linkers. Their tunable pore size, high surface area, and structural design make them ideal carriers for metal complexes. MOFs can encapsulate, coordinate, or release metal complexes in a controlled or stimulus-responsive way, improving selectivity through passive targeting or functional surface modification.^145,146^

Zr-based MOFs (UiO-66) can be used to encapsulate cisplatin, releasing it in response to acidic pH within the tumor environment.^147^ Porphyrin-linked MOFs act as photosensitive compounds in PDT, with potential for embedded Ru­(II) or Ir­(III) centers (Figure 38) providing photoreactivity.^148^ Iron-based MOFs have been designed to deliver Pt­(IV) complexes and GSH-sensitive ligands, increasing production of ROS and causing cell death selectively in cancer.^150^

**Figure 38:**
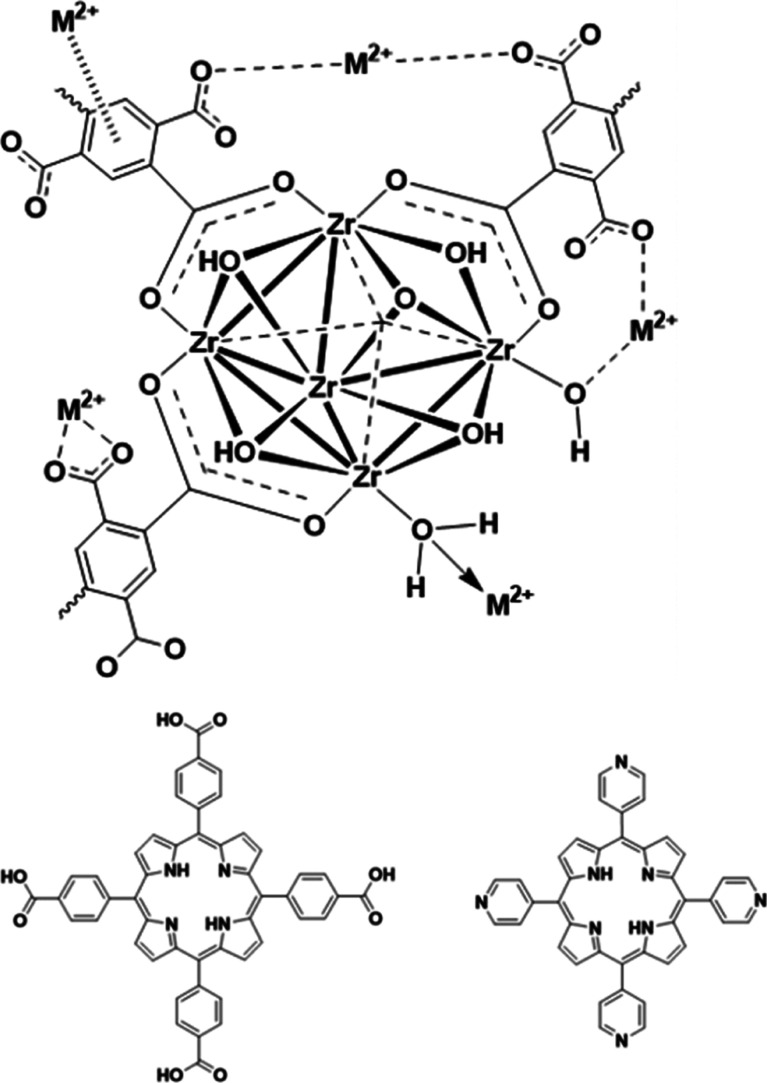
Zr-based MOF (Ui O -66) that can be used to hold M^2+^ metals such as cisplatin. Below the MOF are two porphyrins that can be linked to MOFs that can act as photosensitive compounds in PDT, with potential for embedded Ru (II) or Ir (III) centers **(Jrad** et al. **2022)** ^147^ **(Sajjadinezhad** et al. **2024).** ^148^ Adapted from ref 148. Copyright 2022 American Chemical Society. Redrawn using Chemdraw based on ref 149.

This method has the same benefits as dendrimers, except it has added tunability and biodegradability. Once again, the scalability and synthesis of these multifunctional structures are difficult and not very scalable.

## 8 Critical Analysis of Selectivity Strategies

So far, this review has summarized seven types of methods that have and or can be used to make metal complexes selectively target cancers. The key underlying principles are explained in the sections above.

This section below aims to offer a detailed critique of each method and evaluate each method’s efficacy when used in isolation. This judgment has been made based off biocompatibility, biodegradability, dosage required, scalability, and the overall evaluation of how selective the strategy is toward cancer cells in clinical trials.

The table below (Table 1) states the advantages and disadvantages of each method while showing additional examples of metal complexes used in each cancer-selective strategy.

**Table 1:**
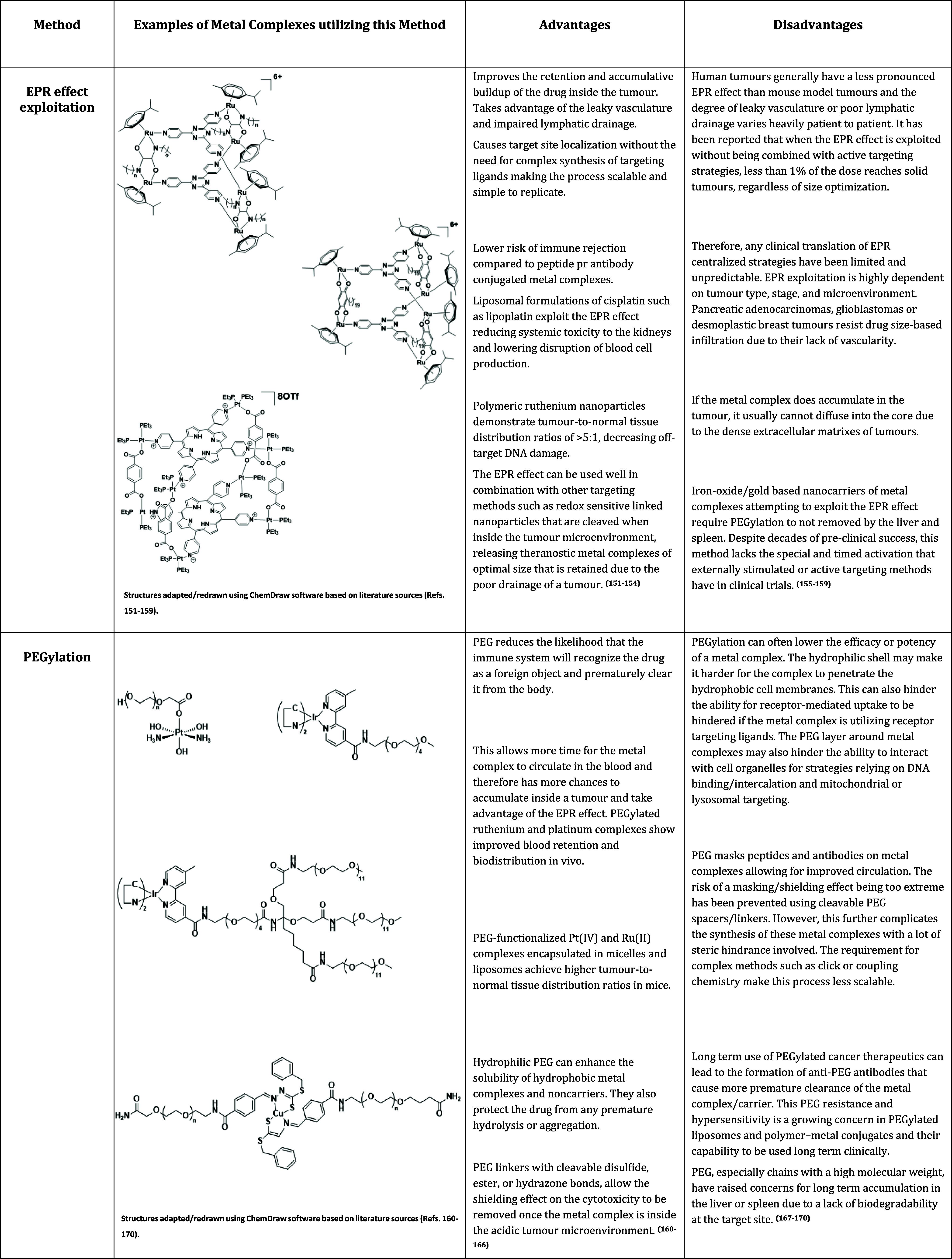

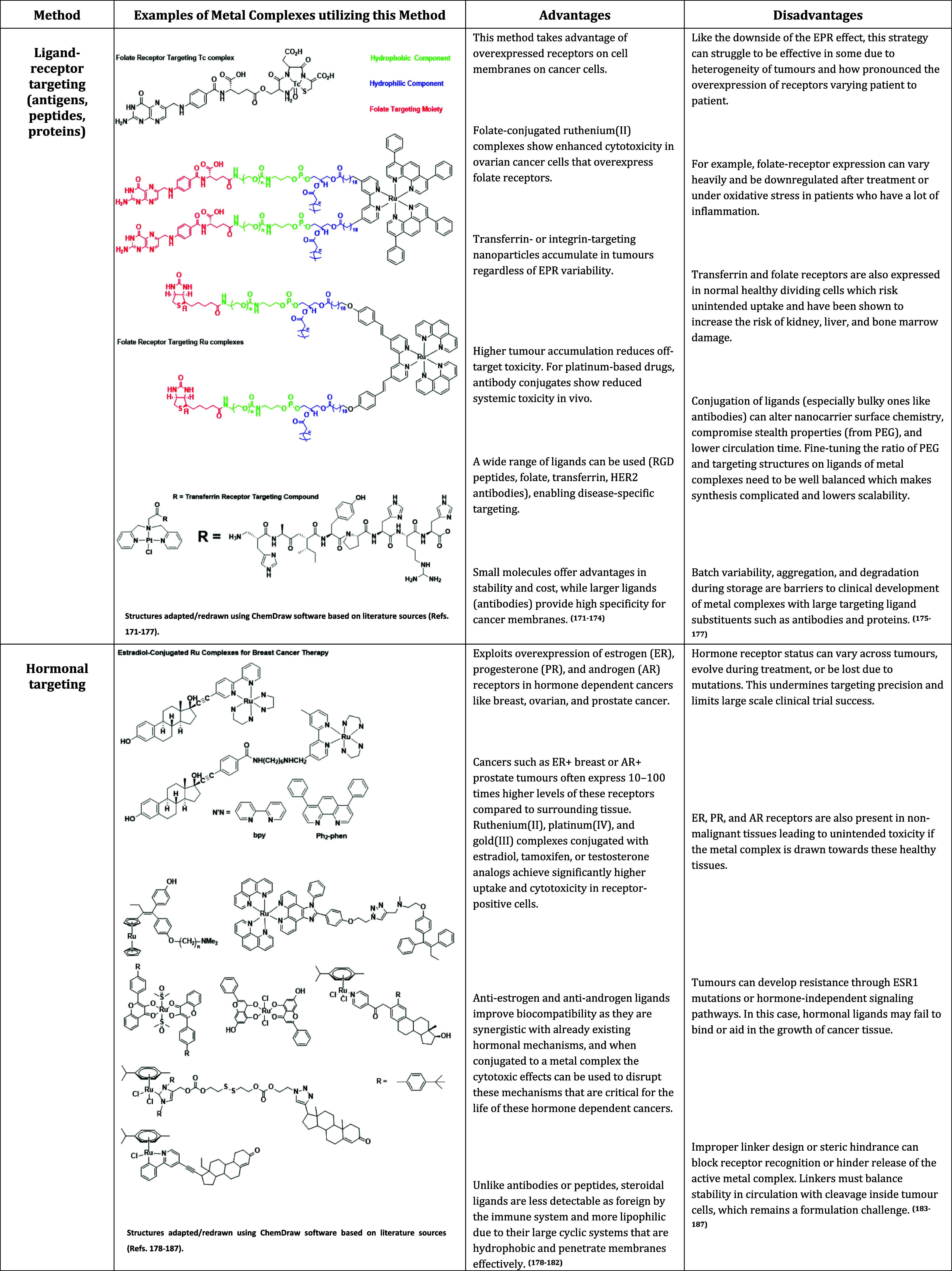

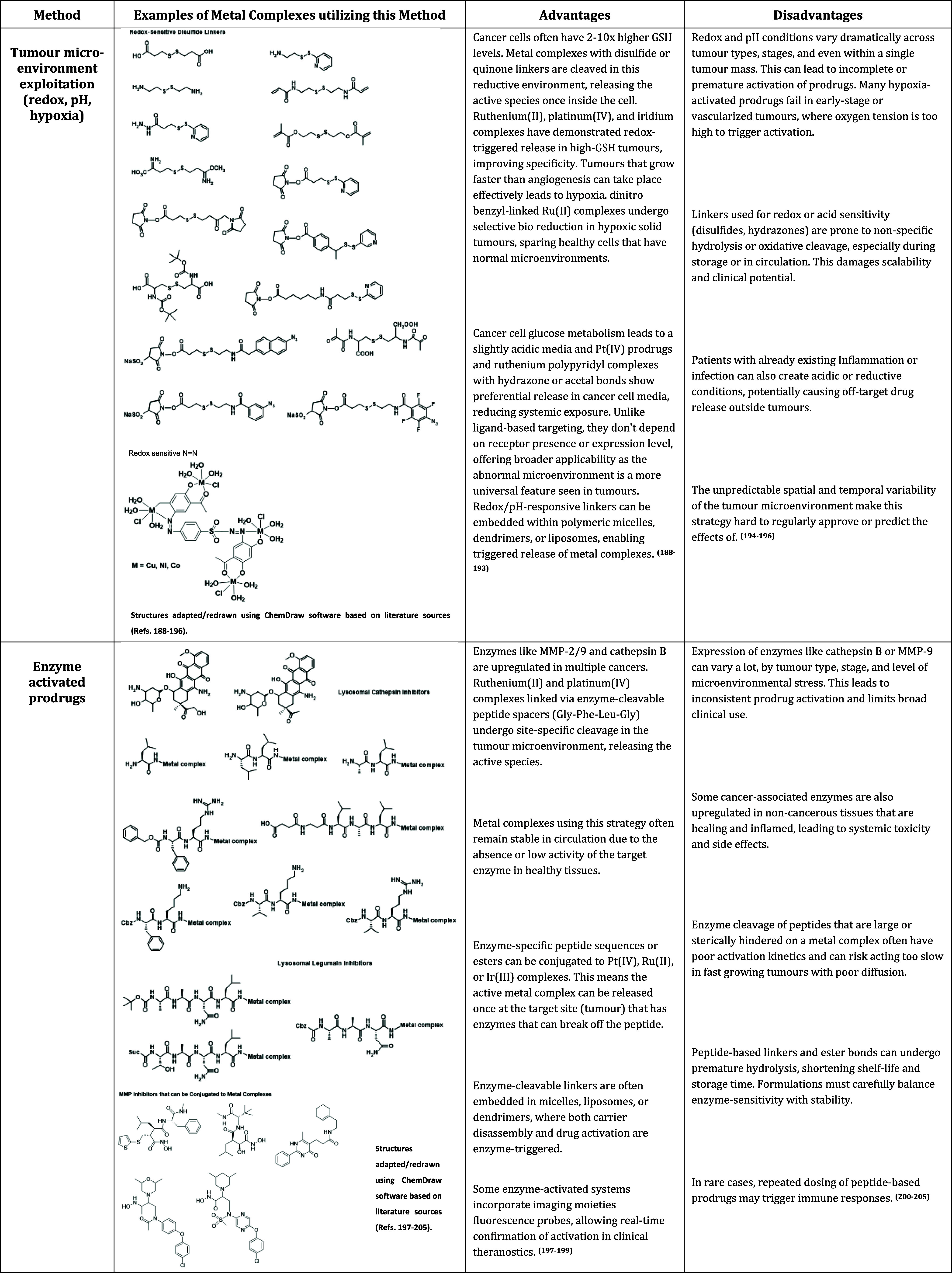

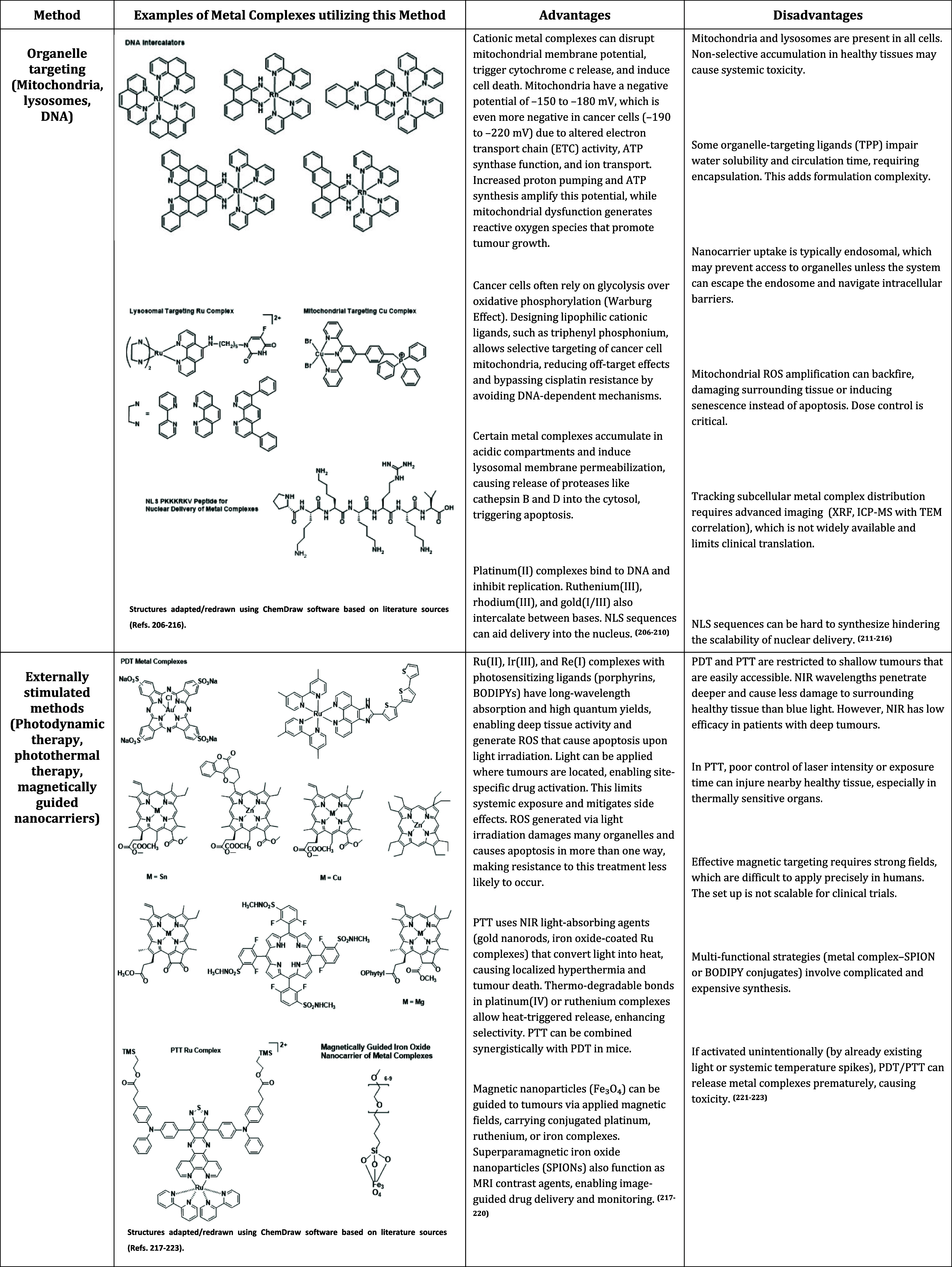

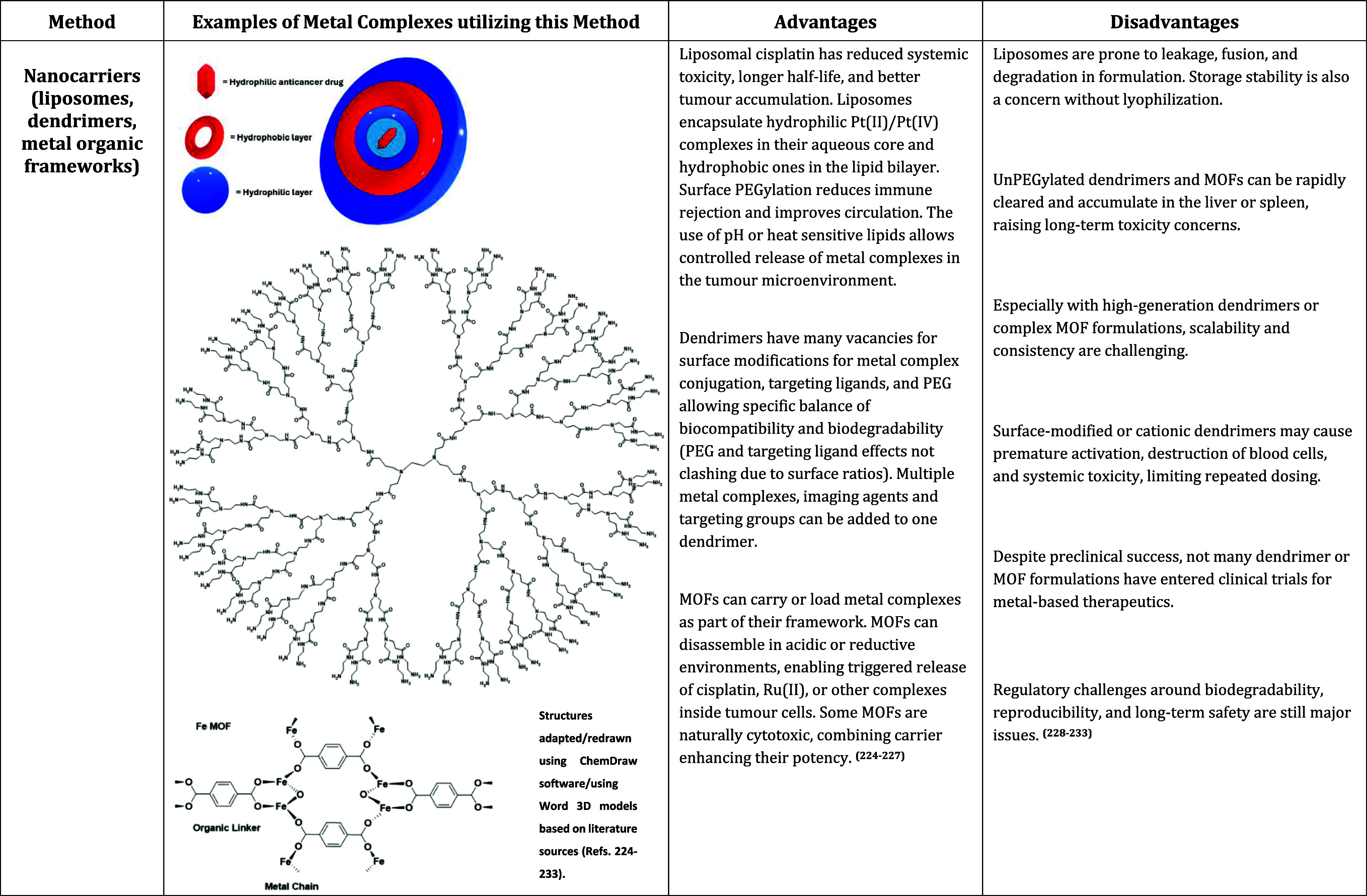
Additional Examples, Advantages, and Disadvantages of Each Selectivity Method Discussed^224–181^ ^,^ ^182–212^ ^,^ ^213–223^

## 9 Most Common Combinations of Targeting Strategies

### 9.1 Introduction

Many of the targeting strategies discussed in this Review have successfully improved tumor selectivity. However, recent studies have demonstrated that this is often accompanied by strategy-specific limitations. Enhancing the performance in one area frequently causes drawbacks in others. For example, PEGylation can improve circulation time and improve stealth properties of drug delivery, but this is often associated with reduced cytotoxic potency compared with non-PEGylated analogues. This section presents selected case studies that have progressed to advanced preclinical or clinical evaluation, showing how the modern design of metal-based anticancer agents increasingly relies on the combination of complementary targeting strategies. The examples discussed in this section highlight how delivery vehicles, active targeting approaches, and physicochemical design principles can operate simultaneously to enhance selectivity while retaining anticancer efficacy and limiting systemic toxicity, allowing for clinical success.

### 9.2 Mitochondrial Targeting with PDT

As seen in Figure 39, mitochondrial targeting and PDT are a way that strategies can be combined to make a dual-function anticancer drug. These two strategies work synergistically to induce cell death via many pathways overcoming any patient resistance to traditional chemotherapy. The amide linkage and spacing of the triphenylphosphonium group ensure that there is no steric interference between the targeting group and the ROS generator. The lipophilic, hydrophobic, and cationic nature of the mitochondrial targeting group boost the drug’s affinity for cancer cell membranes as well as the attraction toward the enhanced negative inner membrane potential of cancer cell mitochondria. The ROS produced not only damage mitochondria but also other cell organelles causing apoptosis. Hydrogen bonding or Pi–Pi stacking with DNA is also possible due to the aromatic and amine groups. This strategy proved to be largely nontoxic to healthy liver cells before or after light irradiation. The drug exhibited an efficient cytotoxicity against cervical cancer in vivo. Cytotoxic activity was confined to the areas that were illuminated, leading to less systemic damage. The synthesis of a small molecule containing two strategies simplifies replicability compared with systems that use large antibodies as targeting groups.

**Figure 39:**
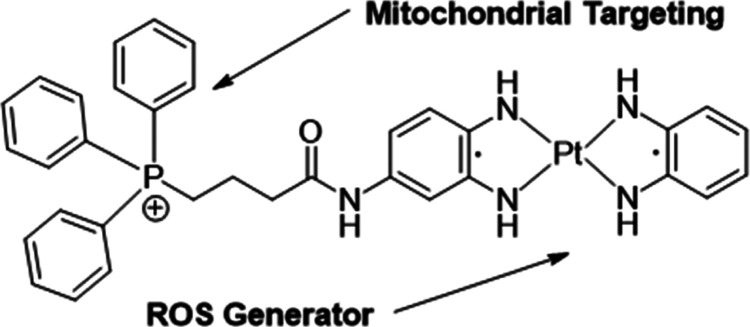
Platinum-diamine complex with an amide-linked mitochondrion targeting a triphenyl phosphonium group and ROS generator via PDT **(Qi** et al.**, 2024)**.^231^ Structure redrawn/adapted using ChemDraw software based on ref 233.

Cytotoxicity is less efficient for targeting deep tissue tumors. Even when NIR light is used for activation, the singlet oxygen generation is moderate and may restrict potency. The radical generator and cationic targeting group are difficult to remain stable while being stored. While this method is successful in vivo, it has not yet been proved to be a contender with traditional chemotherapy in human clinical trials. PEG is difficult to add to this compound to improve stealth and circulation without hindering the photosensitivity or mitochondrial targeting. To improve the overall clinical potential, a drug like this would need to be combined with functionalized nanocarriers.^222^

### 9.3 Tumor Microenvironment-Sensitive Magnetically Guided Nanocarrier of the Anticancer Pt­(IV) Complex

Pt­(IV) prodrug dihydroxy cisplatin was tethered to the surface of a super paramagnetic iron oxide nanocarrier that had a polyacrylic acidic coating (Figure 40). The magnetic properties of the nanocarrier allowed spatial control of the therapeutic drug. The carboxylic acid coating allowed the Pt­(IV) complex to be conjugated to the carrier’s surface via amide and or ester bonds. The acidic layer offers biocompatibility and pH responsiveness allowing the Pt­(IV) drug to be cleaved from the nanocarrier once inside the tumor microenvironment. The coating allows the magnetic core to be preserved for targeting and MRI tracking. The work demonstrates high tumor accumulation in vivo, combined with low systemic toxicity, aligning well with the magnetically guided and passive targeting hybrid strategies.

**Figure 40:**
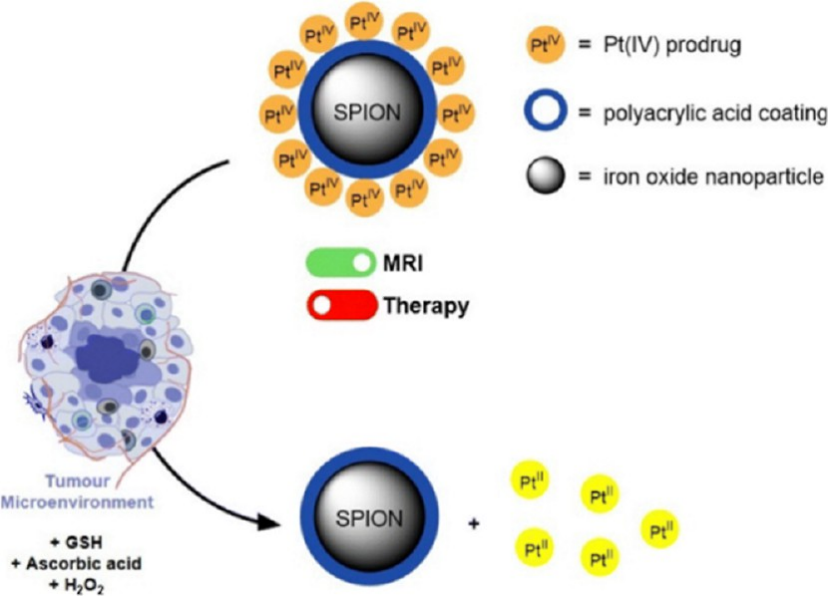
Magnetically guided SPION with biocompatible acidic coating that releases Pt­(IV) prodrug inside the tumor microenvironment **(Brito** et al.**, 2025)**.^232^ Reproduced with permission from ref 234. Copyright 2025 Royal Society of Chemistry.

However, Pt­(IV) prodrug linkage may hydrolyze before reaching the tumor, the nanocarrier can be cleared and accumulate in the liver and spleen leading to long-term toxicity, poly­(acrylic acid) may trigger immune system response or interfere with protein absorption, and the complexity of this strategy hinders scalability.^233^

### 9.4 PSMA-Targeting PEGylated Nanoparticle Delivery

This method utilized polymeric nanocarriers capable of delivering a combination of the anticancer compounds irinotecan and cisplatin. The nanoparticle was functionalized with PSMA-targeting ligands, allowing selective uptake by the overexpressed antigens on the surface of LNCaP prostate cancer cell lines (Figure 41). Compared to nontargeting control variables, the PSMA-targeting nanoparticles showed an 8x higher uptake in prostate cancer cells with overexpressed PSMA. Nanoparticles containing both irinotecan and cisplatin had IC_50_ values 10.6× lower than cisplatin only nanocarriers and 3.6× lower than irinotecan only nanocarriers. This proved targeting to be effective and the combination of drugs to be synergistic. Cisplatin was conjugated to the nanoparticle via polyacetic acid improving biocompatibility and ensuring the complex is released once inside the tumor microenvironment. Irinotecan (hydrophobic) was encapsulated inside the carrier via a single-step microfluidic synthesis.

**Figure 41:**
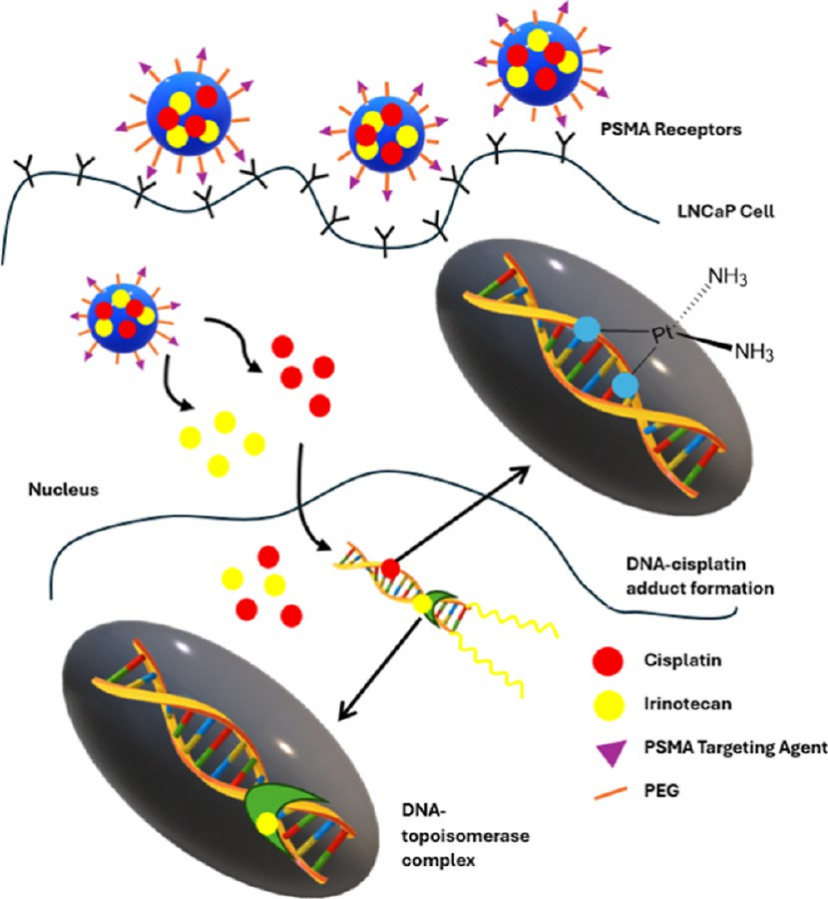
Polymeric nanoparticle using PSMA-targeting peptides to target overexpressed antigens on prostate cancer cell surfaces. The nanoparticle delivers cisplatin to bind to cancer cell DNA and irinotecan to inhibit topoisomerase I (an enzyme responsible for DNA replication) **(Valencia** et al.**, 2012).** ^233^ Adapted/redrawn using Microsoft Word 3D models based on the literature from ref [Bibr ref235] 2012 Taylor and Francis.

This strategy is promising, but it needs to be tested further in vivo to compare the drug combinations with and without nanoparticles to further prove efficacy and validity for human trial testing. The industrial-scale synthesis of dual-drug-ligand-functionalized NPs is still technically challenging and expensive. PSMA expression varies from patient to patient. Irinotecan was trapped, while cisplatin was chemically conjugated. These drugs may release at different rates, lowering the intended synergy.^233^

### 9.5 GSH-Cleavable Polymeric Nanocarriers of Pt­(IV) Prodrugs

This strategy uses a highly biocompatible nanoparticle while also being biodegradable after PEGylation (Figure 42). The biocompatibility comes from the lipid layer of the nanoparticle, almost mimicking the benefits of using a biological nanocarrier, such as a liposome. The PEG improves circulation time, allowing more time and chances for the nanocarrier to localize in the tumor via the EPR effect. The biodegradability comes from the disulfide-linked polymeric layer that accompanied the Pt­(IV) prodrug allowing excess GSH from the tumor microenvironment to release the prodrug from the nanocarrier once inside the tumor. The hydrophobic parts of this design allow prodrug containment and membrane penetration, while the hydrophilic sections add to biocompatibility.

**Figure 42:**
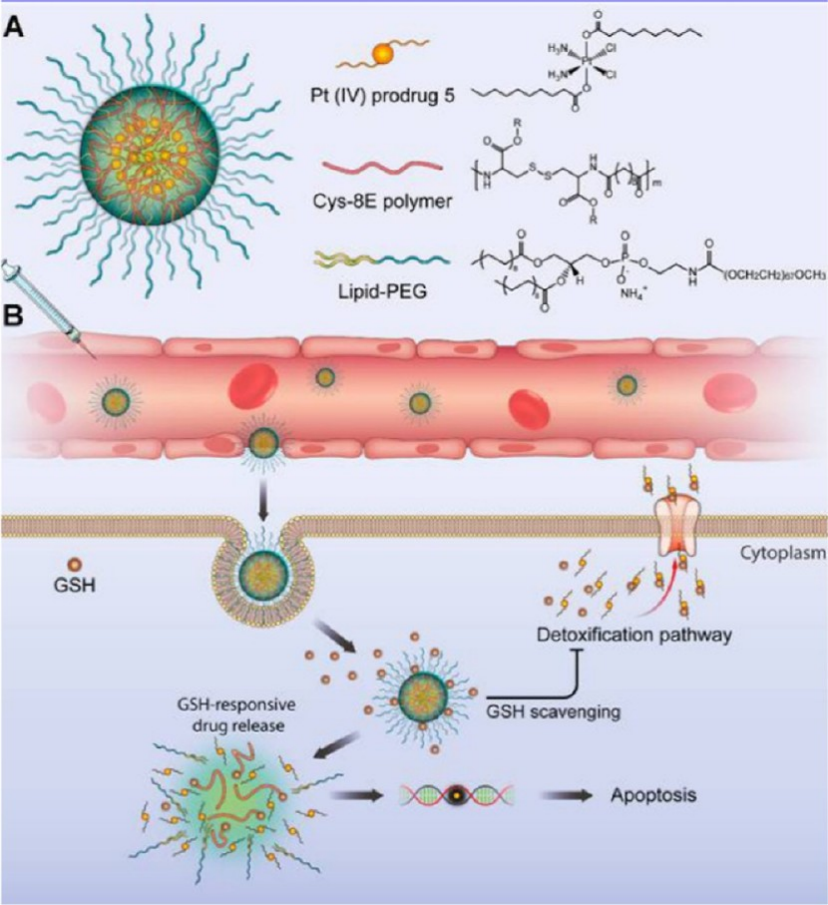
PEGylated lipid-coated polymeric and GSH-responsive nanocarrier of platinum­(IV) prodrug **(Ling** et al.**, 2018).** ^234^ Reproduced from ref 236 under CC BY license.

This is a well-balanced nanoparticle that noticeably reversed cisplatin resistance in A2780cis ovarian cancer cells in vitro. The mouse model studies proved roughly 83% of tumor growth was inhibited with negligible systemic toxicity compared to regular cisplatin. On the basis of the in vitro and in vivo success, this strategy makes a strong case for clinical trial testing.

However, GSH levels vary across cancers and may be insufficient in some tumors to trigger release of the prodrug. Systemic GSH scavenging could damage nontumor tissues or disrupt antioxidant balance. The long-term effects of polymeric disulfide exposure remain unexplored. The complex synthesis of such multifaceted nanoparticles remains a scalable challenge.

### 9.6 Photo-Thermally Triggered Release of Nanocarriers

This study developed gold nanorods (AuNRs) conjugated to cisplatin via mercaptoundecanoic acid linkers, forming Pt-AuNRs that enable external laser-triggered release (Figure 43). NIR laser (850 nm) usage caused cisplatin to be released 15× more effectively than gold nanorods without laser-triggered release. In sarcoma cells, the laser-triggered release reduced IC_50_ values to 11–13× less than cisplatin used without a carrier. Laser activation led to an increase in ROS and led to apoptosis via various organelle damage pathways. Minimal damage to blood cells or macrophages was observed, showing signs of lowered systemic toxicity in comparison to regular cisplatin.

**Figure 43:**
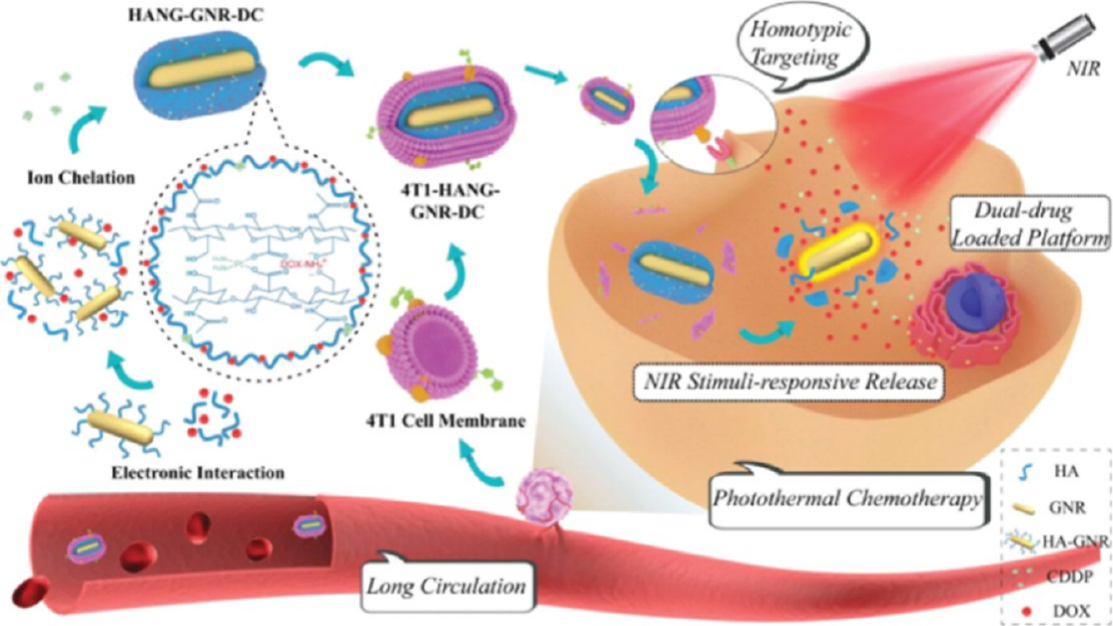
Photothermally triggered release of chemotherapy from a biomimetic nanocarrier **(Gao** et al. **2020).** ^236^ Reproduced from ref 236 under a Creative Commons Attribution License.

However, lack of in vivo data limits conclusions about biodistribution, tumor penetration, and systemic effects. Precise control of laser intensity and exposure time will be critical; small changes may cause collateral tissue heating and or damage. Gold–thiol bond stability in the human body and long-term retention remains untested. Different tumor types may respond variably based on tumor density, vascular perfusion, or antioxidant defenses. Continuous-wave NIR may not penetrate deeply in solid tumors, limiting applicability to shallow or easily accessible tumors. The synthesis of this multifaceted approach leads to high costs and complexity, as well as storage issues hindering the scalability of this strategy that it needs to make a case for its validity in clinical trial testing.^236^

Another example involved a porous polydopamine nanoparticle core for photothermal conversion, a thermoresponsive lipid shell loaded with chemotherapy drugs, and the surface coated with a cancer cell membrane for immune rejection evasion and biomimetic targeting. Upon NIR laser irradiation at 808 nm, the core generates localized heating that disrupts the lipid shell, triggering drug release directly at the tumor site while also inducing photothermal destruction of cancer cells.

This system demonstrated a high photothermal conversion efficiency (roughly 40%) and maintained stability across multiple irradiation cycles. Drug release was highly temperature-sensitive, with over 80% chemotherapy being released at mild hyperthermia (approximately 43 °C) but minimal release under normal conditions (37 °C).

In vitro experiments showed strong synergistic cytotoxicity in 4T1 breast cancer cells, and in vivo studies confirmed efficient tumor accumulation facilitated by the biomimetic membrane layer leading to approximately 90% tumor growth inhibition with negligible systemic toxicity.

Despite these promising outcomes, the same limitations of scalability remain as an issue. Long-term biodistribution and clearance were not evaluated.

While the system used chemotherapy drug doxorubicin, the design is readily adaptable to metal-based therapeutics such as Pt­(IV) or Ru­(II) complexes, offering potential for translation in metal-complex-mediated combination therapies.^235^

## 10 Conclusions

This review offers an effective summary of the key underlying scientific principles of many strategies that have and can be used to enhance selectivity and specificity of anticancer metal complexes to mitigate systemic toxicity and side effects (a key issue of cisplatin). The strategies discussed were then heavily assessed on the basis of their potency, biocompatibility, biodegradability, selectivity, scalability, and clinical translatability. On the basis of these criteria, the methods were ranked from best to worst.

The exploitation of the EPR effect on its own or with PEGylation boosts the circulation of anticancer metal complexes while also taking advantage of poor lymphatic drainage and leaky vasculature. However, from patient to patient, the EPR effect is pronounced to different extents and there is so active targeting of tumors meaning little tumor accumulation.

Active targeting methods with PEGylation target cancer cells more effectively by exploiting overexpressed enzymes, antigens, and receptors on the surface of cancer cells. Unfortunately, the same issues arise with human tumor variability in how overexpressed these features are patient to patient. The targeting ligands can be unstable in circulation, and adding PEG to resolve this can complicate surface chemistry balance of the metal complex and overcomplicate synthesis, hindering scalability.

The controlled activation of metal complexes in the hypoxic, slightly acidic, high GSH, and reductive tumor microenvironment can be a promising principle behind many metal complex prodrugs via redox-sensitive leaving groups or linkers/spacers (disulfide and azido bonds). Although the pH difference between cancer cells and healthy cells is small, hypoxia activation can be premature if patients have already existing oxidative stress or inflammation in healthy cells that are healing, and GSH elevation can once again vary between patients, hindering clinical trial potential.

The organelle-targeting strategies offer specific and controlled cytotoxicity. On the other hand, cationic TPP for targeting enhanced negative inner membrane potential in cancer cell mitochondria can sometimes hinder solubility and stability in circulation of the metal complex. The synthesis of NLS peptides can be difficult and the quantification of DNA binding/accumulation involves advanced imaging technology that is not readily available in clinical trials. Lysosomal acidity in cancer cells can be hard to effectively target due to other acidic compartments being present. These organelles are present in healthy cells too so unless these strategies are combined with methods that actively deliver the drug to the tumor, then organelle targeting is relatively ineffective on its own.

Hormonal targeting improves biocompatibility and biodegradability, but it can lead to endocrine damage, hormonal imbalances, and promotion of the growth of tumors instead in some cases. The strategy is limited to hormone-dependent cancers.

The use of PDT offers a very controlled method of overcoming drug resistance. The metal complexes remaining inert in darkness and only becoming active when irradiated with light are an effective way to target cancer cells once accumulated. The production of ROS upon light activation causes damage to many organelles and causes apoptosis in various pathways, meaning development of resistance to PDT is unlikely. However, blue-light irradiation can cause damage to surrounding healthy tissue and cannot penetrate deeply. Even when NIR light is used continuously, the therapy is limited to accessible or shallow tumors. PTT has a similar issue, where the heat can damage nearby healthy cells.

Dendrimers, MOFs, liposomes, and polymeric nanoparticles offer a multifaceted approach with high modifiability. The surface of these carriers can be functionalized with PEG and targeting ligands, while the core can be cleavable once inside the tumor microenvironment releasing the encapsulated or conjugated metal complex once inside the cancer cell. This selectivity comes at a cost of long-term retention of MOFs and polymeric nanocarriers in the liver and spleen due to lack of biodegradability as well as low scalability/complex synthesis. Liposomes offer a more natural biocompatible and biodegradable system, but they are known to have low encapsulation and storage efficiency or stability. Each method used individually has its own promising advantages, some more than others; however, those that do stand out (PDT, nanocarriers, targeting ligands) usually all suffer from scalability or long-term lack of biodegradability.

Therefore, the most recent generations of cancer-selective complexes often combine these methods. For example, PEGylated nanocarriers with tumor-targeting ligand surfaces and GSH-cleavable release of redox-sensitive or organelle-targeting metal complex prodrugs or PTT to cause nanoparticles to release theranostic metal complexes. These combination strategies are more selective than traditional chemotherapy, but their scalability and large-scale clinical use remain a challenge to this day.

### 10.1 Future Suggestions

Cancer cell variability is the main feature that makes treating this disease so difficult. Tumors can vary from their surfaces to their intracellular environments. This variability often calls for extremely specific and sophisticated metal complex design that achieves selectivity, biocompatibility, and biodegradability while also being scalable to obtain significant clinical trial success. Cisplatin, despite its various side effects and drawbacks, is extremely simple to synthesize and relatively cost-effective, making it the gold standard metal complex for causing apoptosis in rapidly dividing cells such as cancer cells. To find an alternative that is more selective while also being more scalable, certain strategies could be tested in the future. The next generation of anticancer metal complexes could utilize metals that are even more cost-effective than platinum such as cobalt or copper with copper being very biocompatible. If platinum is used, then preferably Pt­(IV) prodrug complexes can be used with group ligands that are only cleaved once inside the reductive tumor microenvironment. Octahedral geometry should be favored, as it allows for more tunability. For example, a platinum­(IV) prodrug with a bidentate 2–2-disulfide bridged bipyridine ligand that will leave when inside elevated GSH tumors. The other 4 coordination spots can be taken up by redox-sensitive ligands such as 1,10-phenanthroline-5,6-dione where the carbonyls are reduced to hydroxyls inside the tumor environment, altering the ligands from electron-withdrawing to electron-donating, which changes the absorption and emission signals, allowing live-tumor detection and response monitoring. A theranostic agent like this will preferably shift toward NIR so that the change in the signal can be seen easily even in deep tissue tumors. Not much continuous use of NIR is needed as it is only used to observe localization and confirm damage rather than being the primary driver of cytotoxicity like in PDT. A complex like this is an effective imaging agent of tumors specifically and binds to DNA more readily in cancer cells due to elevated GSH converting the prodrug into its active Pt­(II) species. To avoid overcomplicating surface chemistry of the metal complex, a liposome can be functionalized with a 50:50 ratio of PEG and targeting ligand such as a small easily synthesizable PSMA-targeting peptide rather than a large antibody. Liposomes are less likely to have long-term retention issues that MOFs have and small peptides such as GUL are more scalable synthetically than large proteins. The liposome can include a disulfide shell or the PEG can include a cleavable disulfide bridge to ensure the carrier not only accumulates but also releases the theranostic prodrug when inside the tumor where the complex will be activated. This Pt­(IV) complex only adds two synthetic steps to traditional Pt­(IV) prodrug dihydroxyplatin (react with the cleavable leaving group ligand and then with the redox sensitive ligand), helping to improve simplicity and scalability.

## Supplementary Material



## Notes

Additional experimental data including cell images, cytotoxicity charts, absorption emission plots, and in vivo imaging of tumor responses to treatment and references for data and abbreviations of acronyms used in manuscript (PDF)
