# Supplementary material for: Strategies for Enhancing Selectivity in Anticancer Metal Complexes
Source: ACS Omega. 2026 May 22;11(22):31972–2001. doi: 10.1021/acsomega.5c11333 (PMC13261472; doi:10.1021/acsomega.5c11333)
Supplement: Supplementary file 1 [file ao5c11333_si_001.pdf]

Supporting Information

Strategies for Enhancing Selectivity in Anticancer Metal Complexes

Paolo R. Butcher*[1] and Daniel Sykes[1]

[1] School of Human Sciences, London Metropolitan University, 166–220 Holloway Road, London N7 8DB, UK

**Corresponding author email:* pa2016b@gmail.com

Description of Contents

Supporting information: Additional experimental data including cell images, cytotoxicity charts, absorption emission plots, and in vivo imaging of tumor responses to treatment. References for data and abbreviations of acronyms used in manuscript.

Introduction - Supplementary Data

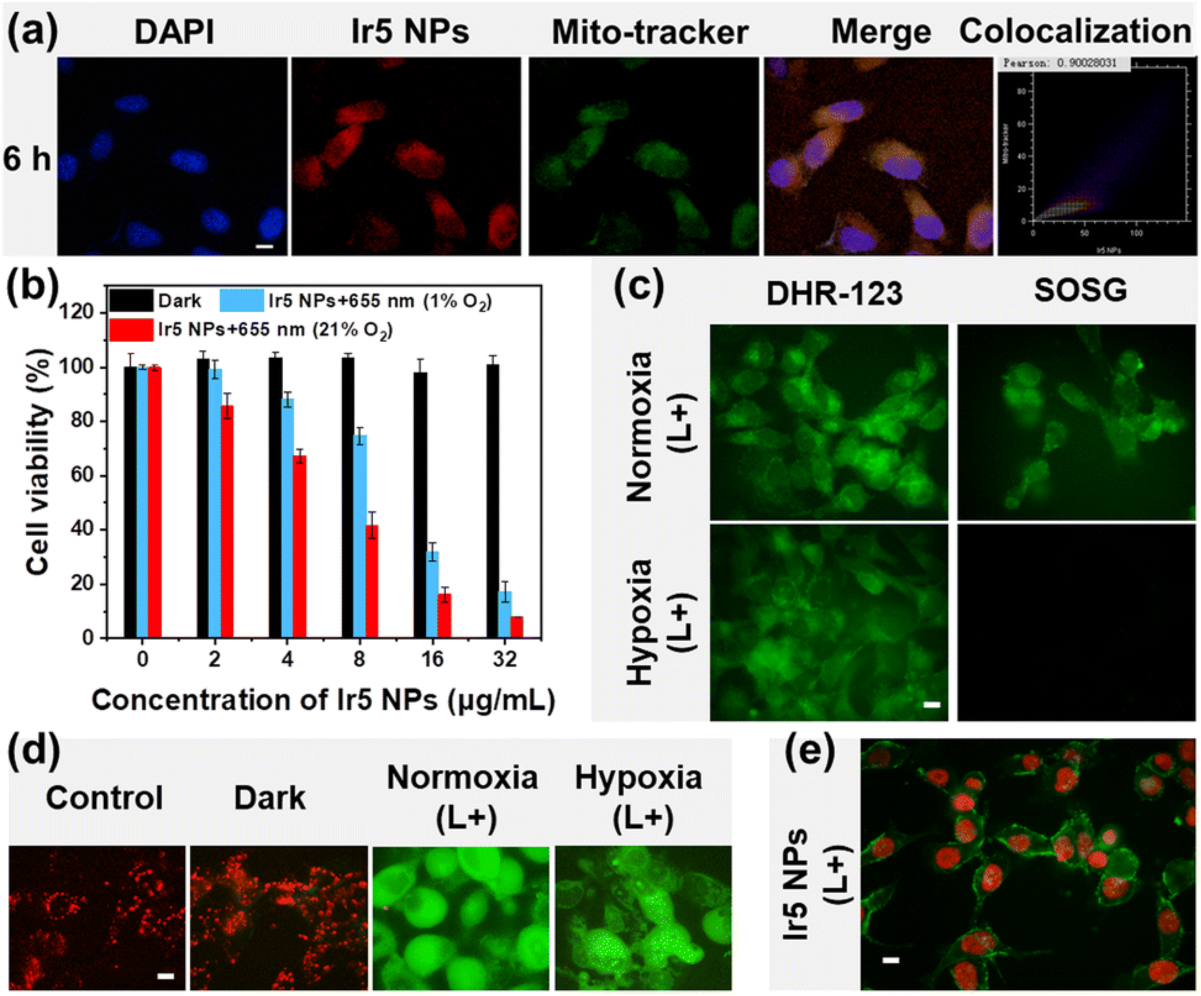

Figure S1. *(a) CLSM images of 4T1 cells after incubation with Ir5 NPs and colocalization assay of Ir5 NPs with MitoTracker Green. (b) Relative viability of 4T1 cells after co-incubation with Ir5 NPs under different conditions. Confocal fluorescence images for the detection of ROS generation (c), the detection of MMP via JC-1 dye assay (d) and cell death detection by dual fluorescence of Annexin V-FITC/PI staining (e) in 4T1 cells treated with Ir5 NPs under different conditions. Scale bar = 10 μm.*

Ir complex used in PDT, generating reactive oxygen species, causing apoptosis, Liu et al., 2024, 2024. [(1)]

Reproduced with permission from [Ref. 1]. Copyright 2024 Royal Society of Chemistry. Creative Commons Attribution 3.0 Unported Licence.

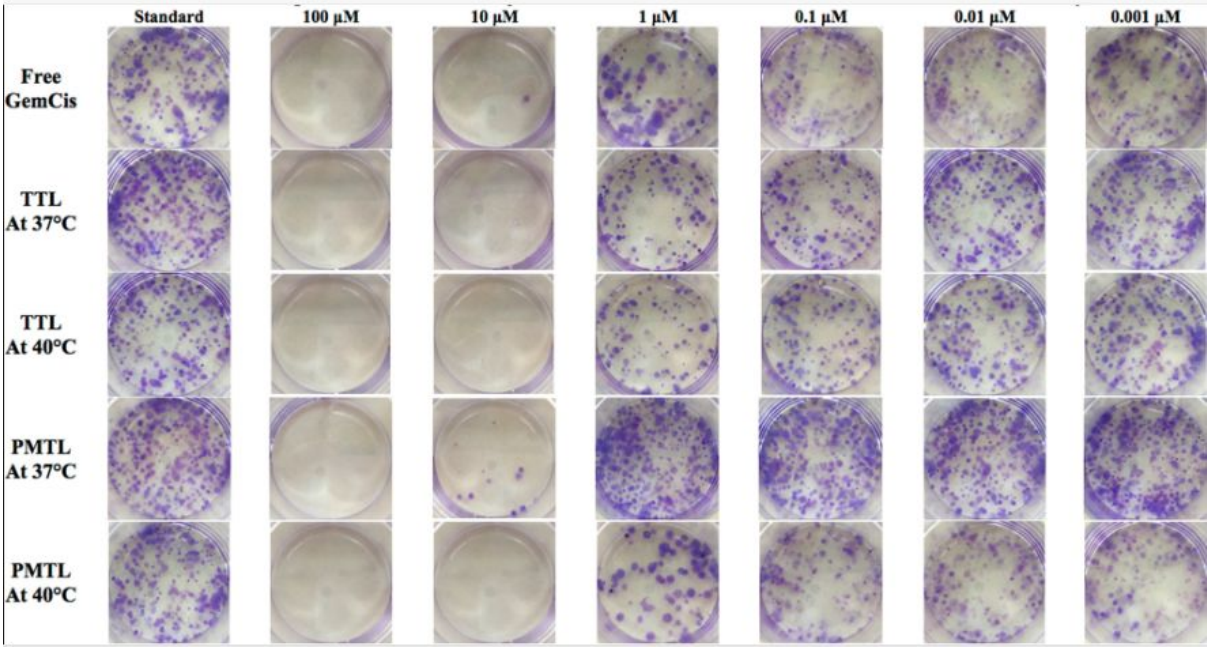

Figure S2. *Digital photographs of the clonogenic assay of MiaPaCa-2 cells performed with the combination of free GemCis, loaded in TTL and PMTL below and above the LCST for 14 consecutive days in 6-well plates (the diameter of each well is approximately 34.8 mm).*

*Liposome nanoparticles used for drug delivery**,** Emamzadeh, Emamzadeh and Pasparakis,* 2019. **[(2)]**

Reproduced from [Ref. 2]. Copyright 2019 American Chemical Society.

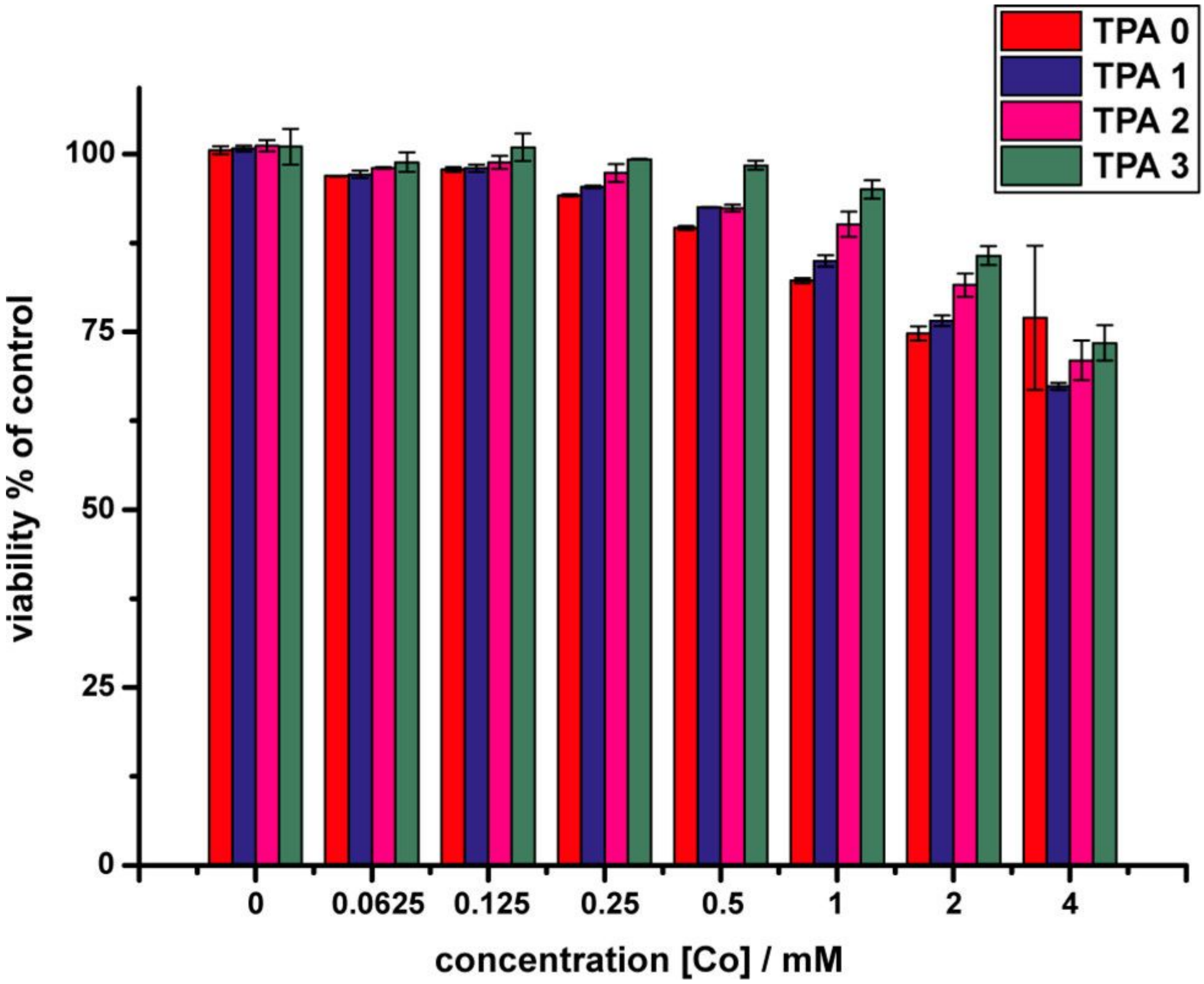

Chart S1. *Cytotoxicity of TPAx complexes measured by alamarBlue assay (DLD-1 cells, 24 h incubation with TPAx, followed by 4 h incubation with alamarBlue). Cell viability normalized to controls incubated with PBS vehicle alone.*

Co complexes with redox sensitive carbonyls that can be used for detection of hypoxic tumor environments via a change in the emission signal, O'Neill et al., 2017. **(3)**

Reproduced from [Ref. 3]. Copyright 2017 American Chemical Society.

Passive Targeting - Supplementary Data

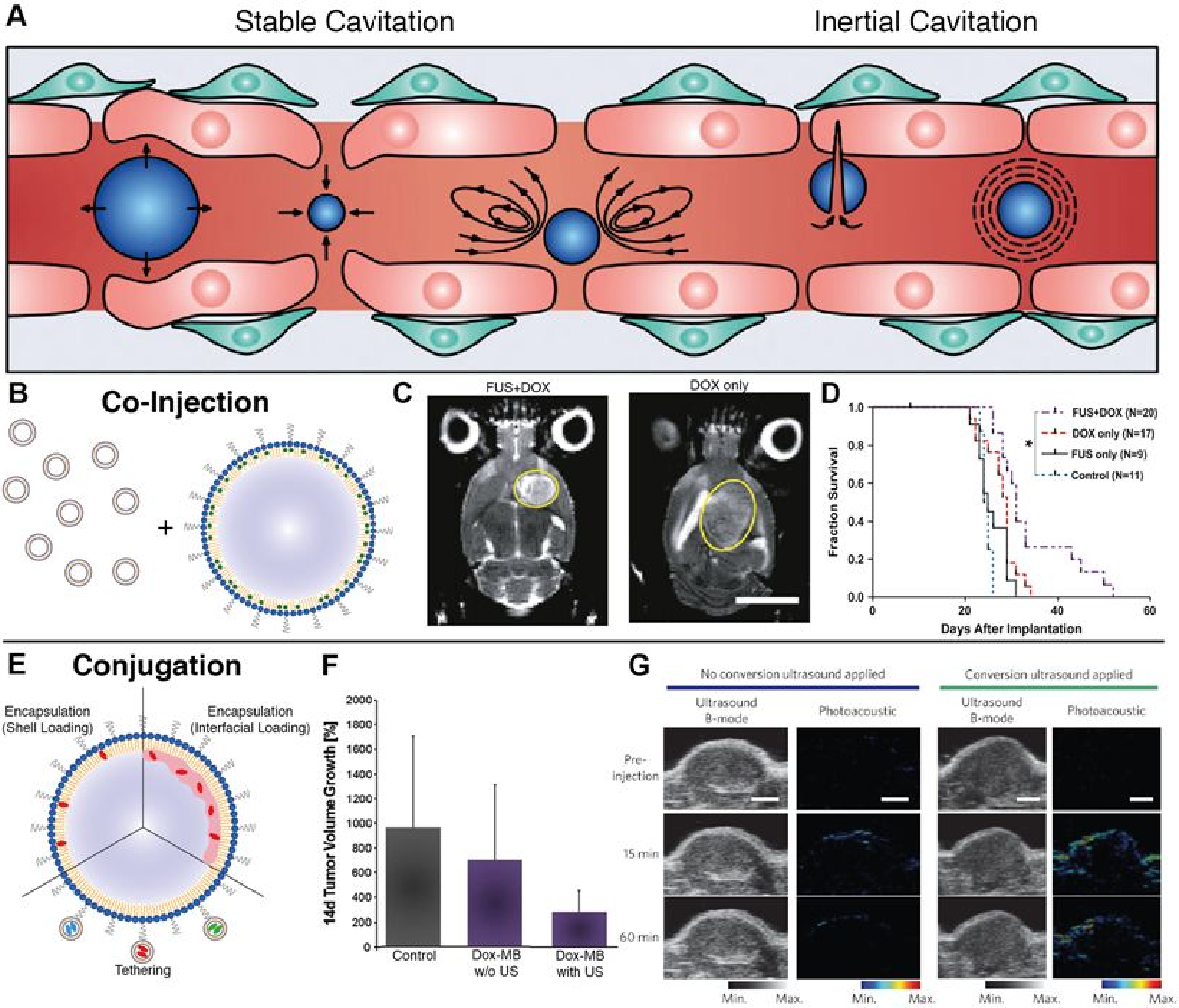

Figure S3. *Cavitation-based mechanisms by which ultrasound can enhance nanomedicine delivery, including transient disruption through stable cavitation and destructive opening through inertial cavitation (adapted from. B. Co-injection involves simultaneous administration of microbubbles and nanoparticles with ultrasound priming to improve delivery. C-D. Application of microbubble-enhanced focused ultrasound and pre-administered liposomal doxorubicin on rat brains bearing 9L glioma xenografts results in tumor size reduction and prolonged survival compared to doxorubicin only controls (adapted from. E. Conjugation encompasses both nanoparticle tethering and agent encapsulation to produce a highly co-localized platform for delivery improvement. F. The combination of doxorubicin-loaded microbubbles and ultrasound achieve enhanced therapeutic reduction in DSL6A pancreatic xenograft size compared to drug-loaded bubbles alone. G. In situ microbubble-to-nanoparticle conversion of porphyrin-lipid microbubbles upon ultrasound irradiation leads to enhanced nanoparticle delivery as measured by photoacoustic imaging,*

A comparison between EPR sensitive tumours that have poor vasculature/lymphatic drainage and insensitive tumours that have less exploitable EPR effect, Dhaliwal & Zheng, 2019. **(4)**

Reproduced with permission from [Ref. 4]. Copyright 2019 PubMed Central.

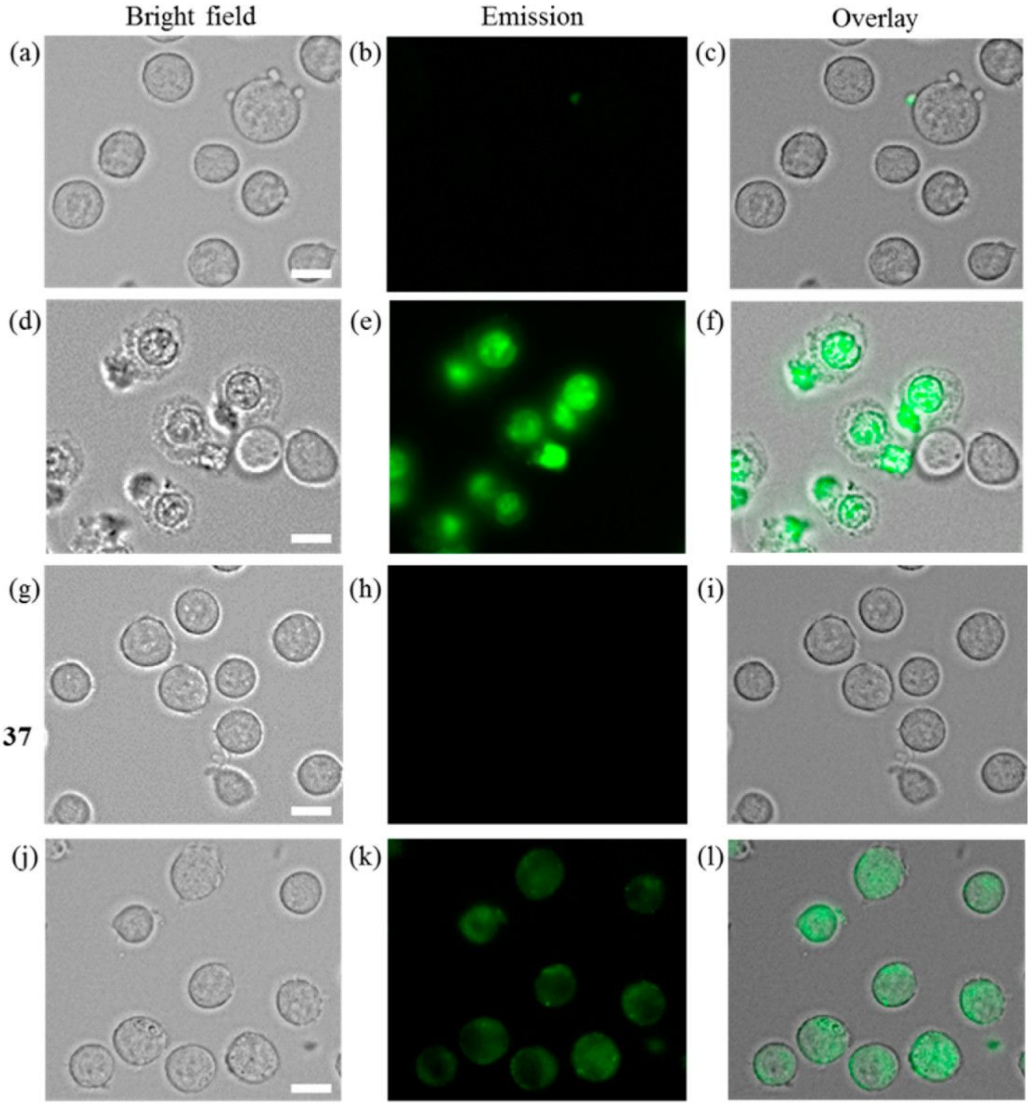

Figure S4. *Luminescence microscopy images (Biorevo, BZ-9000, Keyence) of Jurkat cells treated with Ir complexes 6i (75 μM), 7b (50 μM), 21 (50 μM) + deprotected KKGG peptide 37 (150 μM), and 11 (225 μM) for 1 h at 37 °C. (a) Bright field image of 6i, (b) emission image of 6i, (c) overlay image of (a) and (b), (d) bright field image of 7b, (e) emission image of 7b, (f) overlay image of (d) and (e), (g) bright field image of 21 + 37, (h) emission image of 21 + 37, (i) overlay image of (g) and (h), (j) bright field image of 11, (k) emission image of 11, (l) overlay image of (j) and (k). Excitation at 377 nm for Ir complexes and an exposure time ca. 0.02 s for (b), (e), (h), and 0.25 s for (k). Scale bar (white) = 10 μm.*

Amphiphilic iridium (III) complex that can be used to exploit EPR effect, Hisamatsu et al. 2015. [(5)]

Reproduced from [Ref. 5]. Copyright 2015 American Chemical Society.

Active Targeting - Supplementary Data

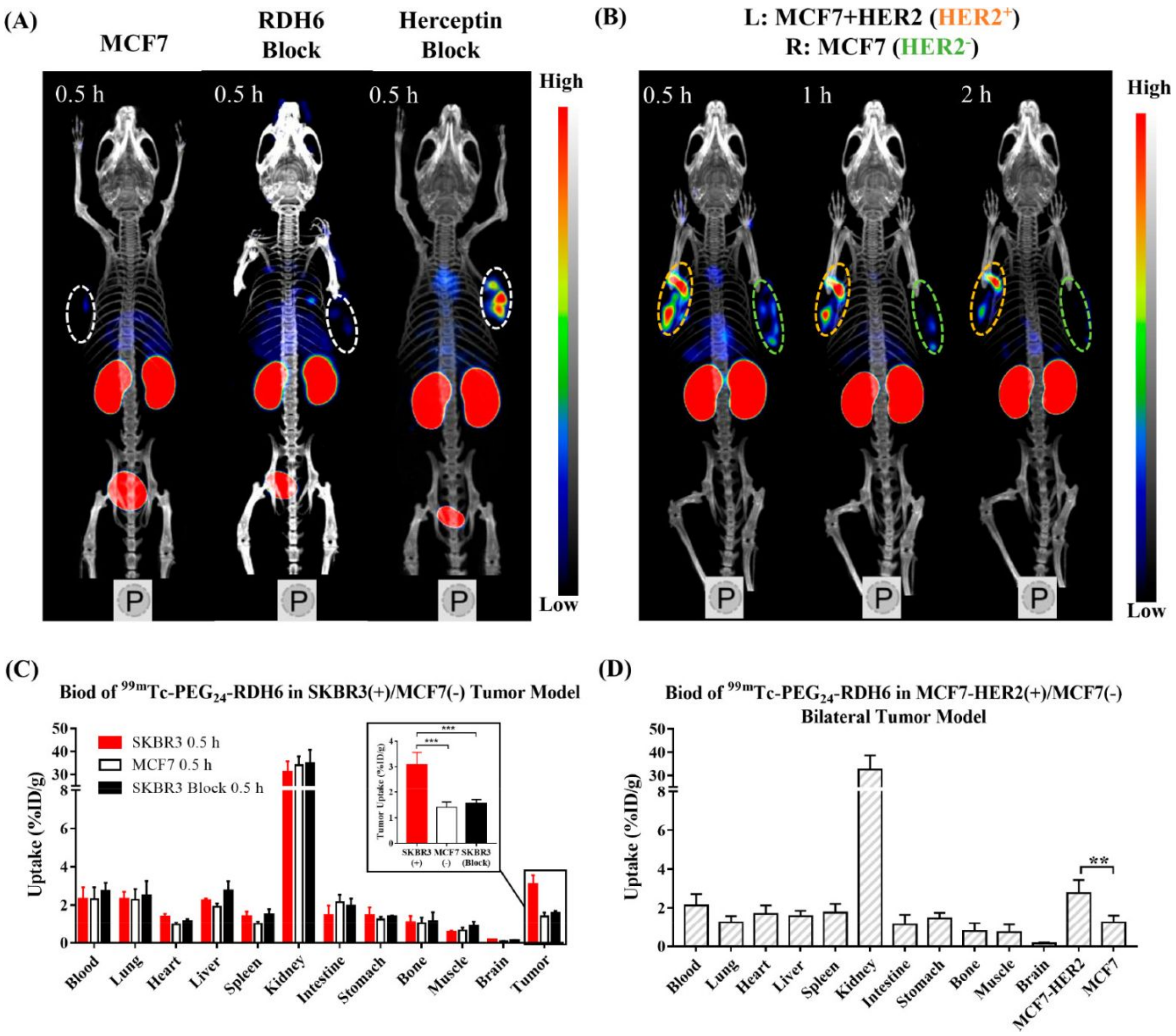

Figure S5. *Representative nanoScanSPECT/CT images of ^{99m}Tc-PEG$_{24}$-RDH6 in MCF7 tumor model and in SKBR3 tumor model with coinjection of excess cold RDH6 peptide or Herceptin at 0.5 h p.i. (A), and in MCF7-HER2/MCF7 bilateral tumor model at 0.5, 1, and 2 h p.i. (B). Biodistribution of ^{99m}Tc-PEG$_{24}$-RDH6 in MCF7 tumor model at 0.5 h p.i. and in SKBR3 tumor model with and without the blocking dose of cold RDH6 peptide (C), and in MCF7-HER2/MCF7 bilateral tumor model at 0.5 h p.i. (D) ($P < 0.05$, *; $P < 0.005$, **; $P < 0.0005$, ***).*

Technetium complex with targeting peptides linked to ligands, Du et al., 2020. **(6)**

Reproduced from [Ref. 6]. Copyright 2020 American Chemical Society.

Stimuli-Responsive Activation - Supplementary Data

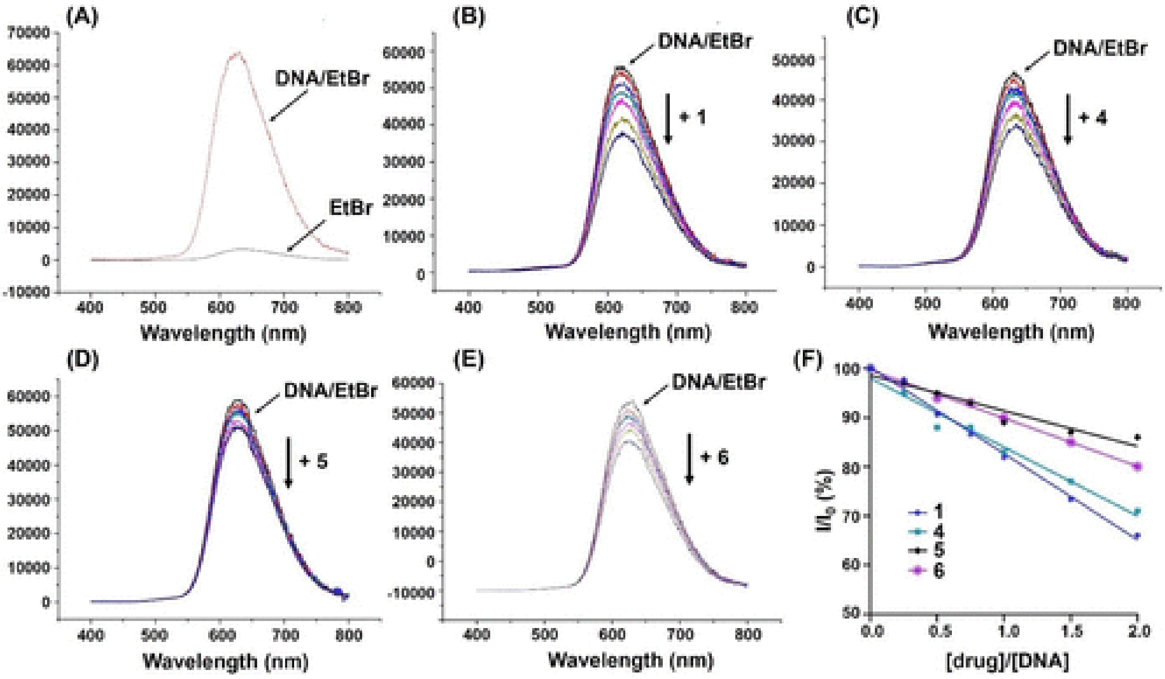

Figure S6. *(A) Fluorescent spectra of salmon sperm DNA/EtBr and EtBr in the absence of DNA. (B–E) Changes in fluorescent spectra of the salmon sperm DNA/EtBr complex upon sequential addition of complex (B) 1, (C) 4, (D) 5, and (E) 6 in the DMSO/H_2O (4:6, v/v) solvent system. The ratio of the drug to DNA was varied from 1:0 to 2:1. (E) Half-reciprocal plot of I/I_0 vs drug–DNA concentration ratio (fold change).*

Redox sensitive Pt/Pd complexes, Romashev et al. 2022. [(7)]

Reproduced from [Ref. 7]. Copyright 2022 American Chemical Society.

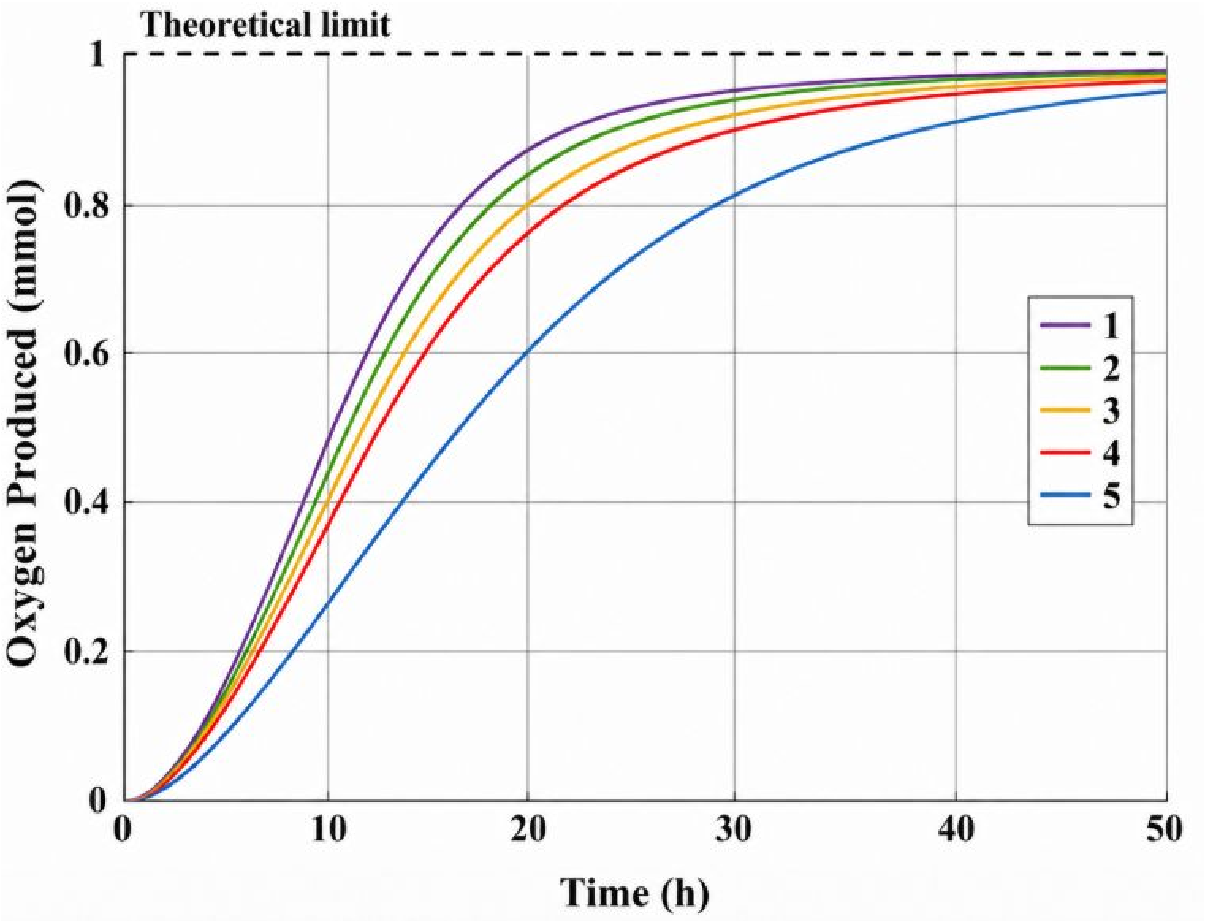

Figure S7. *O2 evolution traces of complexes 1–5 at 50 µM catalyst and 0.36 M CAN (final volume = 11 mL). The total amount of O2 produced with all catalysts is consistent with the stoichiometric limit of the added CAN.*

Iridium complexes utilizing sulfonamides, Li, M. & Bernhard, S., 2017. [8]

Redrawn using Excel based on literature from [Ref. 8].2017 Elsevier

External Stimulus-Controlled - Supplementary Data

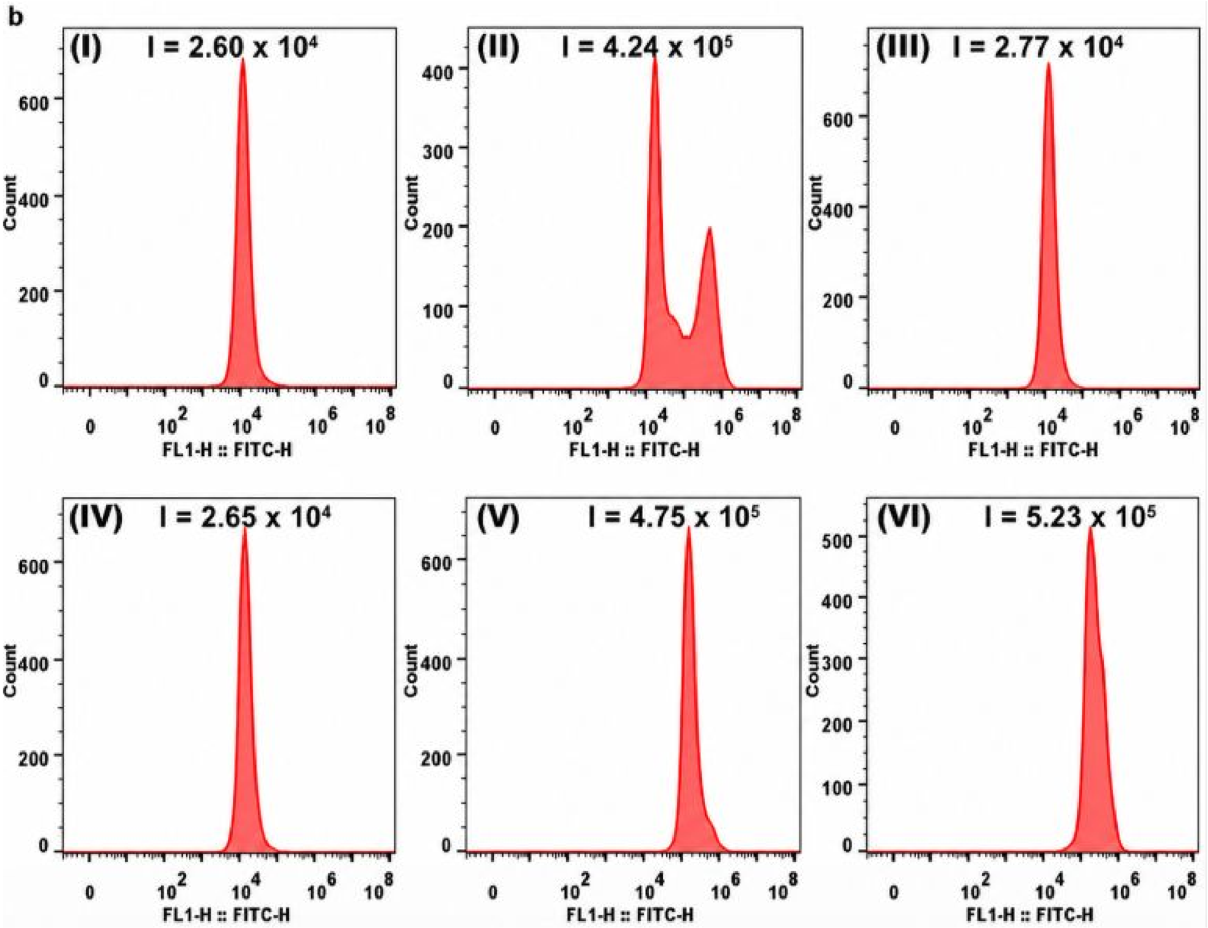

__Figure S8.__ (a) IC_{50} concentration of Ir1 or Ir2 in the absence or presence of irradiation. (b) Assay of DCF fluorescence intensity after 24 h exposure of A549 cells (I) to ROSUP (II), IC_{50} concentration of Ir1 (III, V) or Ir2 (IV, VI) in the absence or presence of irradiation.

PDT using light activated iridium complexes to produce ROS resulting in apoptosis, Li et al., 2022. [9]

Redrawn using Excel based off literature from [Ref. 9]. 2022 Elsevier

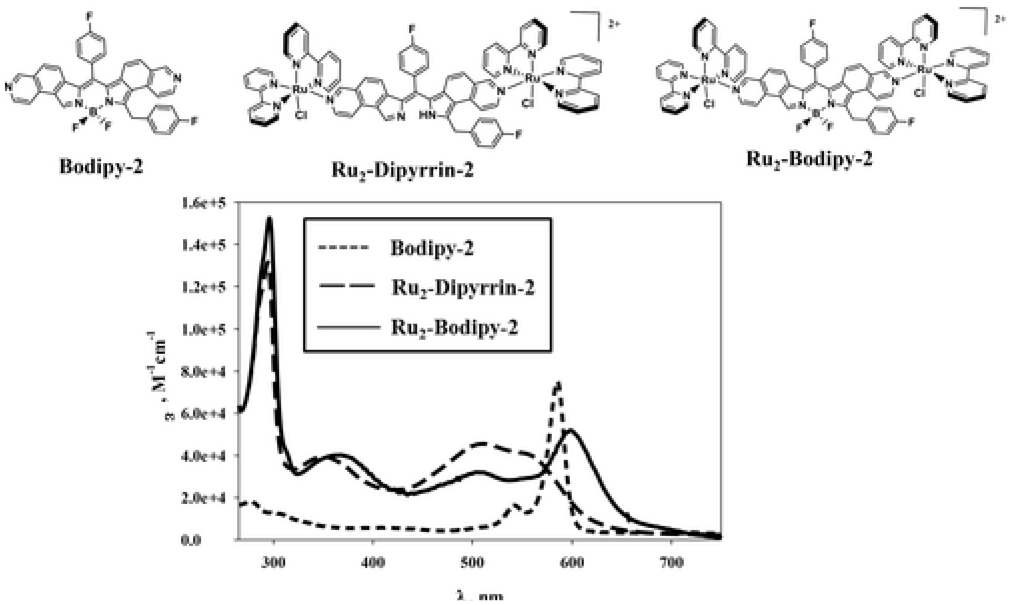

Figure S9. *UV–vis spectra of 2, Ru_2-2, and Ru_2-dipyrrin in DCM at RT.*

Polypyridyl Ru (II) complex with tunable photophysical properties via change in R_1 and R_2. Swavey et al. 2017. [(10)]

Reproduced from [Ref. 10]. Copyright 2017 American Chemical Society.

Delivery Systems - Supplementary Data

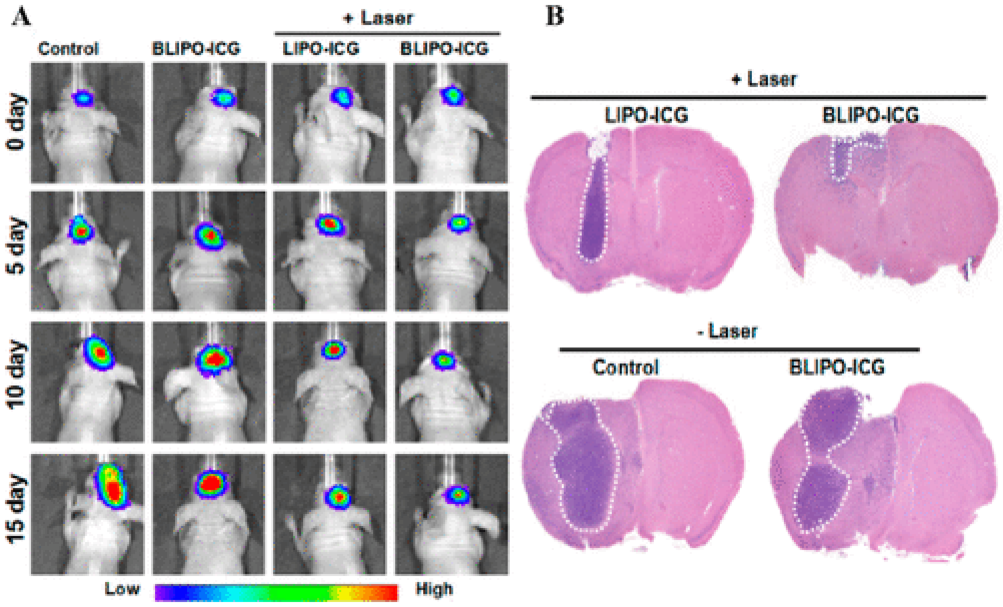

Figure S10. Early fluorescent imaging detection of liposomes in an orthotopic glioma model. (A) Representative bioluminescent images of C6-Luc glioma-bearing mice in different groups. (B) H&E staining of brain sections of orthotopic glioma-bearing mice in all groups.

The modifications that can be made to liposomes surfaces, improving selectivity, Li et al., 2020. [(11)]

Reproduced from [Ref. 11]. Copyright 2020 American Chemical Society.

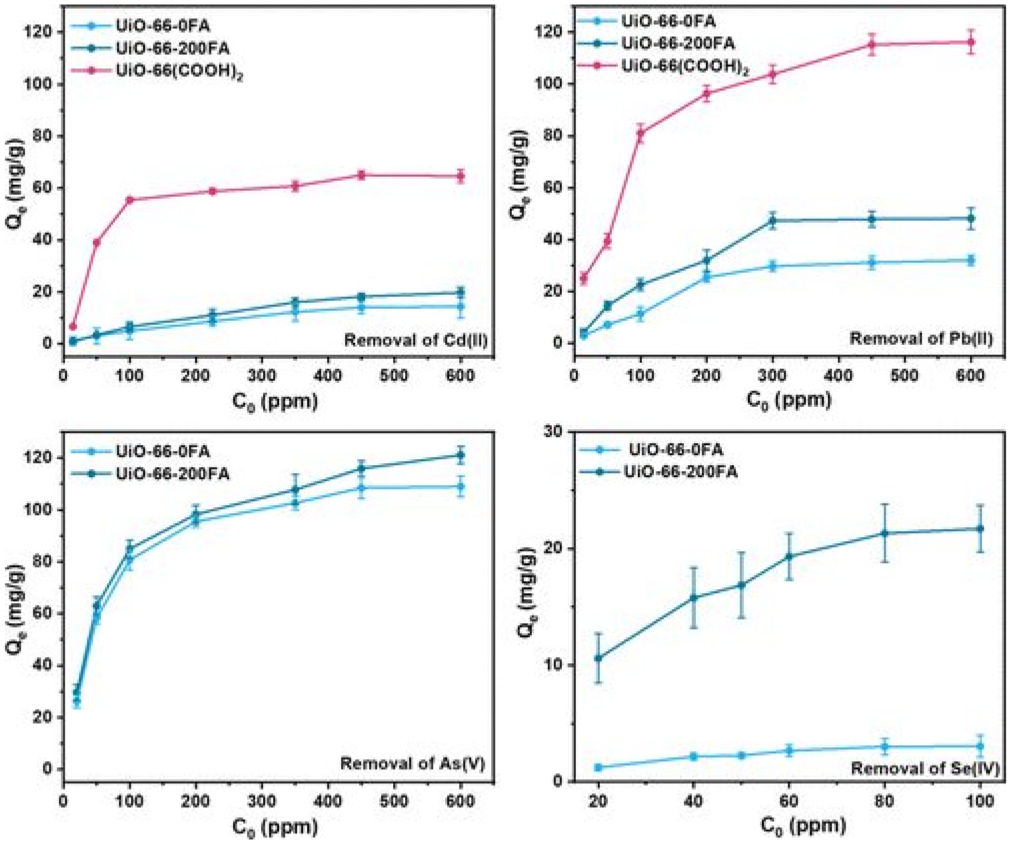

Figure S11. *Adsorption isotherms of Pb(II) and Cd(II) onto UiO-66(COOH)$_2$ and of Pb(II), Cd(II), As(V), and Se(IV) onto UiO-66-200FA and UiO-66-0FA.*

Zr-based MOFs (UiO-66) that can be used to hold M^{2+} metals such as cis platin, Jrad *et al.* 2022. **(12)**

Reproduced from [Ref. 12]. Copyright 2022 American Chemical Society.

References

(1) Liu, S., Wang, Z., Wu, Z., Chen, H., Zhu, D., Li, G., Yan, M., Bryce, M.R. and Chang, Y., 2024. Long-wavelength triggered iridium(III) complex nanoparticles for photodynamic therapy against hypoxic cancer. Chemical Communications. Found at: https://doi.org/10.1039/D4CC03501A. Accessed (04/03/25).

(2) Emamzadeh, M., Emamzadeh, M. and Pasparakis, G., 2019. Dual controlled delivery of gemcitabine and cisplatin using polymer-modified thermosensitive liposomes for pancreatic cancer. ACS Applied Bio Materials, 2(3), pp.1298–1309. Found at: https://doi.org/10.1021/acsabm.9b00007. Accessed (05/03/25).

(3) O'Neill, E.S., Kaur, A., Bishop, D.P., Shishmarev, D., Kuchel, P.W., Grieve, S.M., Figtree, G.A., Renfrew, A.K., Bonnitcha, P.D. and New, E.J., 2017. Hypoxia-responsive cobalt complexes in tumor spheroids: Laser ablation inductively coupled plasma mass spectrometry and magnetic resonance imaging studies. Inorganic Chemistry, 56(16), pp.9860–9868. Found at: https://doi.org/10.1021/acs.inorgchem.7b01368. Accessed (08/03/2025).

(4) Dhaliwal, A. & Zheng, G. (2019). Improving accessibility of EPR-insensitive tumor phenotypes using EPR-adaptive strategies: Designing a new perspective in nanomedicine delivery. Theranostics, 9(26), 8091–8108. Found at: https://doi.org/10.7150/thno.37204 . Accessed (14/03/2025).

(5) Hisamatsu, Y., Shibuya, A., Suzuki, N., Suzuki, T., Abe, R. and Aoki, S., 2015. Design and synthesis of amphiphilic and luminescent tris-cyclometalated iridium(III) complexes containing cationic peptides as inducers and detectors of cell death via a calcium-dependent pathway. Bioconjugate Chemistry, 26(5), pp.857–879. Found at: https://doi.org/10.1021/acs.bioconjchem.5b00095. Accessed (20/03/2025).

(6) Du, S., Luo, C., Yang, G., Gao, H., Wang, Y., Li, X., Zhao, H., Luo, Q., Ma, X., Shi, J. and Wang, F., 2020. Developing PEGylated Reversed D-Peptide as a Novel HER2-Targeted SPECT Imaging Probe for Breast Cancer Detection. Bioconjugate Chemistry, 31(8), pp.1971–1980. Found at: https://doi.org/10.1021/acs.bioconjchem.0c00334. Accessed (03/04/2025).

(7) Romashev, N. F., Abramov, P. A., Bakaev, I. V., Fomenko, I. S., Samsonenko, D. G., Novikov, A. S., Tong, K. K. H., Ahn, D., Dorovatovskii, P. V., Zubavichus, Y. V., Ryadun, A. A., Patutina, O. A., Sokolov, M. N., & Babak, M. V. (2022). Heteroleptic Pd(II) and Pt(II) complexes with redox-active ligands: Synthesis, structure, and multimodal anticancer mechanism. Inorganic Chemistry, 61(1), 75–89. Found at: https://doi.org/10.1021/acs.inorgchem.1c03314. Accessed (14/04/2025).

(8) Li, M. & Bernhard, S., 2017. Synthetically tunable iridium(III) bis-pyridine-2-sulfonamide complexes as efficient and durable water oxidation catalysts. Catalysis Today, 280, pp.19–27. Found at: https://doi.org/10.1016/j.cattod.2016.11.027. Accessed (15/04/2025).

(9) Li, W., Shi, C., Wu, X., Zhang, Y., Liu, H., Wang, X., Huang, C., Liang, L., & Liu, Y. (2022). Light activation of iridium(III) complexes driving ROS production and DNA damage enhances anticancer activity in A549 cells. Journal of Inorganic Biochemistry, 236, 111977. Found at: https://doi.org/10.1016/j.jinorgbio.2022.111977. Accessed (01/05/2025).

(10) Swavey, S., Kumar, S.V. and Erb, J., 2017. Ruthenium(II) polypyridyl complexes coordinated directly to the pyrrole backbone of π-extended boron dipyrromethene (Bodipy) dyes: Synthesis, characterization, and spectroscopic and electrochemical properties. Inorganic Chemistry, 56(17), pp.10664–10673. Found at: https://doi.org/10.1021/acs.inorgchem.7b01630. Accessed (02/05/2025).

(11) Li, J., Tan, T., Zhao, L., Liu, M., You, Y., Zeng, Y., Chen, D., Xie, T., Zhang, L., Fu, C. & Zeng, Z. (2020) 'Recent advancements in liposome-targeting strategies for the treatment of gliomas: A systematic review', ACS Applied Bio Materials, 3(9), pp. 5500–5528. Found at: https://doi.org/10.1021/acsabm.0c00705. Accessed (16/05/2025).

(12) Jrad, A., Damacet, P., Yaghi, Z., Ahmad, M. and Hmadeh, M., 2022. Zr-based metal–organic framework nanocrystals for water remediation. ACS Applied Nano Materials, 5(8), pp.10795–10808. Found at: https://doi.org/10.1021/acsanm.2c02128. Accessed (24/05/2025).

**Abbreviation**	**Definition**
CT	Computed Tomography

DNA	Deoxyribonucleic Acid
DUPA	DUPA, or 2-[3-(1,3-dicarboxypropyl)ureido]pentanedioic acid
EGFR	Epidermal Growth Factor Receptor
EPR	Enhanced Permeability and Retention
FADH	Flavin adenine dinucleotide
FRET	Förster Resonance Energy Transfer
G4	G-quadruplex
GSH	Glutathione
GUL	Glu-Urea-Lys
HER2	Human Epidermal Growth Factor Receptor 2
HIF	Hypoxia Inducible Factor
HOMO	Highest Occupied Molecular Orbital
LUMO	Lowest Unoccupied Molecular Orbital
MMP	Matrix Metalloproteinase
MOFs	Metal–Organic Frameworks
MRI	Magnetic Resonance Imaging
MYC	Myelocytoma
NADPH	Nicotinamide Adenine Dinucleotide Phosphate (Reduced form)
NAMI-A	Imidazolium-trans-tetrachloro(dimethylsulfoxide)imidazoleruthenium(III))
NIR	Near-Infrared
NLS	Nuclear Localization Signal
NMR	Nuclear Magenetic Resonance
PAMAM	Polyamidoamine
PDT	Photodynamic Therapy
PEG	Polyethylene Glycol
PH	Potential of Hydrogen
PKKKRKV	Proline-lysine-lysine-lysine-arginine-lysine-valine

PSMA	Prostate-Specific Membrane Antigen
PTT	Photothermal Therapy
RGD	Arginine-Glycine-Aspartic acid peptide
ROS	Reactive Oxygen Species
SPIONs	Superparamagnetic Iron Oxide Nanoparticles
TPP	Triphenylphosphonium
VEGFR	Vascular Endothelial Growth Factor
